# Proceedings of the Fifteenth International Society of Sports Nutrition (ISSN) Conference and Expo: Clearwater Beach, FL, USA. 7-9 June 2018

**DOI:** 10.1186/s12970-018-0256-5

**Published:** 2018-11-06

## A1 Effects of a short-term vegan diet on fitness and body composition

### Michael Dahlinghaus, Drake Berberet, John Rehfeld

#### Department of Kinesiology, St. Ambrose University, Davenport, IA, 52803, USA

##### **Correspondence:** Michael Dahlinghaus (dahlinghausmichaelf@sau.edu)

**Background**

Vegan diets refrain from the consumption of all animal products. People may choose a vegan diet for health, performance, or ethical concerns. While the adoption and popularity of a vegan diet has increased among the general population and fitness communities, there has been little research documenting its effect on general fitness (max strength, cardiorespiratory endurance, muscular endurance) and body composition. The purpose of this randomized controlled study was to study the effects of adopting a vegan diet on general fitness performance and body composition in an active population.

**Methods**

Twelve physically active college students volunteered for this study. The respondents were randomly and equally divided into two groups: an experimental (vegan) and control group. Each participant was instructed to track their diet for three days prior to pre-testing, as well as during the experiment using myfitnesspal software. Both groups underwent a pre fitness and body composition assessment. This assessment included height, weight, blood pressure (BP), body fat percentage, one repetition maximum (1RM) back squat, 1RM bench press, push up test to failure (PUT), and a Vo2 max test (1.5 mile run). The experimental group was then instructed to follow an ad libitum vegan diet for three weeks which eliminated all animal products from their normal diet. The control group maintained their normal diet. Both groups were instructed to continue their normal exercise program (exercising >3x a week). After three weeks, both groups underwent a post-test consisting of the same initial assessments. A paired sample t-test was used to investigate statistically significant differences between the pre and post-tests for the experimental and control group.

**Results**

For the experimental group, mean weight decreased significantly by 3.4lbs (P<.05). There was also a significant decrease in mean caloric (422.5), protein (32.8), and fat (32.9) intake (P < .05). No statistically significant differences were seen for the control group mean values.

**Conclusion**

Based upon this study, a short term ad libitum vegan diet decreased total body weight with no impact on fitness scores.

## A2 Pilot clinical trial to evaluate the effect of Fenugreek Saponins on physical fitness

### Sreejayan Nair^1^, Derek Smith^2^, Scarlet L. Barnes^2^, Boyi Dai^2^, Rama Nair^3^, Debasis Bagchi^4,5^, Anand Swaroop^5^

#### ^1^University of Wyoming, School of Pharmacy, College of Health Sciences, Laramie, WY, 82071, USA; ^2^University of Wyoming, Department of Kinesiology and Health, College of Health Sciences, Laramie, WY, 82071, USA; ^3^Nutriwyo LLC, 1938 Harney St, Laramie, WY 82072, USA; ^4^University of Houston, College of Pharmacy, Houston, TX, 77004, USA; ^5^Cepham Inc, Piscataway Township, NJ, 07701, USA

**Background and objective**

Previous studies from our lab demonstrated that a saponin rich isolate from the spice fenugreek (*Trigonella foenum-graecum L. Leguminoseae*) improved glucose tolerance, insulin sensitivity and cardiometabolic parameters while mitigating hepatic steatosis in diabetic mice. Furthermore, in a pilot, randomized clinical study, we found that obese, insulin-resistant subjects receiving fenugreek saponins exhibited improved insulin sensitivity as evidenced by a reduced homeostatic model of insulin resistance (HOMA-IR) index. The current study was aimed at evaluating the efficacy of fenugreek saponins in improving lean body mass, cardiorespiratory endurance and muscle strength in healthy human subjects.

**Research Design and Methods**

A prospective, double blind, randomized trial was performed in 40 healthy male human subjects (24.02±3.9 years) who received fenugreek saponin or placebo capsules (250 mg, twice daily) for a 12-week period. Prior to and following intervention, hand-grip, body fat mass/lean mass/fat distribution, upper and lower body strength and maximal graded exercise stress testing were determined using a digital hand dynamometer, dual-energy X-ray absorptiometer (DEXA), force plate, and treadmill with open-circuit spirometry, respectively. Clinical chemistry, free and total testosterone and C-reactive proteins were determined in the serum samples.

**Results**

No changes were observed in grip-strength, jump height, peak jump force and push-up force between the treatment or placebo groups following intervention. Similarly, measures of cardiorespiratory endurance (resting heart rate, maximal heart rate, total exercise test time, VO_2_max, peak and submaximal respiratory exchange ratio) and systolic and diastolic blood pressures were similar between groups and unchanged in both groups following intervention. Similarly, there were no differences or changes in percent fat or fat mass following DEXA analysis. No differences or changes were observed for body mass index, android fat, gynoid fat or the ratio of android to gynoid fat. However, the mean lean mass was significantly elevated in the group receiving fenugreek saponin compared to the placebo group. Total serum testosterone levels were significantly higher in the subjects receiving fenugreek saponins compared to placebo, whereas no changes were observed in free testosterone levels.

**Conclusion**

Short-term intervention with fenugreek saponins increased lean body mass and total testosterone levels. Further long-term studies are warranted to determine if these changes translate to improved physical fitness (i.e., muscle mass, strength, body composition and aerobic capacity).

**Acknowledgments**

This study was supported by a research grant from Cepham Inc, NJ to SN.

## A3 You think you’re fit, but your genes say you’re fat: a polymorphism in the fat mass and obesity-associated (FTO) gene predicts body fat in well-trained athletes

### Sarah D Knafo^1^, Ritishka Kapoor^1^, Jose Antonio^2^, Jaime L Tartar^1^

#### ^1^Department of Psychology and Neuroscience, Nova Southeastern University, Davie, FL, 33024, USA; ^2^Department of Health and Human Performance, Nova Southeastern University, Davie, FL, 33024, USA

##### **Correspondence:** Jose Antonio (ja839@nova.edu)

**Background**

A single nucleotide polymorphism (SNP) in the fat mass and obesity-associated (FTO) gene is a strong predictor of obesity in humans. The FTO SNP (rs1421085) results in a T to C nucleotide change with increased risk for obesity in individuals who carry at least one C allele. The highest expression levels of the FTO enzyme are found in arcuate nucleus of the hypothalamus, which plays a major role in appetite and eating behavior. In agreement, previous work shows that C allele carriers have worse eating behaviors, relative to TT homozygotes. However it is currently unclear if intense aerobic and/or resistance training can reduce the influence of FTO genotype on body composition.

**Materials and Methods**

We tested 108 well-trained individuals that included professional mixed martial arts fighters, elite distance runners, collegiate swimmers, stand-up paddlers as well as a cohort of recreational bodybuilders. Body composition was assessed via the dual-energy x-ray absorptiometry (DXA). Saliva samples were collected in order to genotype participants and quantify cortisol levels.

**Results**

The physical characteristics of the subjects were as follows (mean±SD): body weight 74.4±15.6 kg; bone mineral content 2.8±0.7 kg; fat mass 15.7±5.5 kg; lean body mass 55.9±14.4 kg. We found that C allele carriers had significantly higher fat mass t(107)=3.13, p < 0.01 and a significantly higher body fat percentage t(107)=2.68, p < 0.01, relative to the TT group. No other measures of body composition were associated with the FTO genotype (body mineral density, bone mineral content, or lean body mass). Interestingly, resting cortisol levels were significantly higher in the TT group, relative to the C allele carriers t(107)=-2.37, p < 0.05.

**Conclusions**

Our findings suggest that despite regular exercise training, C allele carriers on the FTO gene are still predisposed to a higher percent body fat as well as fat mass. We further show that increased cortisol is not a likely pathway through which exercise increases weight gain in the C allele carriers.

**Acknowledgements**

This work was supported through a Department of Education grant (P120A140012) awarded to JLT.

## A4 Peanut butter and jelly-belly: a pilot trial

### Cara Axelrod, Cassandra Carson, Anya Ellerbroek, Victoria Burgess, Tobin Silver, Corey Peacock, Jose Antonio

#### Department of Health and Human Performance, Nova Southeastern University, Davie, FL, 33024, USA

##### **Correspondence:** Jose Antonio (ja839@nova.edu)

**Background**

It is known that overfeeding on carbohydrate and fat differ in their effects on body composition in comparison to protein. Data from our lab suggests that it is very difficult to increase fat mass via protein overfeeding (i.e., mainly from protein powder). However, there are no investigations on the effects of overfeeding on a specific food (i.e., peanut butter). Peanut butter is a commonly consumed food among fitness professionals and exercise enthusiasts. Thus, the purpose of this study was to determine how overfeeding on peanut butter affected body composition in a cohort of trained subjects.

**Materials and Methods**

Sixteen healthy exercise-trained men and women participated in this study (mean±SD – age: 30.1±7.8, height cm: 170.8±7.9). Subjects initially recorded their food intake via MyFitnessPal for a period of two weeks prior to coming to the laboratory for baseline body composition assessment. Subsequently, they were instructed to consume 5 jars of peanut butter (Smuckers Natural, 16 oz jar) over the 4-week treatment period. The peanut butter was additional calories above their normal baseline intake. Body composition and total body water was assessed with the Bod Pod and Impedimed. Data was analyzed using a t-test. Data is expressed as the mean±SD.

**Results**

Of the sixteen subjects that completed the study, 12 consumed additional kcals above their normal intake. These 12 subjects consumed 6±5 jars of peanut butter over the 4-week treatment period. Four subjects did not consume additional kcals despite consuming 4±3 jars of peanut butter over the 4-week period. Energy and fat intake increased in the 12 subjects that actually overfed (Kcals/day [p=0.0533]: Pre 2104±705 Post 2611±1342, Fat grams/day [p=0.0567]: Pre 77±33 Post 126±86). There were no significant changes in carbohydrate (grams/day: Pre 181±62 Post 185±64) or protein intake (grams/day: Pre 171±116 Post 189±144). Fat mass significantly increased [p=0.0503] (Pre 12.0±5.4 Post 12.7±4.7 kg). There were no significant pre to post changes in body weight, lean body mass or total body water.

**Conclusions**

Overfeeding on peanut butter (~500 extra kcals) results in an increase in fat mass. This outcome differs from prior studies in our lab in which subjects have overfed on protein for a much longer period of time. Despite the healthy “halo” that surrounds the consumption of peanut butter, eating too much of it will make you fatter.

## A5 A bone to pick: the myth of high-protein intake and bone harm

### Anya Ellerbroek, Cassandra Carson, Cara Axelrod, Victoria Burgess, Tobin Silver, Corey Peacock, Jose Antonio

#### Department of Health and Human Performance, Nova Southeastern University, Davie, FL, USA

##### **Correspondence:** Jose Antonio (ja839@nova.edu)

**Background**

It has been suggested that consuming a higher protein diet can be deleterious to bone health. This notion is based on the acid-ash hypothesis which claims that foods such as animal protein, milk, etc may promote a drop in blood pH; this in turn is buffered by minerals released from bones. Hence, purportedly bone demineralization occurs. The purpose of this study was to determine if consuming a high protein diet for one year had any effect on measures of whole body and lumbar bone mineral content or density.

**Materials and methods**

Twenty-two exercise-trained women participated in this study (mean±SD – age: 35±9, height cm: 167±7). Subjects recorded their food intake via MyFitnessPal ~3 times per week for one year. Subjects were instructed to consume a high protein diet for at least 6 months (>2.2 g/kg/d). The other 6 months they were instructed to consume a lower protein diet (<2.2 g/kg/d). The order was randomized. Body composition and bone health (i.e., whole body and lumbar spine) were assessed via the DXA pre, 6 months and 1 year.

**Results**

During the lower protein phase of the study, subjects consumed (mean±SD) 1557±379 kcal, 135±54 grams protein, 135±42 grams carbohydrate, and 55±11 grams of fat. Protein intake per unit body weight was 2.2 g/kg/d. During the higher protein phase, subjects consumed 1731±547 kcal, 163±81 grams protein, 142±50 grams carbohydrate, and 57±16 grams fat. Protein intake per unit body weight was 2.7 g/kg/d. There were significant differences in protein (g/kg/d) and total kcal between groups (protein p=0.0135 and kcal p=0.0487). No changes occurred over the course of the year (pre, 6 months, 1 year) for any measure of body composition (i.e., body weight, fat mass, lean mass, bone mass). Lumbar bone mineral density at baseline, 6 months and 1 year was 1.079±0.132, 1.068±0.115, and 1.079±0.119 grams/cm^2^. Whole body bone mineral density at baseline, 6 months and 1 year was 1.215±0.094, 1.209±0.103, and 1.209±0.091 grams/cm^2^.

**Conclusions**

Consuming a high protein diet for one year (range of protein intake 2.2-2.7 g/kg/d) has no effect on bone mineral content or density (whole body or lumbar spine). Furthermore, no changes occurred vis a vis fat mass or bone-free lean body mass. Thus, there is no evidence that a high protein diet has a harmful effect on bone health in exercise-trained women.

**Acknowledgements**

This study was unfunded; however, protein powder.

## A6 Training for the NFL Combine: body composition changes

### Stephanie Silva, Corey Peacock, Cassandra Carson, Anya Ellerbroek, Pete Bommarito, Tobin Silver, Jose Antonio

#### Department of Health and Human Performance, Nova Southeastern University, Davie, FL, 33024, USA

##### **Correspondence:** Jose Antonio (ja839@nova.edu)

**Background**

The NFL Scouting Combine is a weeklong showcase whereby college football players perform a battery of physical and mental tasks. NFL coaches, general managers and scouts use the Combine as a tool to assess the “performance” of the potential NFL football players. The purpose of this investigation was to determine the effects of a 6-week training preparation program on body composition in football players vying for the NFL Combine.

**Materials and methods**

Thirty-five collegiate football players (mean±SD – age: 22.4±0.9, height cm: 186.4±8.4) participated in a 6-week training preparation program including nutritional, physical, and medical support. Body composition was assessed via the Bod Pod. Total body water was assessed via the Impedimed. Pre vs Post values were analyzed via a paired samples t-test.

**Results**

There was a significant increase in lean body mass (p=0.0014) (Pre 86.1±10.1 vs Post 87.7±9.9 kg), a significant decrease in fat mass (p=0.0327) (Pre 16.8±8.6 vs Post 15.8±8.2 kg), and a significant decrease in % body fat (p=0.0194) (Pre 15.7±5.5 vs Post 14.8±5.4 percent).  There were no significant changes in body mass (Pre 102.9±16.5 vs Post 103.6±15.6) or total body water (Pre 64.5±8.1 vs Post 64.5±7.6 liters).

**Conclusions**

Our findings suggest that 6 weeks of training and nutritional support in preparation for the NFL Combine can produce significant and beneficial alterations in body composition vis a vis an increase in lean body mass with a concomitant decrease in fat mass and body fat percentage.

**Acknowledgements**

We would like to thank Bommarito Performance Systems for subject acquisition. We would also like to thank Crystal Jacques and Victoria Burgess for data support.

## A7 Probiotic supplementation in active men and women

### Tobin Silver, Cassandra Carson, Anya Ellerbroek, Cara Axelrod, Corey Peacock, Victoria Burgess, Jose Antonio

#### Department of Health and Human Performance, Nova Southeastern University, Davie, FL, 33024, USA

**Background**

There is evidence in rodents as well as obese adults that probiotic supplementation can promote a decrease in fat mass. For instance, Bifidobacterium animalis ssp. lactis 420 (B420) has been shown to decrease abdominal fat mass. Therefore, our laboratory determined the effects of probiotic supplementation on body composition in a group of active men and women in a double-blind, placebo-controlled two-arm investigation.

**Materials and methods**

Twenty subjects participated in this investigation (6 male, 14 female). All were actively participating in aerobic and/or resistance training for a period of at least one year. Subjects were randomly assigned to a group that received either a placebo (maltodextrin) or an encapsulated probiotic (one capsule) containing 5 billion Bifidobacterium BR03 and 5 billion Streptococcus thermophilus FP4 (Probiotical, Novara, Italy). Subjects consumed one capsule daily during the 6-week treatment period. Furthermore, subjects were instructed to not alter their diet or training regimen during this time. Body composition was assessed via dual-energy x-ray absorptiometry (DXA) (Hologic Horizon W, *Danbury CT USA*). Data are presented as the mean±SD. An ANOVA was used to assess differences between groups.

**Results**

The physical characteristics of the placebo and probiotic groups were as follows: Placebo – Age 25±4 years, Height 168±7 centimeters; Probiotic – Age 30±8 years, Height 166±8 centimeters. Six weeks of probiotic supplementation had no effect on body weight, lean body mass, fat mass, bone mineral content, body fat percentage or trunk fat mass (Table 1).

**Conclusion**

Six weeks of daily supplementation with a probiotic that contains 5 billion Bifidobacterium BR03 and 5 billion Streptococcus thermophilus FP4 in active men and women has no effect on body composition.

**Acknowledgements**

We would like to thank Dr. Ralf Jager and Probiotical (Novara, Italy) for the provision of the probiotic and placebo.

**Table 1 (abstract A7).** Body composition

	Placebo Pre	Placebo Post	Probiotic Pre	Probiotic Post	P value
Body Weight (kg)	69.0 ±15.8	69.2 ±15.1	66.9 ±12.0	67.5 ±12.4	0.9777
Lean Body Mass (kg)	48.8 ±10.8	48.8 ±11.3	44.4 ±9.0	45.2 ±9.3	0.6696
Fat Mass (kg)	17.5 ±7.6	17.7 ±6.3	19.9 ±6.9	19.7 ±7.1	0.8019
Trunk Fat Mass (kg)	7.1 ±4.1	7.3 ±3.9	8.9 ±3.7	8.6 ±3.8	0.9994
Bone Mineral Content (kg)	2.6 ±0.6	2.7 ±0.6	2.5 ±0.4	2.6 ±0.3	0.9453
Body Fat Percentage (%)	25.2 ±6.2	25.7 ±5.8	29.6 ±7.5	29.0 ±7.7	0.3636

Data are expressed as the mean±SD. n=10 for both groups. The placebo group had 4 males, 6 females; the probiotic group had 2 males and 8 females. There were no significant differences within or between groups. Legend: kg - kilograms

## A8 Effects of acute nitrate supplementation on repeated sprint performance in collegiate soccer players

### Gloria Velasquez, Nathan Hammon, McKenzie Moore, Jenna Bancroft

#### Department of Exercise Science & Human Performance, The University of Tampa, Tampa, FL, USA

##### **Correspondence:** Gloria Velasquez (gloriavelazquez@yahoo.com)

**Background**

Acute (1.5-3 hours pre-exercise) dietary nitrate (NO_3_) has been shown to improve blood flow to active muscle tissue under hypoxic conditions (which occurs during repeated sprints). Additionally, multiday (≥ 3days) supplementation improves mitochondrial efficiency (less O_2_ is used to produce the same amount of ATP) and decreases the ATP necessary for muscle contractions [1, 2, 3]. Collectively, these effects may lead to improvements in repeated sprint performance due to better ATP turnover, decreased PCr degradation (less Pi), and improvements in type II force production [1, 2, 3, 4, 5, 6]. Although studies have shown multiday NO_3_ supplementation improves exercise performance, minimal research exists on acute NO_3_ consumption and repeated sprint performance. The purpose of this study was to determine the effect of acute NO_3_ supplementation on repeated sprint performance in trained collegiate athletes during a field-based repeated sprint test.

**Materials and methods**

A single-blinded, randomized, crossover study was performed over a 1 week period on eight healthy collegiate male soccer players (21.1 ± 1.4 years). After participants completed a warm-up, they performed the sprint protocol consisting of 6x40m max sprints followed by 30 seconds of active recovery. An electronic timing system ensured accurate sprints times were collected. Immediately after completion of the 6 sprints, subjects took 140mL of either the placebo (pomegranate juice) or beet root juice (BRJ) shot (Beet It Sport Shots). After 2 hours passed, they performed the same warm-up and sprint protocol as used earlier.

**Results**

A trend towards significant condition effect (pre-to-post) was demonstrated for BRJ group in sprint time (p = 0.10) with an average sprint time reduction of 0.3% (-0.017s), whereas consumption of the placebo increased times by 1.96% (+0.117s), the same was true for the overall power (p = 0.06) with an average increase of 1.5% (+9.68 watts) for BRJ and a decrease by 4.29% (-26.12 watts) for the placebo. A significant trend was also found towards an interaction effect for rate of perceived exertion (RPE) during the trials (p = 0.08), suggesting that RPE decreased to a greater extent (-30.3% or -2.1 units) during the BRJ post trial compared to placebo.

**Conclusions**

This study demonstrated a trend towards a significant effect in the enhancement of repeated sprint performance when acutely supplementing NO_3_ (800mg); mostly by improving sprint times, power output, and RPE in collegiate soccer players. Although not significant, BRJ added the ability to resist fatigue by maintaining high performance levels through a repeated sprint test.

**Acknowledgements**

Dr. Eduardo Oliveira de Souza, Dr. Nauris Tamulevicius

**References**

1. Bailey, S., Fulford, J., Vanhatalo, A., Winyard, P., Blackwell, J., & DiMenna, F. et al: Dietary nitrate supplementation enhances muscle contractile efficiency during knee-extensor exercise in humans. *Journal of Applied Physiology*. 2010, **109**(1), 135-148. http://dx.doi.org/10.1152/japplphysiol.00046.2010.

2. Ferguson, S., Hirai, D., Copp, S.,Holdsworth, C., Allen, J., & Jones, A. et al. Impact of dietary nitrate supplementation via beetroot juice on exercising muscle vascular control in rats. *The Journal of Physiology*. 2012, **591**(2), 547-557.http://dx.doi.org/10.1113/jphysiol.2012.243121

3. Pawlak-Chaouch, M., Boissière, J., Gamelin, F., Cuvelier, G., Berthoin, S., & Aucouturier, J. Effect of dietary nitrate supplementation on metabolic rate during rest and exercise in human: A systematic review and a meta-analysis. *Nitric Oxide*, 2016, 53, 65-76. http://dx.doi.org/10.1016/j.niox.2016.01.001

4. Hernández, A., Schiffer, T., Ivarsson, N., Cheng, A., Bruton, J., & Lundberg, J. et al. Dietary nitrate increases tetanic [Ca ^2 +^] and contractile force in mouse fast-twitch muscle. *The Journal Of Physiology*. 2012, 590(15), 3575-3583. http://dx.doi.org/10.1113/jphysiol.2012.232777

5. Affourtit, C., Bailey, S., Jones, A., Smallwood, M., & Winyard, P. On the mechanism by which dietary nitrate improves human skeletal muscle function. Frontiers In Physiology. 2015, 6. http://dx.doi.org/10.3389/fphys.2015.00211

6. Orchard, C., Pásek, M., & Brette, F. The role of mammalian cardiac t-tubules in excitation-contraction coupling: experimental and computational approaches. *Experimental Physiology*. 2009 94(5), 509-519.

## A9 Diet induced intra hepatic fat loss; benefits beyond visceral adiposity changes

### Yftach Gepner^1^, Ilan Shelef^2^, Oded Komy^1^, Noa Cohen^1^, Dan Schwarzfuchs^3^, Nitzan Bril^1^, Michal Rein^1^, Dana Serfaty^1^, Shira Kenigsbuch^1^, Hila Zelicha^1^, Anat Yaskolka Meir^1^, Lilac Tene^1^, Avital Bilitzky^1^, Gal Tsaban^1^, Yoash Chassidim^2^, Benjamin Sarusy^3^, Uta Ceglarek^4^, Joachim Thiery^4^, Michael Stumvoll^4^, Matthias Blüher^4^, Meir Stampfer^5^, Assaf Rudich^1^, Iris Shai^1^

#### ^1^ Faculty of Health Sciences, Ben-Gurion University of the Negev, Beer-Sheva, 84102, Israel; ^2^ Soroka University Medical Center, Beer-Sheva, 84102, Israel; ^3^ Nuclear Research Center-Negev, Dimona, 86106, Israel; ^4^ Department of Medicine, University of Leipzig, Leipzig, 04103, Germany; ^5^ Channing Division of Network Medicine, Department of Medicine, Brigham and Women's Hospital and Harvard School of Public Health, Boston, MA, 02138, USA

##### **Correspondence:** Iris Shai (irish@bgu.ac.il)

**Background and aim**

It is currently unclear if reduction in intra-hepatic fat (IHF) is the main mediator of the cardiometabolic beneficial outcomes of lifestyle interventions, beyond visceral adipose tissue (VAT) loss. In the present study, we explore IHF loss and its benefits across dietary interventions, beyond VAT changes.

**Methods**

In an 18-month weight-loss trial, 278 participants with abdominal obesity/dyslipidemia were randomized to low-fat (LF) or Mediterranean/low-carbohydrate (MED/LC +28g walnuts/day) diets with/without moderate physical activity. IHF and abdominal fat-depots were measured using magnetic-resonance-imaging at baseline, after 6 (sub-study only, n=158) and 18-months.

**Results**

Of 278 participants [age=48yr; 88% men; body-mass-index=30.8kg/m2; mean IHF=10.2%, (range:0.01%-50.4%)], adherence rate was 86.3%. %IHF substantially decreased after 6 [-6.6%absolute-units (-41%relatively)] and 18-months [-4.0% absolute-units (-29% relatively); p<0.001 vs. baseline], along with moderate weight-loss (-3%). Reduction of IHF was directly associated with decreased VAT, deep-subcutaneous-adipose-tissue (deep-SAT) and superficial-SAT (r>0.48, p<0.001 for all). However, when weight-loss was adjusted, IHF loss only associated with decreased VAT (β=0.155; p=0.048). After controlling for VAT loss, decreased %IHF was independently associated with reductions of HbA1c, circulating chemerin levels, serum gamma-glutamyl-transferase (GGT) and alanine-aminotransferase (ALT), (p<0.05 for all). MED/LC diet decreased %IHF (p=0.036) and induced greater improvements in cardiometabolic risk parameters (p<0.05) more than LF diet, even after adjustment to VAT changes. In contrast, the differences between diet groups (e.g. waist circumference, triglycerides, TG/HDL ratio and cardiovascular risk score) were markedly attenuated after controlling for IHF changes.

**Conclusions**

%IHF is substantially reduced by diet-induced moderate weight loss, more effectively by MED/LC diet, independent of VAT changes. IHF loss is associated with specific improved parameters. Beneficial effects of MED/LC diet are largely mediated by decreased %IHF rather than VAT loss.

**Clinical trial registry:** ClinicalTrials.gov Identifier: NCT01530724

## A10 The effect of probiotic supplementation on markers of immune and endocrine status in Division I baseball players

### Jeremy R. Townsend, David Bender, William C. Vantrease, Philip A. Sapp, Ann M. Toy, Clint A. Woods, Kent D. Johnson

#### Exercise and Nutrition Science, Lipscomb University, Nashville, TN, 37204, USA

##### **Correspondence:** Jeremy R. Townsend (jrtownsend@lipscomb.edu)

**Background**

The human gut contains a diverse collection of microorganisms which affect host immune, hormonal, nutritional, and metabolic status. As such, probiotic supplementation is increasing in popularity among athletes to aid overall health and possibly to support adaptations to training. However, little is known as to the effects of probiotic supplementation on potential markers of overtraining and overreaching in the team sport athlete. Thus, the purpose of this investigation was to determine the effects of probiotic supplementation on markers of immune and hormonal status in collegiate male athletes following 12-weeks of offseason training.

**Methods**

Twenty-five Division I male baseball athletes (20.1±1.5y, 85.5±10.5kg, 184.7±6.3cm) participated in this double blind, placebo-controlled, randomized study. Participants were randomly assigned to a probiotic (PRO; n=13) or placebo (PL; n=12) group. Throughout their 12-week offseason training program, athletes consumed PRO (DE111®; 1 billion CFU/day) or PL supplement in conjunction with their post-workout nutrition (36g CHO, 27g PRO, 2g FAT) immediately following resistance and/or sport-specific training. On weekend or non-training days, athletes consumed their respective supplement with a normal meal. Pre- and post-training, all athletes provided resting blood and saliva samples. Circulating concentrations of testosterone, cortisol, TNF-α, IL-10, and zonulin were examined in the blood, while salivary IgA and IgM were assayed as indicators of mucosal immunity. Separate analyses of covariance (ANCOVA) were performed on all measures collected at POST. Associated values collected at PRE were used as the covariate to eliminate the possible influence of initial score variances on the outcomes.

**Results**

TNF-α concentrations were significantly (p = 0.024) lower in PRO (∆: -0.25 ± 1.10pg/mL) compared to PL (∆:+0.36pg/mL). There were no significant group differences in any other biochemical markers examined. However, a trend (p = 0.078) for lower cortisol concentrations in PRO (∆:-2.79 ± 8.10ng/mL) compared to PL (∆:+1.44 ± 4.57ng/mL) was observed. Collectively, significant increases were observed for testosterone (p = 0.045), IL-10 (p = 0.048), SIgA rate (p = 0.031), and SIgM rate (p = 0.002) following 12-weeks of offseason training across groups.

**Conclusions**

These data suggest daily probiotic supplementation may attenuate circulating markers of inflammation and catabolism following 12-weeks of offseason training in collegiate baseball players. However, additional research is needed to provide further practical insight to these findings.

**Acknowledgements**

This study was supported by Deerland Enzymes Inc.

## A11 Acute ingestion of beetroot juice reduces post exercise diastolic blood pressure in obese males

### Ana P. T. Fayh^1,2^; Agnes D. L. Bezerra^1^; Daniela A. Pacheco^1^, Daniel C. Souza^3^, Luiz F. Farias-Junior^2^, Eduardo C. Costa^2^

#### ^1^ Graduate Program in Nutrition, Federal University of Rio Grande do Norte, Natal/RN, Brazil; ^2^ Graduate Program in Physical Education, Federal University of Rio Grande do Norte, Natal/RN, Brazil.

##### **Correspondence:** Ana P. T. Fayh (apfayh@yahoo.com.br)

**Background**

Numerous clinical trials have shown beneficial cardiovascular effects and improvement of performance of inorganic nitrate supplementation (NO3^−^), especially in form of beetroot juice. Despite these reports, no study to date has evaluated whether dietary nitrate in the form of beetroot juice can enhance the reduction of blood pressure (BP) following a single session of exercise in individuals who have an increased cardiovascular risk profile, but have not yet developed hypertension, such as obese individuals. Thus, the aim of this study was to examine the effects of a dietary nitrate supplementation (beetroot juice) on the post-exercise laboratory BP in obese males.

**Material and methods**

In a randomized, controlled and cross-over trial, 14 obese male subjects (25.3 ± 4.7 years; BMI = 35.8 ± 3.3 kg / m², and ≈40% body fat) were randomly submitted to three experimental sessions: 1) beetroot juice with exercise (Beet It Sport^®^, James White Drinks Ltd., Ipswich, UK, ≈ 800 mg nitrate); 2) fruit soda juice with exercise (Kapo^®^, Del Valle, Brazil, ≈ 5 mg nitrate); and 3) water without exercise. The exercise performed was moderate aerobic in treadmill, with duration of 40 minutes and intensity of 50% of reserve heart rate after. BP was measured at three different moments of the experimental session with digital esfigmomanometer (Omrom®, Brazil): before drinking (baseline, time 0), 60 minutes after drinking and immediately before exercise/control session (time 60) and one hour after acute high moderate exercise or control session (time 165). For statistical analysis, two-way ANOVA with repeated measures (condition vs time) between the experimental sessions was used, and a statistical significance of p <0.05 was considered.

Table 1 shows the results of laboratorial BP between interventions. There was no main effect of the condition x time interaction for systolic blood pressure, even though in beetroot juice and exercise there was a reduction of 8 mmHg systolic blood pressure at time 165. There was a main effect of the condition *vs* time interaction for diastolic blood pressure. Beetroot juice and exercise reduced diastolic blood pressure by 7 mmHg at time 2 (165 minutes after ingestion of juice), while fruit soda with exercise and control session did not influence BP.

**Conclusion**

Beetroot juice supplementation significantly reduced diastolic blood pressure in young men with obesity.

**Trial registration:** http://www.ensaiosclinicos.gov.br/rg/RBR-5gkhhj/

**Table 1 (abstract A11).** Values of laboratorial blood pressure between the experimental sessions at different periods of trial

	Time 0	Time 60	Time 165	p-value		
	Mean ± SD	Mean ± SD	Mean ± SD	Condition	Time	Interaction
SBP				0.619	0.054	0.349
CON	124.8 ± 8.8	127.7 ± 10.5	123.2 ± 14.6			
BJE	130.4 ± 14.5	124.8 ± 12.0	121.7 ± 13.3			
FSE	127.0 ± 10.7	127.8 ± 13.2	125.0 ± 11.1			
DBP				0.145	0.079	0.013
CON	82.3 ± 6.1	83.0 ± 10.1	84.8 ± 9.1			
BJE	85.1 ± 8.5	76.4 ± 9.2	78.1 ± 7.7			
FSE	82.3 ± 9.0	80.9 ± 7.2	85.6 ± 10.7			

SBP: systolic blood pressure; DBP: diastolic blood pressure; CON = control without exercise, BJE = beetroot juice with, FSE = fruit soda with exercise. Values are expressed in mean ± standard deviation. P-value with two-way ANOVA with repeated measures (condition vs time).

## A12 Impact of immediate, pre-exercise ingestion of branched-chain amino acid and taurine on soreness and performance following eccentric exercise

### Ross A. Sherman^1^, Morgan E. Kennedy^1^, Rhyan J. Wozniak^1^, Dylan L. Runions^1^, and Jordan R. Moon^2^

#### ^1^Grand Valley State University, Allendale, MI, USA; ^2^ImpediMed Inc., Carlsbad, CA, USA.

##### **Correspondence:** Ross A. Sherman (ross.sherman@gvsu.edu)

**Background**

Eccentric exercise can cause structural damage and functional perturbations for several days post-exercise. Branched-chain amino acids (BCAAs) reduce muscle soreness by assisting with muscle recovery, and taurine has cyto-protective properties that reduce muscle damage following eccentric exercise. Combined BCAAs and taurine have been reported to provide complimentary benefits after multiple and consecutive days of ingestion. However, it is unclear whether a single, pre-exercise ingestion provides any functional or perceptual benefits. This study investigated the impact of a single, immediate pre-exercise ingestion of BCAAs and taurine on performance and soreness following predominantly eccentric exercise.

**Materials and methods**

40 recreationally active participants (21±4 years; 1-RM 92.8±29.0 kg) were divided into four (BCAAs and taurine [BCAA-Tau]; BCAAs only [BCAA-P]; taurine only [P-Tau]; and placebo [P-P]) supplement (Amino1^TM^, MusclePharm Inc., Burbank, CA; BCAA 6 g [3:1:2; L-Leucine 3 g, L-Valine 2 g, L-Isoleucine 1 g]; Taurine 2 g;) groups using a randomized, double-blind allocation. Preliminary testing comprised 1-RM barbell back squat, 40-yd sprint, vertical jump, T-test agility, and perceptual soreness. Participants reported to the laboratory having refrained from damaging and high-intensity exercise, and ingestion of BCAA and taurine supplements for 48 hours. After supplementation, participants completed the exercise bout (3x12 barbell back squats at 65% 1-RM, 3x12 weighted lunges, and 5x10 18” depth jumps). Further functional and perceptual soreness tests were completed 1, 4, 24, 48 and 72-h post-exercise. Null hypothesis tests and magnitude-based inferences were used to identify statistically and practically significant changes in dependent variables.

**Results**

BCAA-Tau supplementation had a “likely positive” effect on 40-yd sprint time (non-significant change; p=0.210) and t-test agility run time (non-significant change; p=0.095) at 24-h, 48-h, and 72-h post-exercise compared to placebo intake. BCAA-Tau supplementation had a non- significant (p=0.074) but “likely positive” effect on vertical jump height at 48-h and 72-h post- exercise compared to placebo use. BCAA-Tau was found to have a significant (p=0.021) and “likely negative” impact on perceived soreness at 24-h, 48-h and 72-h post-exercise compared to placebo.

**Conclusion**

A single, pre-exercise ingestion of BCAA-taurine supplement appeared to provide performance benefits between 24-h and 72-h post-exercise. The use of perceptual scales might not be appropriate when used to equate perceived soreness to functional ability. Further trials that extend either the pre-exercise or post-exercise ingestion period are also recommended.

**Acknowledgements**

This study was supported by a grant from MusclePharm and the International Society of Sports Nutrition.

## A13 Do high-protein diets affect sleep quality and quantity in exercise-trained men and women?

### Cassandra Carson^1^, Jaime Tartar^2^, Anya Ellerbroek^1^, Cara Axelrod^1^, Corey Peacock^1^, Tobin Silver^1^, Victoria Burgess^3^, Jose Antonio^1^

#### ^1^Department of Health and Human Performance, Nova Southeastern University, Davie, FL, 33024, USA; ^2^Department of Psychology and Neuroscience, Nova Southeastern University, Davie, FL, 33024, USA; ^3^College of Human Performance, Concordia University, Chicago, IL, 60305, USA

##### **Correspondence:** Jose Antonio (ja839@nova.edu)

**Background**

There is evidence to suggest that one’s diet may affect sleep quality and/or quantity. For instance, six months of modest caloric restriction in overweight individuals has been shown to shorten sleep onset latency. Furthermore, 12 weeks of a higher protein diet (1.5 g/kg/d) may improve sleep in overweight individuals versus a low protein diet (0.8 g/kg/d). Therefore, the purpose of this investigation was to determine if a high-protein diet (>2.2 g/kg/d) affected parameters of sleep in exercise-trained men and women. This is the first investigation of its kind that has examined the effects of a high-protein diet in trained men and women.

**Materials and methods**

Eighteen subjects (6 male, 12 female) participated in this 14-day randomized crossover investigation (mean±SD: age: 32±8 years; height: 162.9±29.0 centimeters; body weight: 65.6±6.0 kilograms; body fat percentage: 17.8±6.7 %). All subjects were exercised trained (mean±SD: average years of training: 14±8 years; average hours of resistance training per week: 4±2 hours; average hours of aerobic exercise per week: 4±2 hours). Subjects consumed a high-protein (>2.2 g/kg/d) and a lower protein diet (control, <2.2 g/kg/d) for 7 days in a randomized order. Total sleep time and quality was calculated through the use of Actiwatch wrist monitors and Actiware software (Phillips Respironics, New Jersey). Subjects wore the Actiwatch wrist monitors 24 hours per day for 14 consecutive days. Body composition was assessed via the Bod Pod^**®**^.

**Results**

There was a significantly higher intake of protein and calories during the high-protein phase of the study; however, there were no significant differences vis a vis the other dietary measures (Table 1). There was no effect of protein intake on any measures of sleep (Table 2).

**Conclusion**

Consuming a high-protein diet for one week had no effect on sleep quantity or quality. Furthermore, it is evident that the individuals in this study are chronically sleep-deprived.

**Table 1 (abstract A13).** Diet

	Control	High-Protein
Kcal	1740±396	2105±451*
Protein (g)	122±50	214±63*
Carbohydrate (g)	176±64	165±65
Fat (g)	61±21	66±21
Kcal/kg/day	27.3±8.2	32.7±8.3*
Protein g/kg/day	1.9±0.9	3.3±1.2*
Carbohydrate g/kg/day	2.8±1.2	2.6±1.1
Fat g/kg/day	1.0±0.4	1.0±0.3

Data are expressed as the mean±SD. Legend: g- grams, kg – kilograms

*Significant difference between control and high-protein, p<0.0001

**Table 2 (abstract A13).** Sleep

	Control	High-Protein
Sleep duration (hours)	6.70±0.65	6.47±0.76
Onset latency (minutes)	12.2±15.0	15.0±13.0
Sleep efficiency (percent)	85.1±4.1	84.5±3.5
WASO (minutes)	48.3±20.4	44.1±11.1
Number of awakenings	24.1±5.4	23.6±6.4

Data are expressed as the mean±SD. Legend: WASO – wake time after sleep onset (i.e., amount of time a person spends awake after first falling asleep). Onset latency – how long it takes to fall asleep. Sleep efficiency – percentage of time you are actually sleeping after you fall asleep. Number of awakenings – number of times you wake up after falling asleep.

## A14 Body composition assessment of NFL Combine athletes: BIA, Bod Pod, and DXA

### Victoria Burgess^1^, Cara Axelrod^2^, Cassandra Carson^2^, Anya Ellerbroek^2^, Tobin Silver^2^, Corey Peacock^2^, Pete Bommarito^2^, Jose Antonio^2^

#### ^1^College of Human Performance, Concordia University, Chicago, IL, 60305, USA; ^2^Department of Health and Human Performance, Nova Southeastern University, Davie, FL, 33024, USA

**Background**

Body composition may play an important role in college football. An increase in lean body mass coupled with a decrease in fat mass should theoretically lead to an improved outcome as it relates to sports performance. However, it is evident that performance in football is multi-factorial. Nevertheless, the purpose of this study was to evaluate different body composition measures (i.e., Bod Pod, DXA and BIA) in trained college football players who were preparing for the NFL Combine.

**Materials and methods**

College football players came to the laboratory on two occasions (baseline and 6-weeks post) for body composition assessment. Athletes participated in an intensive, six-week training program in preparation for the NFL Combine (Physical characteristics: 22.5 ±1.0 yr; 184.7 ± 8.3 cm height; 101.7 ± 15.3 kg body weight). Body composition was measured via dual-energy X-ray absorptiometry (DXA), Bod Pod and bioelectrical impedance analysis (BIA) In addition, total body water (TBW) was measured (Impedimed). An ANOVA was used to determine if differences existed between groups.

**Results**

There no significant differences in measures of lean body mass (p=0.1314), fat mass (p=0.2772) or percentage body fat (p=0.3816) between the three methods. See Table 1.

**Conclusion**

There were no significant differences between the DXA, Bod Pod or BIA vis a vis lean body mass, fat mass or body fat percentage. Thus at least for group measures, the BIA is a feasible method for assessing body composition.

**Table 1 (abstract A14).** See text for description.

	DXA	Bod Pod	BIA
Lean body mass (kg)	83.3±9.5	84.9±9.0	86.2±9.6
Fat mass (kg)	17.7±6.5	16.5±8.0	16.3±7.5
% Body Fat	17.1±3.6	15.6±5.0	15.4±5.1

Data are mean±SD. n=26

## A15 Acute aerobic exercise improves neurophysiological measures of emotion processing.

### Jaime L Tartar^1^, Sebastien Salzmann^1^, Roodelyne Pierrelus^1^, Jose Antonio^2^

#### ^1^Department of and Psychology and Neuroscience, Nova Southeastern University, Davie, FL, 33314, USA; ^2^Department of Health and Human Performance, Nova Southeastern University, Davie, FL, 33314, USA

##### **Correspondence:** Jaime L Tartar (tartar@nova.edu)

**Background**

A large and growing body of research demonstrates that aerobic exercise results in improved physical, mental, and emotional well-being and has even been shown to protect against depression and other mood disorders. Despite the established mental benefits of aerobic exercise, the neurophysiological mechanisms through which exercise can improve mood and emotion processing is currently unclear. In order to answer this uncertainty, we are testing the extent to which high intensity aerobic exercise, relative to a control condition, can alter the late positive potential (LPP) event related potential (ERP), which is a physiological marker of emotion processing in the brain.

**Materials and methods**

In randomized crossover trial, we compared physiological and self-report correlates of emotion processing after an acute aerobic exercise session compared to a control condition (n = 20, 10 males, mean age = 21, SD 2.78). Subjects ran on a treadmill at 70-80% of their estimated maximum heart rate for a 30-minute duration. We measured biomarkers associated with emotion processing and increased arousal (salivary alpha amylase and cortisol) at 4 different time-points at each session. Mood assessments included the profile of mood states and the state trait anxiety inventory. In order to examine brain changes associated with emotion processing, participants underwent EEG testing.

**Results**

We found that, relative to baseline testing, the LPP amplitude to emotionally-negative pictures is reduced after exercise (p < 0.05 at multiple electrode locations), suggesting decreased amygdala responsivity after exercise. In agreement, self-report measures also showed improved mood after exercise, relative to the control session t(19) = 2.27, p = 0.04. We also saw a significant session by time interaction for cortisol F(3,57) = 4.11, p = 0.01 and alpha amylase F(3,57) = 3.63, p = 0.02

**Conclusions**

The self-report mood measures agree with previous studies which showed a benefit of exercise on mood. New to our study, we show that the overall change in mood (TMD) is driven primarily by an increase in vigor and a decrease in depressive symptomatology. Combined, the biochemical data suggest that effects of exercise are likely related to activation of the sympathetic nervous systems, rather than cortisol (through HPA axis activation). New to our study, we show that exercise decreases the brain’s response to emotionally negative stimuli (measured by the LPP ERP response). Combined, these findings demonstrate a possible neurobiological explanation for mood improvements following exercise.

**Acknowledgements**

This work was supported through a Department of Education grant (P120A140012) awarded to JLT.

## A16 Competition-based caloric expenditure in NCAA division I women’s basketball

### Gabriel J. Sanders^1^, Brian Boos^1^, Roger O. Kollock^3^, Cory Scheadler^1^, Corey A. Peacock^2^, Jordan Horning^1^

#### ^1^Northern Kentucky University, Highland Heights, KY, 41076 USA; ^2^Nova Southeastern University, Fort Lauderdale, FL, 33314 USA; ^3^University of Tulsa, Tulsa, OK, 74104 USA

**Background**

Basketball is an intermittent, high intensity sport in which athletes expend a significant amount of energy throughout competition. Knowing the number of calories expended per quarter could enhance fuel replenishment protocols used to optimize in-game performance and post-game recovery.

**Methods**

Thirteen, (19.6 ± 1.3 years old) NCAA Division I women basketball athletes were monitored throughout 31 games. To track calories, athletes were maximally tested (VO_2max_) before the season and monitored with a wearable microsensor that included a heart rate strap (Polar Team Pro, Kempele, Finland) for each 40-minute game (10 minute quarters). To be included in the analysis, participants were required to participate in a minimum of three minutes in any given quarter and 10 minutes for the entire game. Three positions were analyzed: Guards, Forwards, and Centers. Multiple ANOVAs were used in the analyses.

**Results**

There was a main effect of group (*p* < 0.001) for total calories expended throughout games; Guards (907 ± 227) expended less energy than Forwards (1179 ± 316) and Centers (1200 ± 234). Guards consistently expended less energy than Forwards and Centers throughout each quarter. There was a main effect of condition (*p* < 0.001), as more energy was expended in warm-ups than all other quarters.

**Conclusions**

Energy expenditure in games is different for each position. Athletes expend more energy in warm-ups when compared to each quarter. Fuel replenishment or carbohydrate containing hydration protocols should begin after warm-ups and be specific to each position and athlete to optimize in-game performance and to enhance post-game recovery.

**Table 1 (abstract A16).** Calories expended throughout each quarter

	Pre-Game Warm-up	Max	1st Quarter	Max	2nd Quarter	Max	3rd Quarter	Max	4th Quarter	Max
Guards	375 ± 89	524	168 ± 39	266	208 ± 53	344	222 ± 53	346	260 ± 84	451
Forwards	399 ± 130	654	228 ± 57^#^	321	265 ± 73^#^	376	267 ± 70^#^	395	328 ± 96^#^	524
Centers	472 ± 132^#!^	686	214 ± 44^#^	304	266 ±47^#^	370	263 ± 53^#^	379	308 ± 84^#^	498
Team	418 ± 127^*^	686	210 ± 54^%^	321	249 ± 65^&^	376	254 ± 64^+^	395	304 ± 93^*^	524

Data are Means ±SD for calories expended^*^Different than quarters, (*p* < 0.001); ^#^Different than Guards, (*p* ≤ 0.014); ^!^Different than Forwards, (*p* ≤ 0.001); ^%^Different than 3^rd^ and 4^th^ quarters, (*p* < 0.001); ^&^Different than 4^th^ quarter, (*p* < 0.001); ^+^Different than 1^st^ and 4^th^ quarters, (*p* ≤ 0.027).

## A17 The kinetics of muscle carnosine increase with β-alanine supplementation

### Roger C Harris^1^, Dmitry Spelnikov^2^

#### ^1^Junipa Ltd., Newmarket, Suffolk, United Kingdom; ^2^Kurchatov Institute, B.P.KONSTANTINOV, St Petersburg Nuclear Physics Institute, Gatchina, Russia

##### **Correspondence:** Roger C Harris (junipa@ymail.com)

Carnosine (Carn: β-alanyl-L-histidine) is the only member of the histidine-containing-dipeptide family found in human muscle. Combined with β-alanine (β-A), the histidine residue of Carn is prevented from participating in protein synthesis enabling high concentrations to be accumulated. In addition, the pKa of the imidazole ring is raised from 6.1 to 6.83 making Carn a highly effective H^+^ buffer with a power of 0.33 slykes • mol^-1^ Carn, over the pH range: 7.1 (resting pH) to 6.5 (post-exercise).

Synthesis of Carn occurs *in situ* and is limited by β-A which has led to the use of supplements. But what dose and for how long should β-A be taken? From a review of three published studies, it was concluded that the rate of synthesis of Carn with β-A supplementation (*vform*) could be described by zero order kinetics, with a rate constant, *k*_*f*_, linearly related to dose.

$$ vform= kf\kern1em \left(\mathrm{mmol}\cdot {\mathrm{kg}}^{-1}\cdot {\mathrm{d}}^{-1}\right) $$

Further, opposing this is an on-going process of Carn decay at a rate of *vdec* (d^-1^) most probably driven by the spontaneous reaction of Carn with carbonyl groups to form adducts which are then exported from muscle. Carn decay back to the pre-supplementation level (PSL) appears first order where:

$$ vdec=-{k}_d\cdot \left[\varDelta \mathrm{Carn}\right]\kern1.5em \left(\mathrm{where}\kern0.17em \Delta \mathrm{Carn}\kern0.17em \mathrm{is}\ \mathrm{the}\ \mathrm{increase}\ \mathrm{above}\ \mathrm{PSL}\right) $$

It follows:

$$ \mathrm{d}\left[\mathrm{Carn}\right]/\mathrm{dt}= vform- vdec={k}_f- kd\cdot \left[\varDelta \mathrm{Carn}\right] $$

and by integration over time:

$$ \left[\varDelta \mathrm{Carn}\right]=\left({k}_f/{k}_d\right)\cdot \left(1-\exp \left(-{k}_d\cdot \mathrm{t}\right)\right) $$

90% effect of β-A supplementation is predicted after 200+ days and the greatest ΔCarn with the highest dose (up to 6.4g • d^-1^). The model has been used to compare the effectiveness of different β-A formulations on increasing muscle Carn with time; simulating the effects of moving from a vegetarian diet to one containing meat; in accounting for the lower level of Carn in type I muscle fibres; and estimating endogenous β-A synthesis. A study of the effects of increased activity/sedation, diabetes, COPD and sarcopenia on *k*_*d*_ would help illuminate the role of Carn, as a carbonyl group target in health and disease, in suppressing the formation of advanced glycation end (AGE) products.

*Since retiring in 2009, R Harris has acted as a consultant to NAI, Carlsbad, Ca, USA.*

**Fig 1 (abstract A17).**
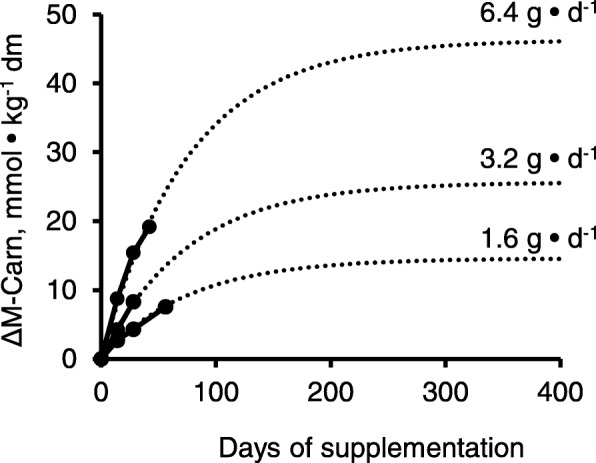
Comparison of the change in muscle Carn with time predicted by the model for subjects supplemented with 1.6 to 6.4 g • d^-1^ β-A (SR CarnoSyn®), with experimental findings from three published studies with k_d_ = 0.0133 • d^-1^ and k_f_’s of 0.1942 ± 0.0260, 0.3411 ± 0.0252, 0.6161 ± 0.0481 mmol • kg^-1^ • d^-1^ for 1.6, 3.2 and 6.4 g • d^-1^, respectively.

## A18 Comparative micronutrient adequacy of elite Australian surf lifesavers and non-athlete age- matched young adults

### Gemma Zanardo^1^, Deb Agnew^2^, Jose Antonio^3^, Kathryn Jackson^1^

#### ^1^College of Nursing & Health Sciences; Nutrition & Dietetics, Flinders University, Adelaide, SA, 5042, Australia; ^2^College of Education, Psychology & Social Work; Physical Education, Flinders University, Adelaide, SA, 5042, Australia; ^3^College of Health Care Sciences; Exercise & Sport Science, Nova Southeastern University, Fort Lauderdale, FL, 33314, USA

##### **Correspondence:** Kathryn Jackson (kathryn.jackson@flinders.edu.au)

**Background**

This observational study investigated dietary micronutrient intakes among Australian Surf-lifesaver athletes (Lifeguards). These athletes regularly compete in elite, high intensity surf sports throughout Summer. In order to achieve and maintain power-to-weight ratios conducive to strength and speed, many competitors restrict their dietary intakes, increasing their risk of micronutrient deficiencies. The sequelae of micronutrient deficiencies include poor sports performance and increased risk of low bone mineral density (BMD) and stress fractures. Supplement intakes were also investigated, to determine whether nutritional adequacy was met through supplements if dietary intakes were inadequate.

**Materials and methods**

Dietary and supplement intakes were compared for 9 young adult elite surf-lifesaving competitors (5 males, mean age 21.6±3.8 yrs; 4 females, mean age 20.5±1.7 yrs) with eleven age-matched non- athletes (4 males, mean age 20.5±1.0 yrs; 7 females, mean age 21.9±1.1 yrs). The athletes formed a homogeneous group, with identical training and competition requirements. Dietary adequacy was assessed from 4-day food intake records, using FoodWorks dietary analysis software. Supplement intakes were recorded concurrently with food intakes, using a validated questionnaire. All data were analysed using IBM SPSS Statistics for Windows, Version 22. Within and between group gender differences in dietary intakes were determined using a two-way between factor ANOVA, with post-hoc Bonferroni-correction applied for multiple tests. Bootstrapping was used to correct for small sample size. The alpha level was set at 0.05.

**Results**

Mean dietary iron and calcium intakes among male athletes and non-athletes met the Recommended Dietary Intakes (RDI) from food alone, although this result did not confer a statistically significant difference between all groups, even when supplement intakes were added. Neither female athletes nor non-athletes met the RDI for iron or calcium from dietary sources, but supplement intakes increased iron intakes for female athletes to recommended levels. However the addition of calcium supplements did not increase intakes to RDI levels among female athletes or both non-athlete gender groups and statistical analyses did not show a significant difference between any groups. Contrary to expectations, female athletes showed a significantly higher (p=0.001) mean total energy intake compared to female non-athletes, although no significant differences were observed between male athletes and non-athletes.

**Conclusion**

Insufficient dietary iron and calcium intakes among young adult female Lifeguards place them at risk of growth and performance decrements, low BMD and stress fractures. Male and female non- athletes are similarly at risk, suggesting dietary micronutrient inadequacies may be age-related rather than competitive sport-related. Hence nutrition education is urgently needed among this at risk age-group, regardless of sports participation.

## A19 Safety, tolerability and nutrient status after consuming a total meal replacement beverage for 30 days: a randomized, placebo-controlled pilot study in healthy adults

### Blake Ebersole (blake@npscientific.com)

#### NaturPro Scientific, LLC, Carmel, IN, 46032, USA

**Background**

Poor nutrition is taking an extraordinary toll on human health and productivity. “Nutrition deserts” may be solved with nutrient-dense, cost-effective and shelf-stable meal replacements. While many studies have evaluated meal replacement diets for weight loss or medical needs, few have evaluated the effects of complete meal replacements in non-obese, healthy adults in a real-world setting [1]. The primary objective of this pilot study was to evaluate the safety, tolerability and changes in nutritional status in 15 healthy adults instructed to consume only a liquid total meal replacement beverage (TMB) for 30 days.

**Methods**

30 non-obese adults (20 female, 10 male) aged 18-40 years with BMI 22-30 kg/m² in the United Kingdom were randomly assigned to the TMB (Soylent 2.0, Rosa Labs, California) (n=15) ad libitum, or the control group (n=15) who continued their normal lifestyle (Figure 1). Subjects were accustomed to the typical Western diet, had a stable body weight and consumed junk food at least once a week. Treatment was the replacement of all meals and caloric beverages with TMB ad libitum, with a target of 2,000 kcal/day (5 bottles, 400 calories each). Subjects were instructed not to change level of physical activity. Measurements at baseline, 15 and 30 days included body weight, blood count, metabolic profile, blood lipids, liver enzymes, urinalysis, electrolytes and nutrient status. All subjects completed a daily food diary and subjective scales for gastrointestinal discomfort, satiety and mood.

**Results**

29 subjects completed the study. Subjects consuming TMB experienced a modest reduction in body weight compared to the control group (P<0.001), and reduced fasting blood sugar (P<0.05) from baseline. Heavier subjects tended to consume more TMB and lose more weight than lighter subjects (Figure 2). An increase in Vitamin B12 and folate status (P</=0.001) was observed in the TMB group versus control. No other changes in safety-related or nutrient markers were found. Subjects consuming TMB rated high on satiety ratings. Subjective scores for abdominal discomfort and satiety were similar in both groups, with a small percent of subjects reporting transient changes in stomach rumbling. Consistent with previous pilot studies, TMB was safe and well tolerated [2].

**Conclusion**

This ‘stress test’ human pilot study found consumption of a target of 2,000 kcal TMB for 30 days was safe and well tolerated. TMB maintained or improved body weight and nutrient status and metabolism in this small group of healthy, non-obese subjects.

**Acknowledgments**

This study was independently conducted by Leatherhead Food Research, United Kingdom, with approval under REIC reference 080915-3. Rosa Foods, Los Angeles, California sponsored the study, provided financial support and study materials.

NaturPro Scientific LLC conducted an independent scientific review of the study data, and declares no interest or stake in any company or individual involved with the study.

**References**

1. Noakes M, Foster PR, Keogh JB, Clifton PM. Meal replacements are as effective as structured weight-loss diets for treating obesity in adults with features of metabolic syndrome. J Nutr. 2004 Aug;134(8):1894-9.

2. Hsu RH, McCormick DM, Seitz MJ, Lui LM, Rishi HS, Arkin AP. An interventional Soylent diet increases the *Bacteroidetes* to *Firmicutes* ratio in human gut microbiome communities: a randomized controlled trial. bioRxiv 2017; doi: 10.1101/200881

**Fig. 1 (abstract A19).**
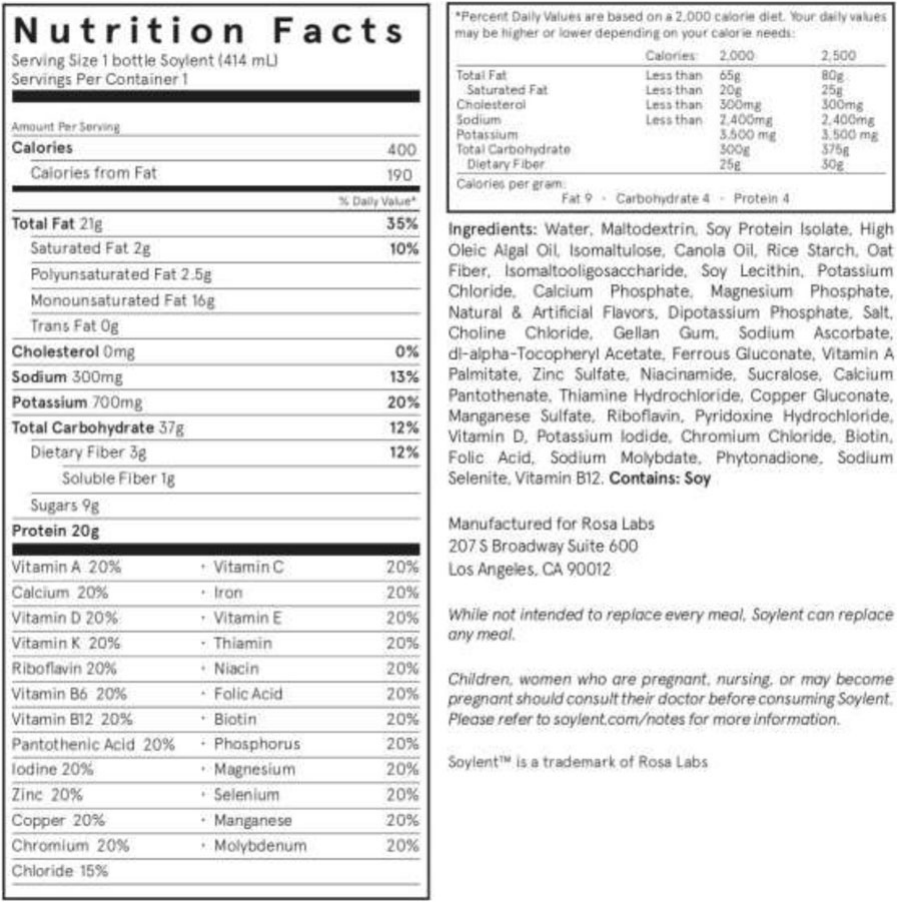
Nutritional Content of TMB. The total meal replacement beverage (TMB) when consumed as instructed in the study (5x daily) contained 100g protein, 105 grams of fat and 185 grams carbohydrates, including 45 grams of sugar, and 100% daily value of essential nutrients per day

**Fig. 2 (abstract A19).**
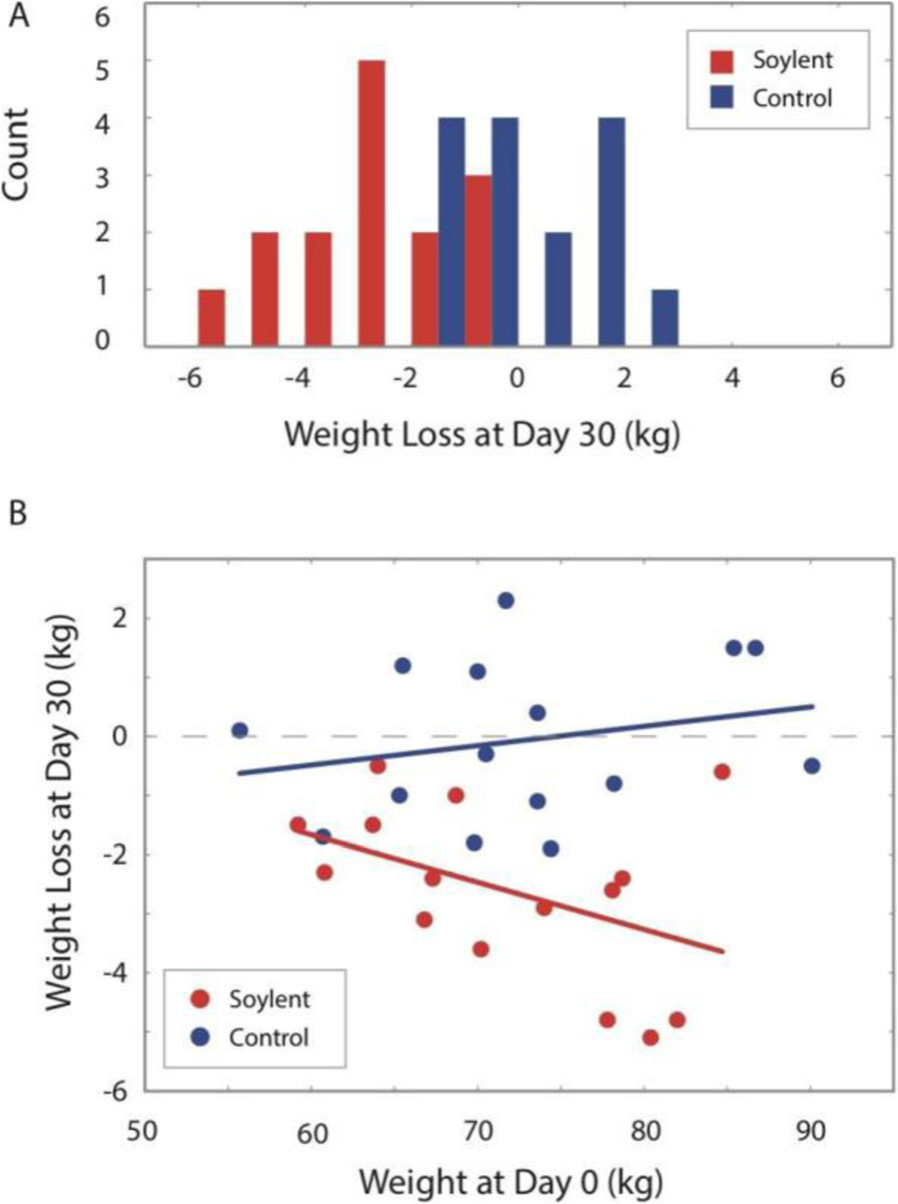
Ad libitum consumption of 2,000 kcal/day TMB trended toward greater weight loss in heavier subjects

## A20 Applying an Xbox Kinect to the two-compartment model of body composition

### Greg Popovich^1^, Kristy Henson^2^, Rachel Fulks^1^, Tara Goldman^1^, Alea Hess^1^

#### ^1^School of Exercise Science & Athletic Training, West Virginia Wesleyan College, Buckhannon, WV, 26201, USA; ^2^Department of Biology & Environmental Science, West Virginia Wesleyan College, Buckhannon, WV, 26201, USA

##### **Correspondence:** Greg Popovich (Popovich.g@wvwc.edu )

**Background**

Body composition is an important determinant of health status. Current two-compartment methods used to determine human body composition are hydrodensitometry, air displacement plethysmography (BodPod), and dual-energy X-ray absorptiometry (DXA). These options are expensive, inconvenient, lack portability, and are simply unavailable to many people. Conversely, field methods for body composition analysis are portable but often have questionable validity. The purpose of this study is to apply the new technology of three-dimensional (3D) scanning to the two-compartment model of body composition by capturing volume. We tested the reliability and validity of an Xbox Kinect V1 motion-capturing input device. This technology is relatively inexpensive and is already present in many homes where it is used for gaming. To our knowledge, this is the first study to attempt to use affordable and accessible scanning technology to determine body fat percent (BF%).

**Materials and methods**

We applied the Xbox Kinect V1 to the two-compartment model, which relies on the subject’s density and assumes the body consists of fat-free mass and fat mass. Participants (n=32) wore form-fitting clothing with hair placed on top of the head. Participants stood upright with hands at sides and feet tight together. A complete 360-degree 3D model of the subject was captured. All scans underwent post processing, removing background artifacts. Scans were exported as stereolithography (STL) files and transferred to Blender to calculate model volume. To calculate density, subjects’ mass was divided by volume minus residual lung volume (RLV). The Siri or Schutte equations were used to convert body density to BF%. Skinfold caliper BF% estimates were compared to the 3D scanning results.

**Results**

Interobserver error was tested using ANOVA (p=0.2), with no statistically significant differences between operators. Subjects were successfully captured as 3D images, although regression results (R^2^=0.04, variance=1008) indicate that XBox Kinect consistently yielded a higher BF% than indicated by skinfold calipers (average percent error = 68.79%). Xbox Kinect and accompanying software provided erroneously high volume estimates, which decreased density while increasing BF%.

**Conclusion**

The Xbox Kinect V1 suffers from common 3D scanner limitations (e.g., difficulty capturing reflective surfaces, sharp angles, and holes). These limitations are corrected with a skilled operator. Our research indicates the need for an equation to correct this over-estimated BF%, or additional post-processing such as a calculated polygonal simplification. Future research using the updated Kinect V2 is warranted, while comparing 3D models to criterion measures such as hydrostatic weighing or BodPod.

## A21 Vitamin D status and its relationship to body composition in a diverse sample of collegiate women athletes

### Jenna M. Worswick^1,3^, Jennifer B. Fields^1,3^, Sina Gallo^2,3^, Deanna Busteed^3^, Margaret T. Jones^1,3^

#### ^1^Health and Human Performance, George Mason University, Manassas, VA, USA; ^2^Nutrition and Food Studies, George Mason University, Fairfax, VA, USA; ^3^Center for Sports Performance, George Mason University, Fairfax, VA, USA

##### **Correspondence:** Margaret T. Jones (mjones15@gmu.edu)

**Background**

Vitamin D is important for bone health [1,2] and is recognized to play a role in autoimmune disease, respiratory infections, and cancer [1]. Among athletes, it has been found to be associated with musculoskeletal function, injury prevention, and sports performance [1]. Women athletes are at higher risk for bone diseases [3] yet, the relationship between vitamin D status and body composition among this group is understudied. In addition, women with darker skin color may be at higher risk for vitamin D deficiency [1,2]. Therefore, the purpose was to examine relationships between vitamin D status, body composition, and skin pigmentation in a sample of National Collegiate Athletic Association Division I (NCAA-DI) women athletes.

**Materials and methods**

NCAA-DI women athletes from volleyball (VB; n=12), basketball (BB; n=12), and track and field (TF; n=12) were tested January - March 2017, the non-vitamin D synthesizing period in northern Virginia. Measures included: serum 25-hydroxyvitamin D ((25(OH)D)) using enzyme-linked immunosorbent assay (ELISA); body composition, including bone mineral density, using dual energy x-ray absorptiometry (DXA); and skin pigmentation via a spectrophotometer. In addition, participants self reported their daily intake of dietary vitamin D (food frequency questionnaire) and total daily sun exposure. One-way analysis of variance (ANOVA) analyzed mean differences in all measures across sports category. Linear regression examined the relationship between 25(OH)D, BMD, LBM, and skin pigmentation.

**Results**

Participants (mean ± SD; age, 19.4±1.4 y; 172.75±8.21 cm; 70.9±13.2 kg; 22.9±4.1% body fat) ranged in skin pigmentation (13% very fair, 33% fair, 5% medium, 11% olive, 36% dark). Overall mean 25(OH)D was 28.2 ± 12.9 ng/ml, 88% of athletes were considered insufficient (≤40 ng/ml). No differences in serum 25(OH)D occurred across sport, despite BB athletes consuming significantly higher (p=0.007) dietary vitamin D (760.9 ± 484.2 IU/d) compared to VB (342.6 ± 257.8) and TF (402.3 ± 376.4). Further, LBM significantly (p=0.001) negatively predicted 25(OH)D levels ((β= -0.752; 95% CI: (-128.157 – (-22.325)), (p=0.001).

**Conclusions**

Vitamin D insufficiency was prevalent among this sample and inversely related to LBM. The lack of relationships between 25(OH)D and BMD, as well as skin pigmentation do not support previous findings in healthy adults [2]; therefore, caution should be exercised in applying results to NCAA-DI women athletes. Further, the inverse association between 25(OH)D and LBM is of interest and warrants further investigation. Routine monitoring of vitamin D status and appropriate dietary sources and/or supplementation are recommended to sustain athlete health and sport performance.

**References**

1. Ogan D, Pritchett K: Vitamin D and the Athlete: Risks, Recommendations, and Benefits. *Nutrients*. 2013;5(6):1856-1868.

2. Mitchell DM, Henao MP, Finkelstein JS, Burnett-Bowie S-AM: Prevalence and predictors of vitamin D deficiency in healthy adults. *Endocr Pract*. 2012;18(6):914-923.

3. Mudd LM, Fornetti W, Pivarnik JM. Bone Mineral Density in Collegiate Female Athletes: Comparisons Among Sports. *J Athl Train*. 2007;42(3):403-408.

## A22 Accuracy of DXA and impedance-based devices for body composition assessment in male and female bodybuilders

### Grant M. Tinsley, Austin J. Graybeal, M. Lane Moore, Megan R. Cruz, Alfred K. Kankam Jr., Michael I. Villarreal

#### Department of Kinesiology & Sport Management, Texas Tech University, Lubbock, TX, 79424, USA

##### **Correspondence:** Grant M. Tinsley (grant.tinsley@ttu.edu)

**Background**

To appropriately evaluate the effectiveness of lifestyle modifications undertaken to improve body composition, valid assessment methods are needed. The purpose of this study was to examine the group and individual accuracy of body composition estimates obtained from multi-compartment models, dual-energy x-ray absorptiometry (DXA), and several impedance-based devices in male and female bodybuilders.

**Methods**

Twenty-seven male (n=17; BMI: 29.4 ± 2.2 kg/m^2^; body fat: 11.8 ± 4.4%) and female (n=10; BMI: 23.3 ± 1.7 kg/m^2^; body fat: 19.7 ± 4.9%) bodybuilders underwent duplicate assessments via DXA, bioimpedance spectroscopy (BIS), electrical impedance myography (EIM), and three bioelectrical impedance analysis (BIA) devices. In addition to utilizing standard output, multi-compartment models were generated. For each method, body fat %, fat-free mass and fat mass were compared to the reference 4-compartment model for the evaluation of group and individual errors. Paired-samples t-tests with a Bonferroni-corrected alpha level were performed, and metrics of group and individual accuracy (e.g. constant error [CE], total error [TE], SEE, and 95% limits of agreement [LOA]) were calculated for each method.

**Results**

The 3-compartment model with a BIS body water estimate produced the lowest SEE, TE, and LOA for all variables, although some alternative methods had lower CE. In general, multi-compartment models with BIS or multi-frequency BIA (MFBIA) body water estimates produced more accurate body composition estimates than single assessment techniques (i.e. DXA, BIS, EIM and BIA). Some single assessment techniques produced low group errors. However, all single assessment techniques produced LOA large enough (e.g. ± 4 – 9% fat) to make the accuracy of these methods questionable when assessing individual athletes.

**Conclusions**

Multi-compartment models are recommended for improved group and individual accuracy in muscular athletes. Three-compartment models can exhibit excellent accuracy when compared to a reference 4-compartment model. Therefore, when DXA is unavailable for use in a 4-compartment model, 3-compartment models can be utilized by obtaining estimates of body volume (via hydrostatic weighing or air displacement plethysmography) and body water (via BIS or MFBIA). When multi-compartment models are not feasible, groups of muscular athletes may be assessed using single assessment techniques, such as DXA and BIS, with fairly good to very good accuracy. However, appropriate caution should be employed when interpreting and utilizing body composition estimates, particularly from single assessment techniques used in individual athletes.

**Acknowledgements**

The authors have no conflicts of interest to report.

## A23 Positive impact of specific bioactive collagen peptide intake on ligaments and tendons

### Steffen Oesser, Michael Schunck

#### Collagen Research Institute, Kiel, 24103, Germany

##### **Correspondence:** Steffen Oesser (Steffen.Oesser@cri-mail.org)

**Background**

A very recent clinical trial demonstrated that the intake of specific collagen peptides (TENDOFORTE^®^), has a positive impact on subjects suffering from chronic ankle instability (CAI)[^1^]. After a three month supplementation of TENDOFORTE^®^, ankle stability clearly improved compared to placebo, as indicated by the Cumberland Ankle Instability Tool and the Foot and Ankle Ability Measure (p < 0.01). In addition, a three-month follow-up revealed a significant decline in the number of ankle joint injuries (p < 0.05). The reason for the positive clinical results observed was unclear. Therefore, the effect of TENDOFORTE^®^ was investigated in pre-clinical tests on human ligament and tendon cells to elucidate the direct impact of specific collagen peptide supplementation on the extracellular matrix molecule (ECM) synthesis.

**Materials and methods**

Primary fibroblasts derived from human anterior cruciate ligaments and Achilles tendons were isolated by enzymatic digestion and seeded in monolayer cultures. After 80% cell confluence, the regular culture medium was supplemented with specific bioactive collagen peptides (TENDOFORTE^®^, GELITA AG, Germany). The RNA expression of the extracellular matrix molecules (ECM), type I collagen, proteoglycans and – in the case of ligament cells - elastin was determined via real-time PCR after 24 hours of culture. Furthermore, the ECM biosynthesis of tendon and ligament derived fibroblasts was determined using validated methods like western blotting, Alcian blue staining, or ^14^[C]-incorporation assay. Statistically significant differences in the RNA expression and ECM biosynthesis were tested in comparison to untreated control experiments with the One-Student’s *t*-test.

**Results**

The data revealed that supplementation with specific collagen peptides led to a statistically significant increase (p < 0.05) in the RNA expression of the tested ECM molecules (type I collagen, proteoglycans, elastin) in ligament cells compared with placebo. These data were confirmed on protein level with a 20 to 50% (p < 0.05) increase in the biosynthesis of the matrix molecules after TENDOFORTE^®^ treatment. Comparable results could be observed after supplementation in tendon cells. On RNA level, a statistically significant (p < 0.05) increase in type I collagen and proteoglycan expression was determined compared to the untreated controls. Moreover, the biosynthesis of collagen and proteoglycans increased significantly with TENDOFORTE^®^ treatment.

**Conclusions**

The results clearly show the stimulatory impact of specific collagen peptides on ECM molecules in ligament and tendons. The increased biosynthesis of the predominant matrix molecules in both tissues might explain the positive results on ankle stability and the reduced injury rate observed in the clinical trial.

**Acknowledgements**

none

**References**

1. Dressler D, Oesser S, Zdzieblik D, Gehring D, Gollhofer A, König D. Improvement of functional ankle properties in subjects with chronic ankle instability following supplementation with specific collagen peptides. J.Sport Science Med. 2018; accepted.

## A24 A comparative pharmacokinetic evaluation of caffeine in two different delivery vehicles in healthy adults

### Douglas Kalman^1,2^, Susan Hewlings^2,3^, Robin Lee^1^, Richard Foster^1^ and Kayce Morton^1^

#### ^1^QPS – Biokinetics, Springfield, MO, 65619, USA; ^2^Substantiation Sciences, Inc., Weston, FL, 33326, USA; ^3^Central Michigan University, Mt. Pleasant, MI, 48804 USA

##### **Correspondence:** Douglas Kalman (douglas.kalman@qps.com)

**Background**

The purpose of this prospective pharmacokinetic (PK) study was to evaluate two different delivery systems for caffeine in healthy adults. Caffeine is found in foods, beverages and in dietary supplements. Athletes often utilize caffeine as an ergogenic aid for sport and mental performance. The timing of caffeine ingestion for ergogenic use has typically been centered on timing for events that commence within an hour of dosing, however for events of longer duration, caffeine dosing may not have the desired impact. Sustained or delayed release caffeine may have ergogenic use for athletes who engage in prolonged training or who want to customize their caffeine experience.

**Materials and methods**

In a prospective randomized two-way counterbalanced cross over pharmacokinetic trial, 12 healthy adults (35.8±11.62 y.o., BMI of 26.67±4.63 kg/m^2^, 7 males, 5 females) under standardized conditions were given either Immediate Release Caffeine 250mg (IR) or Extended Release Caffeine 250 mg (ER; zümXR® , Nano Pharmaceuticals, Denver, CO.) with the opposite administered after a three-day washout. Subjects were dosed and plasma caffeine was measured over a 12 hr post-dose period. Plasma caffeine was measured by LC MS/MS (Keystone Bioanalytical). The PK profile for Tmax, Cmax and terminal ½ life was determined by linear mixed effects model. Standard statistical techniques were also used.

**Results**

Dissolution tests of the raw material revealed that the IR caffeine delivers 100% of the caffeine within1 hour. The ER caffeine (ZümXR^®^) was found to deliver 26% caffeine by 1 hour and 79% by 6 hours. The Per Protocol analysis (n=12) revealed that the IR caffeine had a Cmax of 7.42 ± 1.78 ug/ml, Tmax of 1.0 hr and a half-life (t ½ h) of 6.38 ± 2.71 hr. In contrast, the ER caffeine (zümXR®) had a Cmax of 3.66 ± 1.13 ug/ml, a Tmax of 4.0 hr and a half-life (T ½ h) of 10.5± 5.12 hr.

**Conclusions**

This study demonstrated that the ER caffeine has a different and extended PK profile relative to the IR caffeine. The ER caffeine exhibited a 300% longer Tmax and 64.6% longer half-life than the IR caffeine. This human data supports the dissolution data, adding strength to the overall findings. ER caffeine such as zümXR® technology may have application for athletes who desire extended circulating levels of caffeine.

**Acknowledgements**

This study was sponsored by Nano Pharmaceuticals (Denver, CO). All intellectual property rights reside with the owner.

## A25 The effects of Shilajit supplementation on fatigue-induced decreases in muscular strength

### Joshua L. Keller, Terry J. Housh, Ethan C. Hill, Cory M. Smith, Richard J. Schmidt, Glen O. Johnson

#### University of Nebraska – Lincoln, Nebraska, 68588, USA

##### **Correspondence:** Joshua L. Keller (joshuakeller10@gmail.com)

**Background**

The use of nutritional supplements to improve human performance has garnered substantial interest. Specifically, Shilajit is composed of fulvic acid, dibenzo-α-pyrones, proteins, and minerals. The purpose of the study was to examine the effects of 8-wks of Shilajit supplementation at 250 mg·day^-1^ (low dose) and 500 mg·day^-1^ (high dose) versus placebo on fatigue-induced percent decline in maximal voluntary isometric contraction (MVIC).

**Materials and methods**

Thirty recreationally-active men ($\overline{X}$ ± SD; age: 21.6 ± 2.5 years, height: 180.8 ± 6.0 cm, weight: 85.7 ± 15.4 kg) participated in the investigation. The subjects were randomly assigned to the high dose, low dose, or placebo group (each group: n=10). The pre-supplementation testing, the subjects performed 2, pretest MVICs followed by 2 sets of 50 maximal, bilateral, concentric isokinetic leg extensions at 180°·s^-1^ separated by 2-min of rest, and then 2, posttest MVICs. Percent decline in MVIC was defined as: $\frac{Pretest\ MVIC- Posttest\ MVIC}{Pretest\ MVIC}\ast 100.$The subjects supplemented with either 250 mg·day^-1^, 500 mg·day^-1^, or placebo for 8-wks and then returned for post-supplementation testing and repeated the pre-supplementation testing procedures. An analysis of covariance (ANCOVA) was used to examine post-supplementation differences between groups for the adjusted mean percent decline in MVIC values covaried for pre-supplementation values.

**Results**

At the pre-supplementation testing visit, there was a significant (p=0.03; ${\eta}_p^2$=0.17) difference in percent decline values between the groups. The ANCOVA indicated that there was a difference in the adjusted post-supplement values (p=0.04; ${\eta}_p^2$=0.17). The post-hoc pairwise comparisons indicated that the percent decline in MVIC for the high dose group was significantly less than both the low dose (8.9% vs. 17.0%; p = 0.002; Cohen’s D (*d)=* 1.1) and placebo (8.9% vs. 16.0%; p = 0.044; *d*= 0.95) groups. There was no difference in percent decline in MVIC between the low dose and the placebo groups (17.0% vs. 16.0%; p=0.774; *d*= 0.13).

**Conclusions**

In conclusion, the high dose group maintained more muscular strength than the low dose and placebo groups. Thus, the findings indicated that a daily dose of 500 mg was beneficial for reducing the effect of fatigue on the expression of maximal strength.

**Acknowledgements**

This project was funded by Natreon Inc., and we would like to thank all the subjects for their participation.

## A26 Can exercise intensity and timing modify post-exercise energy intake? A preliminary study

### Valéria Leme Gonçalves Panissa^1^, Ursula Ferreira Julio^1^, Alícia Tavares Gomes^1^, David H. Fukuda^2^, Monica Yuri Takito^1^, Emerson Franchini^1^

#### ^1^School of Physical Education and Sport, University of São Paulo, São Paulo, 08940-000, Brazil; ^2^Institute of Exercise Physiology and Wellness, Sport and Exercise Science, University of Central Florida, Orlando, 32789, Florida

##### **Correspondence:** Valéria Leme Gonçalves Panissa (valeriapanissa@gmail.com)

**Background**

Various strategies to avoid post-exercise energy intake (EI) compensation have recently been investigated. Manipulating exercise intensity has shown more pronounced effects on hunger suppression, greater reduction on orexigenic, and greater increase in anorexigenic hormones; however, decreased EI after high-intensity compared to moderate-intensity exercise has not been universally reported. Furthermore, decrements in EI following exercise may differ depending on the timing of meal/snack consumption. The aim of present study was to analyze post-exercise EI considering different exercise intensities and timing relative to meal consumption.

**Materials and methods**

Ten sedentary, overweight men (age 31±3 years, peak oxygen consumption 32.3±6.7 mL.kg^-1^.min^-1^, body mass 90.8±8.8 kg, height 178.0±9.0 cm; body mass index 28.7±2.0 kg.m^-2^) completed 6 sessions. The first session consisted of anthropometric measurements and an incremental test on a cycle ergometer to determine maximal aerobic power (MAP). Each experimental protocol started upon the arrival of the participant at approximately 8 a.m. following a minimum 10-hour fast. During the five experimental sessions, participants were randomly submitted to conditions where exercise and ingestion were manipulated: 30 minutes of steady-state exercise (SSE – 50% of MAP) and high-intensity intermittent exercise (HIIE – 30s repetitions at MAP separated by 30s of passive recovery) matched for total work were performed 1-hour (SSE;HIIE) or 2.5h (SSE_delay_;HIIE_delay_) after receiving a standardized breakfast, as well as a control session in which participants did not perform any exercise. Finally, an *ad libitum* buffet was offered 3.5 hours following the completion of the standardized breakfast.

**Results**

To compare EI and macronutrient intake in different conditions, a mixed linear model was conducted, while energy expenditure between intensities was compared with a paired t-test. Energy expenditure was greater following the high-intensity (290.5±33.2 Kcal) compared to the moderate-intensity (251.7±28.3 Kcal; p < 0.001) conditions. Absolute EI was lesser in HIIE_delay_ (756.7± 282.4 kcal) than during the control session (1045.4± 353.9 kcal; p=0.014). Relative EI (absolute EI minus energy expenditure from exercise) was greater in control compared to other conditions (SSE_delay_ 592.7±315.4 Kcal; p = 0.010; HIIT 627.3±35.4 Kcal; p = 0.006; HIIT_delay_ 466.3±269.7 kcal; p < 0.001) with the exception of SSE (691.3±394.9 kcal; p = 0.103). There was no effect of condition on macronutrient intake.

**Conclusions**

These preliminary data show that manipulating exercise intensity and timing may influence the anorexigenic effect of exercise given that the HIIT_delay_ condition resulted in decreased absolute EI compared to a control session.

**Acknowledgements**

São Paulo Research Foundation (Grant number 15/11302-3 and 17/07304-6)

## A27 A pharmacokinetic evaluation of chicken protein (Chik│Pro™) as compared to beef protein in heathy active adults

### Douglas Kalman^1,3^, Susan Hewlings^2,3^ Robin Lee^1^, Jacob Bentley^1^, Richard Foster^1^, Kayce Morton^1^

#### ^1^ Departments of BD, Nutrition and Clinical Research. QPS-BKCA, Springfield, MO, 65619, USA; ^2^ Nutrition and Dietetics Department, Central Michigan University, Mt. Pleasant, MI, 48044, USA; ^3^ Substantiation Sciences, Weston, 33326, FL, USA

##### **Correspondence:** Susan Hewlings (sue.hewlings@gmail.com)

**Background**

It is well documented that high quality proteins of various sources stimulate muscle protein synthesis leading to improvements in body composition. Less is known about the comparative pharmacokinetics of these proteins.

**Materials and methods**

This was a prospective, randomized, pharmacokinetic, pharmacodynamic exploratory clinical trial to evaluate the comparative pharmacokinetics and relative effects of Chicken Protein isolate (Chik│Pro™) alone and compared to Beef Protein isolate. The 22 subjects were randomized by Body Mass Index category [19.0 to 26.9 and 27.0 to 34.9] in 2 groups of 11 subjects each. Subjects fasted overnight for at least eight hours and in a single blind fashion consumed 25 grams protein of CP or BP on Day 1 and the alternative treatment after a 3 day washout on Day 4. The blood samples were collected through repeated venipuncture for the amino acids at pre-ingestion (within 1 hour of dose) and post-ingestion at 30, 60, 90, 120,180 minutes. Change from time point 0 was measured by standard statistical techniques for subsequent time points as well as for pharmacokinetic parameters.

**Results**

The Chicken Protein isolate enhanced leucine to a significantly greater and faster degree than the Beef Protein isolate (30 min post 211.73 ± 42.11 vs 178.59 ± 33.57 nmol/mL; p=0.0061; C_max_ 113 ± (43.4) vs 43.4 ± (21.5) nmol/mL AUC_0-180_ 190 ± (68.9) vs 50.6 ± (33.4) h·nmol/mL). The Chicken Protein enhanced Essential Amino Acid absorption and kinetics faster and to a greater degree than the Beef Protein (30 min post 1459.32 ± 293.08 vs 1293.50 ± 185.91, p=0.0251 nmol/mL, C_max_ 663 ± (308) vs 268 ± (181) nmol/mL and AUC_0-180_ 1120 ± (457) vs 329 ± 297 h/nmol/mL). The Chicken Protein delivered more arginine than the Beef Protein (AUC_0-180_ 128 ± (55.4) vs 101 ± (41.2). Chicken Protein delivered more of the Sulfur containing amino acids and at a faster rate than the Beef Protein (30 min post 48.95 ± 14.71 vs 39.18 ± 5.67 nmol/mL; p=0.0170; C_max_ 32.5 ± (11.7) vs 9.33 ± (3.89) nmol/mL and AUC_0-180_ 54.5 ± (17.9) vs 11.9 ± (8.82) h/nmol/mL).

**Conclusion**

Chicken protein isolate (Chik│Pro™) was superior to Beef Protein isolate with greater bioavailability in delivering leucine and essential amino acids faster and to a greater extent to the body than beef protein suggesting it may enhance recovery via protein synthesis faster than beef protein.

**Acknowledgements**

Study Sponsor: International Dehydrated Foods, IDF.

## A28 A randomized, double-blind, placebo-controlled trial of (-)-epicatechin supplementation on adaptations to aerobic and anaerobic endurance exercise training

### Zach J. Blahnik^1^, Neil A. Schwarz^1^, Sarah K. McKinley-Barnard^1^, Shelley L. Holden^1^, Andy Waldhelm^2^

#### ^1^Department of Health, Kinesiology, and Sport, University of South Alabama, Mobile, AL, 36525, USA; ^2^Department of Physical Therapy, University of South Alabama, Mobile, AL, 36525, USA

##### **Correspondence:** Neil A. Schwarz (neilschwarz@southalabama.edu)

**Background**

(-)-Epicatechin supplementation has been shown to increase exercise performance, muscle fatigue resistance, muscle capillarity, and mitochondrial biogenesis in mice. The purpose of the study was to determine if four weeks of cycling combined with (-)-epicatechin supplementation is more effective at increasing aerobic and anaerobic adaptations than cycling combined with placebo.

**Methods**

Nine female and ten male participants with a mean age of 20.7 ± 1.7 years completed the study. Participants completed two testing sessions separated by four weeks of training on a cycle ergometer with twice daily supplementation of 100 mg (200 mg total daily) of 98% pure (-)-epicatechin or placebo (cellulose). For the testing sessions, hemodynamics and body mass were measured followed by a Wingate anaerobic cycle test to assess peak anaerobic power and anaerobic capacity. After the Wingate test, participants rested for 15 minutes and then completed a peak oxygen uptake test on a cycle ergometer. For the exercise training, participants completed four cycling sessions per week following a standardized protocol for a total of 16 exercise sessions. Changes in body mass, hemodynamics, peak VO_2_, peak anaerobic power, and anaerobic capacity were analyzed utilizing mixed-model ANOVA with an *a priori* alpha level of 0.05.

**Results**

A significant difference was observed for time for relative peak anaerobic power (*p* < .01, partial *η*^2^ = .74), relative anaerobic capacity (*p* < .01, partial *η*^2^ = .46), and fatigue index (*p* < .01, partial *η*^2^ = .47). A significant difference was observed for time for absolute peak VO_2_ (*p* < .01, partial *η*^2^ = .48) and peak power output obtained during the peak VO_2_ test (*p* < .01, partial *η*^2^ = .66). A significant interaction between group and time for relative peak VO_2_ was observed (*p* = .04, partial *η*^2^ = .24). Relative peak VO_2_ significantly increased over time in the placebo group (*p* < .01, partial *η*^2^ = .66), but not in the (-)-epicatechin group (*p* = .21, partial *η*^2^ = .19). Furthermore, an independent-samples *t*-test of the difference scores between the pre- and post-tests for relative peak VO_2_ revealed a significant difference between groups (*p* = .04, Cohen’s *d* = 1.06).

**Conclusions**

(-)-Epicatechin supplementation appears to suppress peak relative VO_2_ training response when combined with exercise training on a cycle ergometer. Research into the potential mechanisms inhibiting aerobic adaptations is warranted.

**Acknowledgements**

The authors declare no conflicts of interest. Vital Pharmaceuticals Inc. provided the supplement for the study.

## A29 Effect of acute (-)-epicatechin supplementation on time to complete the 15.5 CrossFit Open Workout

### Brandon R. Funderburg, Neil A. Schwarz, Zach J. Blahnik

#### Department of Health, Kinesiology, and Sport, University of South Alabama, Mobile, AL, 36525, USA

##### **Correspondence:** Neil A. Schwarz (neilschwarz@southalabama.edu)

**Background**

Short-term (-)-epicatechin supplementation has been demonstrated to increase exercise tolerance in rodents. In one human study, seven days of (-)-epicatechin supplementation (25 mg daily) was reported to increase handgrip strength by 7%. The purpose of this study was to determine if acute supplementation of (-)-epicatechin can increase performance during the 15.5 CrossFit Open Workout.

**Methods**

Eleven healthy participants (male = 5, female = 6) completed the study. Average age and body mass index (BMI) of the participants was 26.4 ± 4.8 years and 25.4 ± 4.0 kg/m^2^, respectively. After an entry session to explain the study, participants completed three testing sessions. At each testing session, body mass was recorded followed by the participant completing the 15.5 CrossFit Open Workout for time. In short, the workout consists of four rounds of rowing followed by barbell thrusters. Total time to complete the workout was recorded along with split times for each exercise of each round. The first testing session was treated as a familiarization workout and no supplement was consumed. In a randomized, balanced fashion, 100 mg of 98% pure (-)-epicatechin or cellulose (placebo) was consumed twice daily for two days prior to testing sessions two and three. On the day of testing, 200 mg of the designated supplement was consumed approximately 60 to 90 minutes before completing the workout. Each testing session was performed five to seven days apart at the same time of day for each participant, and participants were asked to keep pre-testing meals consistent between sessions. Statistical analyses were performed using repeated-measures ANOVA with an *a priori* alpha level of 0.05.

**Results**

No significant difference was observed for time to complete the workout between testing sessions (Familiarization = 708 ± 261 s, placebo = 687 ± 250 s, (-)-epicatechin = 684 ± 213 s; *p* > 0.05). Additionally, no difference between testing sessions was observed for split times (*p* > 0.05). Body mass was not significantly different between testing sessions. Further analyses also revealed no influence of testing session order or sex on the time to complete the workout.

**Conclusions**

Acute consumption of 200 mg of 98% (-)-epicatechin prior to the Crossfit 15.5 Open Workout does not appear to increase performance. More research is needed to determine if (-)-epicatechin supplementation is beneficial for other exercise modalities.

**Acknowledgements**

The authors declare no conflicts of interest. Vital Pharmaceuticals Inc. provided the supplement for the study.

## A30 Relationship between select dietary habits, academic achievement, body mass index, and perceived body image among collegiate female track and field athletes

### Shelley L. Holden, Zach J. Blahnik, Neil A. Schwarz

#### Department of Health, Kinesiology, and Sport, University of South Alabama, Mobile, AL, 36525, USA

##### **Correspondence:** Shelley L. Holden (sholden@southalabama.edu)

**Background**

The purpose of this study was to determine the relationship between fast food consumption (FFC), region of college attendance, track and field event participation, body mass index (BMI) classification, grade point average (GPA), living arrangement, perceived body image, and fruit and vegetable intake within a sample of currently active female collegiate track and field athletes.

**Methods**

Current collegiate female track and field athletes (*n* = 165) were surveyed. Continuous data were found to violate the assumption of normality as assessed by the Shapiro–Wilk test (all *p* < 0.05) visual inspection of Q-Q plots. Therefore, group comparisons were performed using Kruskal-Wallis *H* tests and Mann-Whitney *U* tests. Pairwise comparisons for significant Kruskal-Wallis *H* tests were performed using Dunn's procedure with a Bonferroni adjustment. Associations were determined using Spearman's rank correlation coefficient. All analyses were performed with an *a priori* alpha level of 0.05.

**Results**

The South region reported significantly more FFC than the West (*p* < 0.001) and Midwest (*p* = 0.001) regions. Athletes competing in sprints/jumps (*p* = 0.004) and throws (*p* < 0.001) reported significantly more FFC than distance athletes. Athletes who consumed five or more servings of fruit and/or vegetables per day reported significantly less FFC (*p* = .002), had a significantly lower BMI (*p* = .01), and reported a significantly higher GPA (*p* = .027) than those who did not. Obese participants reported significantly greater FFC than those categorized as underweight (*p* = 0.038). A significant positive correlation was observed between FFC and BMI (*p* < .001), and a significant negative correlation was observed between FFC and GPA (*p* = .043). Athletes competing in sprints/jumps (*p* < 0.001), heptathlon/pentathlon (*p* = 0.011), and throws (*p* < 0.001) all had significantly higher BMI than distance athletes. Furthermore, athletes competing in throws had significantly higher BMI than those competing in sprints/jumps (*p* < 0.001). BMI was significantly higher for athletes whose current perceived body type did not match the body type that most appealed to them (*p* = .029).

**Conclusions**

Fast food consumption among female collegiate track and field athletes is related to track event participation, fruit and vegetable consumption, BMI, and GPA. Our results indicate a need for further exploration into the causal relationships between these variables. Additionally, further studies are necessary to determine if these relationships exist in other collegiate sports or in male athletes.

**Acknowledgements**

The authors declare no conflicts of interest.

## A31 A randomized, double-blind, placebo-controlled trial of four weeks of resistance training combined with Bang^®^ Master Blaster^TM^ supplementation on lean body mass, maximal strength, mircoRNA expression, and serum hormones

### Neil A. Schwarz, Sarah K. McKinley-Barnard, Zach J. Blahnik

#### Department of Health, Kinesiology, and Sport, University of South Alabama, Mobile, AL, 36525, USA

##### **Correspondence:** Neil A. Schwarz (neilschwarz@southalabama.edu )

**Background**

The purpose of this study was to determine if Bang^®^ Master Blaster^TM^ (BMB) in conjunction with resistance training increases lean body mass (LBM) and maximal strength more than resistance training while consuming a placebo. Secondly, changes in hemodynamics, skeletal muscle microRNA (miR) expression (miR-15a, -16, -23a, -23b, and -126), and serum hormones (IGF-1 and brain-derived neurotrophic factor, BDNF) were investigated.

**Methods**

Sixteen healthy men with a mean age of 22.5 ± 2.9 years completed the study. Participants completed two testing sessions separated by four weeks of supervised resistance training with supplementation of one serving of BMB or placebo (Fibersol-2) daily (on training and non-training days). For the testing sessions, hemodynamics and body mass were measured followed by a DEXA scan. Venous blood and muscle biopsy samples were collected followed by maximal strength testing (1-RM) for the squat and bench press. For resistance training, participants completed four supervised sessions per week (two lower-body and two upper-body) for a total of 16 sessions. Changes in hemodynamics, body composition, maximal strength, gene expression, and serum hormones were analyzed with an *a priori* alpha level of 0.05.

**Results**

No statistically significant changes were observed for hemodynamics, fat mass, or body fat percentage. A significant interaction between group and time was observed for lean body mass (*p* < .01). Post-hoc analyses revealed a greater increase (*p* < .01) in lean body mass for the BMB group (3.15 ± 1.61 kg) compared with placebo (.89 ± 1.24 kg). A significant interaction between group and time was observed for combined strength (squat + bench 1-RM; *p* = .02) and squat 1-RM (*p* = .04). Post-hoc analyses revealed a greater increase in combined strength for the BMB group (34.38 ± 15.10 vs. 18.75 ± 8.22 kg; *p* = .03). Additionally, a greater increase in squat 1-RM was observed for the BMB group (23.86 ± 8.50 vs.14.20 ± 8.57 kg; *p* = .04). No difference was observed for bench press 1-RM. No significant changes were observed for serum IGF-1 or serum BDNF. No change was observed in miR-15a, -16, -23b, or -126 expression. MicroRNA-23a significantly increased as a result of resistance training (*p* = .03), without any difference between groups (*p* > .05).

**Conclusions**

Compared with placebo, consumption of BMB preferentially increased LBM and maximal strength in combination with short-term resistance training.

**Acknowledgements**

The authors declare no conflicts of interest. Vital Pharmaceuticals Inc. provided funding for the study.

## A32 Acute high-intensity interval and moderate-intensity continuous exercise induces similar responses on ghrelin and energy intake in obese males

### Victor A. F. Matos^1^, Daniel C. Souza^2^, Victor O. A. Santos^1^, Ítalo F. Medeiros^2^, Rodrigo A. V. Browne^2^, Iasmin M. Sousa^2^, Ricardo A. Bezerra^1^, Eduardo C. Costa^1^, Ana P. T. Fayh^1,2^

#### ^1^Graduate Program in Physical Education, Federal University of Rio Grande do Norte, Natal/RN, 00721, Brazil; ^2^Graduate Program in Nutrition, Federal University of Rio Grande do Norte, Natal/RN, 00721, Brazil

##### **Correspondence:** Ana P. T. Fayh (apfayh@yahoo.com.br)

**Background**

Exercise may induce physiological changes in gastrointestinal hormones, including ghrelin, which results in reduced energy intake in normal-weight subjects, and the magnitude of these responses may occur in an intensity-dependent manner [1]. However, these finds regard to different exercise intensities protocols are still lacking in obese population [2,3]. Thus, the present study aimed to determine the magnitude of different exercise intensities (MICE and HIIE) on ghrelin and energy intake in obese males.

**Material and Methods**

In a randomized crossover trial, ten volunteers (31.2 ± 6.4 years, 35.5 ± 4.1 kg/m², 40.1 ± 2.2% body fat) underwent to two experimental conditions: I) MICE (20 min, 70% of maximal heart rate [HR_max_]) and II) HIIE (10 x 1 min, 90% HR_max_ + 1 min active recovery). Total ghrelin was assessed in three moments: 1) pre-exercise, 2) post-exercise and 3) 1h post-exercise. Energy intake was evaluated 1 hour after sessions (*ad libitum* meal offered as a buffet) and throughout the day (24h food record). For statistical analysis a two-way ANOVA with repeated measures (condition vs time) between the experimental sessions was performed to verify total ghrelin concentration and a paired t-test to verify difference on energy intake and macronutrients. A statistical significance of p <0.05 was considered.

**Results**

There was no main effect of the condition x time interaction on ghrelin [F(1, 18)= 0,066, p= 0,859, η^2^_p_=0,007] during exercise sessions, however, a significant effect of time [F(2,18)= 6,016, p= 0,010, η^2^_p_= 0,401] was found (Figure 1). Energy intake and macronutrients did not differ between exercise conditions in ad libitum meal and 24h (Table 1).

**Conclusion**

MICE and HIIE elicits similar effects on total ghrelin and energy intake in obese men with no subsequent increased responses.

**Trial registration:** http://www.ensaiosclinicos.gov.br/rg/RBR-62kr6f/

**Key words:** Obesity; High intensity interval exercise; Hunger; Gastrointestinal hormones.

**References**

1. Hazell TJ, Islam H, Townsend LK, Schmale MS, Copeland JL. Effects of exercise intensity on plasma concentrations of appetite-regulating hormones: potential mechanisms. Appetite. 2016, 1, 80-88.

2. Matos VAF, Souza DC, Browne RAV, Santos VOA, Costa EC, Fayh APT. Acute effect of high-intensity interval exercise and moderate-intensity continuous exercise on appetite in overweight/obese males: a pilot study. Sport Sci Heal. 2017, 13, 403-410.

3. Martins C, Stensvold D, Finlayson G, Holst J, Wisloff U, Kulseng B, Morgan L, King NA. Effect of moderate- and high-intensity acute exercise on appetite in obese individuals. Med Sci Sports Exerc. 2014, 47, 40-8.

**Fig. 1 (abstract A32).**
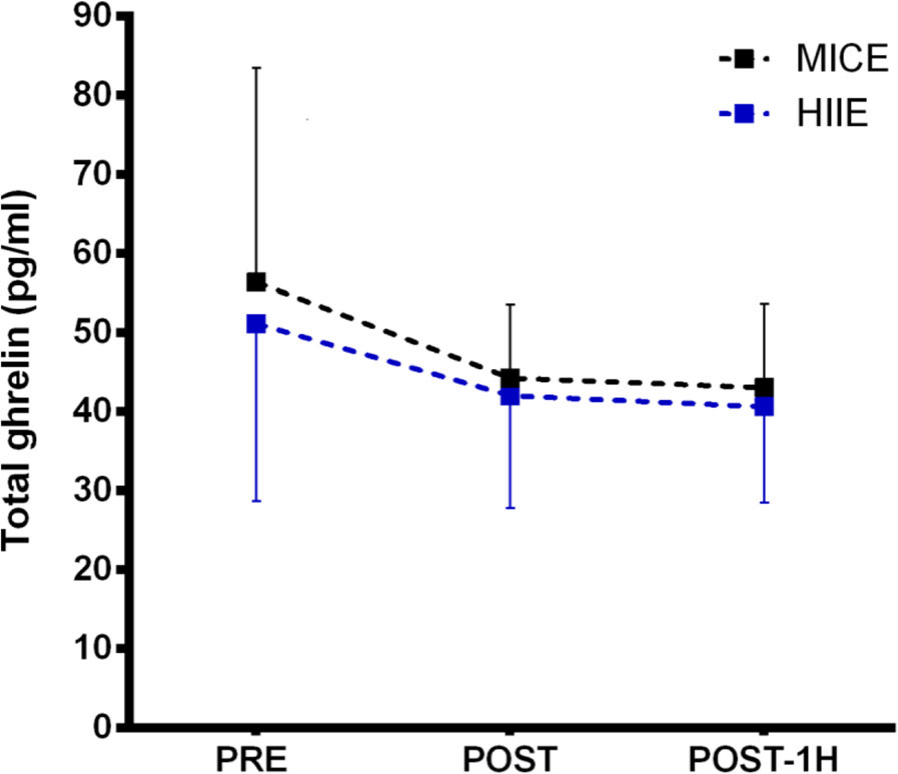
Total ghrelin during exercise sessions, MICE: moderate intensity continous exercise; HIIE: High intensity interval exercise; Values are expressed in mean ± standard deviation.

**Table 1 (abstract A32).** Energy intake and macronutrients in *ad libitum* meal and throughout the day of the experimental sessions

	MICE	HIIE	*p*-value
Energy intake (Kcal)			
*Ad libitum*	743 ± 200	784± 237	0,349
24 h	2886 ± 430	2707 ± 469	0,140
Carbohydrate (%)			
*Ad libitum*	47,46 ± 8,73	48,42 ± 42	0,630
24 h	55,02 ± 5,84	55,82 ± 7,23	0,743
Protein (%)			
*Ad libitum*	14,14 ± 4,19	13,54 ± 3,93	0,055
24 h	16,93 ± 3,74	17,17 ± 4,44	0,881
Lipids (%)			
*Ad libitum*	38,47 ± 7,42	38,05 ± 4,43	0,827
24 h	26,06 ± 5,11	27,03 ± 5,08	0,499

MICE: moderate intensity continous exercise; HIIE: High intensity interval exercise; Values are expressed in mean ± standard deviation. *p*-value with paired t-test.

## A33 The effects of cocoa ingestion on vessel diameter and blood flow in healthy men and women

### Stacie L. Urbina^1^, Katelyn B. Villa^1^, Emily N. Santos^1^, Javier A. Zaragoza^1^, Frank A. Cimino III^1^, Cliffa A. Foster^1^, Lem W. Taylor^1^, Colin D. Wilborn^1^, Rob Wildman^2,3^

#### ^1^Human Performance Laboratory, University of Mary Hardin-Baylor, Belton, TX, 76513, USA; ^2^Department of Nutrition and Food Sciences, Texas Woman’s University, Denton, TX, 76204, USA; ^3^Post Active Nutrition, Dallas, TX, 75207, USA

##### **Correspondence:** Stacie L. Urbina (surbina@umhb.edu)

**Background**

The purpose of this cross-over study was to examine the acute effects of High Epicatechin Cocoa Extract (29000 ppm epicatechin, 44600 ppm theobromine) ingestion on vessel diameter, blood flow, and hemodynamic response.

**Materials and methods**

At baseline testing subjects completed height/weight measurements, questionnaire, body composition, resting hemodynamic measurements, pre- and post-supplementation ultrasound, supplement ingestion with a 30-minute wait period. Ten males and females (21.40 ± 1.67 yrs, 75.87 ± 20.16 kg, 17.65 ± 7.27% body fat) healthy and moderately trained (≥2 months). Body composition was assessed via DEXA and InBody scan were performed in a 3 hour fasted state. Heart rate (HR) and blood pressure (SBP/DBP) was assessed using a digital blood pressure monitor (Omron HEM-907XL) in a supine position. Pre-supplementation ultrasound (S8 Exp, SonoScape Co.,Ltd.) of the brachial artery was performed to assess blood flow peak velocity (PV) and vessel diameter (DIA) and marked to ensure repeated measurements. Subjects were randomly assigned to ingest either 500mg Cocoa (EXP) or maltodextrin (PLC) with 8 ounces of water and remained seated for 30 minutes. All measures were then repeated post-ingestion and an adverse events questionnaire was given. Testing sessions were standardized on time of day and all subjects repeated all testing again 7-10 days

**Results**

Acute ingestion of EXP does not have an effect on various markers of hemodynamic function including vessel DIA, PV, HR, and/or both SBP and DBP.  No significant time (p = 0.309) or group x time interaction (p = 0.427) for average diameter was observed. Mean average diameter across groups decreased (4.16 ± 0.80 vs. 4.06 ± 0.79). No significant time (p = 0.917) or group x time interaction (p = 0.616) for peak velocity was observed. Mean peak velocity across groups increased (106.00 ± 42.65 vs. 106.52 ± 36.36). For SBP and DBP respectively, no significant time (p = 0.785; p = 0.216) or group x time interaction (p = 0.091; p = 0.345) was observed. Mean SBP across groups increased (116.65 ± 11.73 vs. 117 ± 10.55). Mean DBP across groups increased (62.60 ± 7.04 vs. 64.25 ± 6.21). No adverse effects were reported during the trial.

**Conclusion**

Findings of this acute ingestion and double-blind crossover trial suggest that acute ingestion of high Epicatechin Cocoa Extract has no effects on hemodynamic function as assessed by standard measurements and ultrasound.

**Acknowledgement**

This study was supported by an external grant from Dymatize Athletic Nutrition Institute (Dallas, TX).

## A34 The effects of intermittent carbohydrate re-feeds vs. continuous dieting on leptin concentrations in resistance trained individuals: A flexible dieting pilot study

### Danielle Aguilar, Bill I. Campbell, Jaymes Longstrom, Aspen Ranz, Karina Noboa, Abby Fleming, Jamila Lepore, David Mathas, Barbara Sanchez, Andres Toledo, Christina Lodato

#### Performance & Physique Enhancement Laboratory, University of South Florida, Tampa, FL, 33601, USA

##### **Correspondence:** Bill Campbell (bcampbell@usf.edu)

**Background**

There is a direct relationship between blood leptin levels and body weight, such that weight loss is associated with reductions in leptin concentrations. Given its role in regulating hunger and satiety, it is theoretically advantageous to keep leptin concentrations as elevated as possible during a hypocaloric diet. A strategy known as diet “re-feeds” is thought to attenuate some of the adaptive responses to chronic caloric restriction, including reductions in metabolic rate and leptin concentrations. The purpose of this pilot study was to compare leptin levels after 7 weeks of either continuous energy restriction or intermittent restriction with a twice-weekly carbohydrate re-feed.

**Materials and methods**

Resistance-trained males (n=8) and one female (23±3.8 years; 173±7.5 cm; 85±10.8 kg) participated in this pilot study. Participants were randomized to a diet re-feed group (Re-Feed; n=3) or a continuous diet group (CONT; n=5) in conjunction with 4 weekly resistance training sessions for a 7-week period. All participants adhered to a 25% kcal reduction from their baseline calorie intake. The Re-Feed group implemented two consecutive days of elevated CHO intake, followed by 5 days of caloric restriction each week. The CONT group adhered to a continuous 7-week caloric restriction. Leptin concentrations were assessed at baseline and after the 7-week diet. Data were analyzed via a 2-factor [2x2] between-subjects repeated measures ANOVA.

**Results**

No baseline differences for body weight or leptin concentrations existed between the two dietary groups.

Both groups significantly decreased body weight as evidenced by a main effect for time (Re-Feed: baseline=84±4.2kg; post-diet=79.5±5.3kg, Δ4.5kg; CONT: baseline=86.3±14kg; post-diet=81.4±14.6kg, Δ4.9kg; p=0.001). No group x time (p=0.802) or main effect for group (p=0.822) differences were observed for body weight. Both groups significantly reduced leptin concentrations as evidenced by a main effect for time (Re-Feed: baseline=3.8±1.44ng/mL; post-diet=1.33±0.49ng/mL, Δ2.47ng/mL; CONT: baseline= 4.0±0.94ng/mL; post-diet=1.68ng/mL±0.77ng/mL, Δ2.32ng/mL; p=0.001). No group x time (p=0.867) or main effect for group (p=0.632) differences were observed.

**Conclusions**

A 7-week diet at a 25% caloric deficit in conjunction with resistance training resulted in reductions in body weight and plasma leptin levels. Future studies should investigate larger sample sizes to determine if carbohydrate re-feeds during a hypocaloric diet can attenuate decreases in plasma leptin concentrations.

**Acknowledgements**

This investigation was supported by Dymatize Athletic Nutrition Institute (DANI). Bill Campbell is a member of the Dymatize Nutrition Advisory Board.

## A35 The effects of intermittent carbohydrate re-feeds vs. continuous dieting on body composition in resistance trained individuals: A flexible dieting study

### Bill I. Campbell^1^, Danielle Aguilar^1^, Lauren Colenso-Semple^1^, Kevin Hartke^1^, Chris Gai^1^, David Gaviria^1^, John Gorman^2^, Josh Rubio^1^, Adam Ibrahim^1^, Bobby Barker^1^

#### ^1^Performance & Physique Enhancement Laboratory, University of South Florida, Tampa, FL, 33601, USA; ^2^Team Gorman Physique Transformations, Springfield, MO, 65619, USA

##### **Correspondence:** Bill I. Campbell (bcampbell@usf.edu)

**Background**

Physique competitors commonly implement an intermittent approach to fat loss, cycling between periods of caloric restriction interrupted by periods of energy balance. Known as diet “re-feeds,” this strategy is thought to enhance fat loss by attenuating some of the adaptive responses to chronic caloric restriction, including reductions in metabolic rate, glycogen stores, and leptin concentrations. The purpose of this study was to compare body composition changes in resistance trained individuals after 7-weeks of either continuous energy restriction or intermittent restriction with a twice-weekly carbohydrate re-feed.

**Materials and methods**

27 resistance-trained males (n=14) and females (n=13) (25±6.1 years; 169±9.4cm; 80±15.6kg) were randomized to a Re-Feed group (RF; n=13) or Continuous group (CN; n=14) in conjunction with 4-days/week resistance training for a 7-week period. All participants adhered to a 25% kcal reduction from their baseline calorie intake. The RF group implemented two consecutive days of elevated CHO intake, followed by 5-days of caloric restriction each week). The CN group adhered to a continuous 7-week caloric restriction. Body mass (BM), Fat mass (FM), bodyfat% (BF%), and fat-free mass (FFM) were assessed at baseline (following a two-week assessment of maintenance intake) and at the end of the 7-week diet. Data were analyzed via a 2-factor [2x2] between-subjects repeated measures ANOVA.

**Results**

There were no baseline differences in any body composition variable between groups. Main effects for time data revealed that both groups significantly reduced BM (RF: baseline=76.6±15.6kg, post-diet=73.3±13.9kg, Δ3.3kg; CN: baseline=83.3±15.4kg, post-diet=79.6±15kg, Δ3.7kg; p<0.001); FM (RF: baseline=16.3±4kg, post-diet=13.5±3.6kg, Δ2.8kg; CN: baseline=16.7±4.5kg, post-diet=14.4±4.9kg, Δ2.3kg; p< 0.001); BF% (RF: baseline=21.6±4.6%, post-diet=18.8±5%, Δ2.8%; CN: baseline =20.6 ± 6.1%, post-diet=18.6±6.8%, Δ2%; p< 0.001); and FFM (RF: baseline=60.3±13.8kg, post-diet =59.9±13.1kg, Δ0.4kg; CN: baseline=66.6±15.3kg, post-diet=65.3±15.2kg, Δ1.3kg p=0.006). As a follow up (post-hoc test) to the main effect for time, a paired samples t-test revealed a significant reduction in FFM for the CONT group (p=0.001), but not for the Re-Feed group (p=0.460). In addition, there was a statistical trend (p=0.09 group x time interaction) for FFM favoring the Re-Feed group in comparison to the Continuous group.

**Conclusions**

A 7-week 25% caloric reduction in conjunction with resistance training resulted in significant reductions in BM and FM. The Re-Feed group retained more FFM compared to the Continuous group. Future investigations should investigate the mechanisms that potentially explain the extent to which weekly carbohydrate re-feeds contribute to FFM preservation during hypocaloric periods.

**Acknowledgements**

This investigation was supported by Dymatize Athletic Nutrition Institute (DANI). Bill Campbell is a member of the Dymatize Nutrition Advisory Board.

## A36 The effects of intermittent carbohydrate re-feeds vs. continuous dieting on resting metabolic rate in resistance trained individuals: A flexible dieting study

### Bill Campbell, Danielle Aguilar, Vickie Wong, Gavin Rogers, Carl Fox, Tyler Hosey, Shane Lindsay, Sarah Ford, Devin Radford

#### Performance & Physique Enhancement Laboratory, University of South Florida, Tampa, FL, 33601, USA

##### **Correspondence:** Bill Campbell (bcampbell@usf.edu)

**Background**

There is a direct relationship between resting metabolic rate and body weight, such that weight loss is associated with reductions in resting metabolic rate. The implementation of intermittent energy restriction—a strategy known as diet “re-feeds”—is thought to attenuate some of the adaptive responses to chronic caloric restriction, including reductions in metabolic rate and leptin concentrations. The purpose of this study was to compare the relative reductions in resting metabolic rate after 7 weeks of either continuous energy restriction or intermittent restriction with twice-weekly carbohydrate re-feeds.

**Materials and methods**

27 resistance-trained males (n=14) and females (n=13) (25±6.1 years; 169±9.4cm; 80±15.6kg) were matched according to fat mass then randomized to a Re-Feed group (Re-Feed; n=13) or Continuous group (CONT; n=14) in conjunction with 4 weekly resistance training sessions for a 7-week period. All participants adhered to a 25% kcal reduction from their baseline calorie intake. The Re-Feed group implemented two consecutive days of elevated CHO intake, followed by 5 days of caloric restriction each week. The CONT group adhered to a continuous 7-week caloric restriction. Resting metabolic rate was assessed at baseline (following a two-week assessment of maintenance intake) and at the end of the 7-week diet. All resting metabolic rate measurements were assessed one day after the 2-day carbohydrate re-feed. Data were analyzed via a 2-factor [2x2] between-subjects repeated measures ANOVA.

**Results**

No baseline differences for resting metabolic rate were observed between groups. Both groups significantly decreased resting metabolic rate as evidenced by a main effect for time (Re-Feed: baseline = 1,704 ±293 kcal/day; post-diet = 1,664 ±270 kcal/day, Δ40 kcal/day; CONT: baseline = 1,867 ±342 kcal/day; post-diet = 1,789 ±409 kcal/day, Δ78 kcal/day; p = 0.038). A post-hoc paired samples t-test revealed a significant reduction in RMR for the CONT group (p=0.017), but not for the Re-Feed group (p=0.410). No group x time (p=0.482) or main effect for group (p=0.265) differences were observed for resting metabolic rate.

**Conclusions**

A 7-week diet at a 25% caloric deficit in conjunction with resistance training resulted in reductions in resting metabolic rate. An intermittent-restriction approach with a twice-weekly carbohydrate re-feeds was superior to a continuous restriction in preserving resting metabolic rate during the 7-week hypocaloric period.

**Acknowledgements**

This investigation was supported by Dymatize Athletic Nutrition Institute (DANI). Bill Campbell is a member of the Dymatize Nutrition Advisory Board.

## A37 Withdrawn

## A38 Withdrawn

## A39 The effects of αQPC supplementation on weight loss and body composition in overweight, active adults

### Brittany N. Bozzini, Alexa J. Chandler, Thomas D. Cardaci, William G. Hoffman, Christopher E. Ordway, Alan J. Walker, Bridget A. McFadden, David J. Sanders, Shawn M. Arent

#### IFNH Center for Health and Human Performance, Rutgers University, New Brunswick, NJ, 08901, USA

**Background**

Due to the high prevalence of poor body composition and its associated health risks in today’s society, the need for efficacious weight-loss methods becomes increasingly important. Alpha-Glycerophosphocholine (αGPC) is an acetylcholine precursor that may positively influence growth hormone, a major stimulator of lipolysis and protein synthesis. The purpose of this randomized, double-blind clinical trial was to examine the effects of eight-weeks of supplementation with αGPC on weight loss, body composition, and anthropometric measures in overweight, physically active adults.

**Methods**

Participants were randomly assigned to either αGPC supplementation (N=15, M_age_=25.8±9.1yr, M_weight_=82.0±15.1kg, M_height_=163.6±9.6cm) or placebo (N=13, M_age_=24.4±10.4yr, M_weight_=86.0±15.2kg, M_height_=169.5±10.5cm) after health/fitness screening. Both groups were instructed to consume two capsules of their respective supplement for a total of 1200mg/day, one dose before their workout or on non-workout days with their midday meal and the second dose before going to sleep. Assessments were performed pre- and post-supplementation and included body composition via air displacement plethysmography (FM, FFM, BF%, WT), girth measurements (Waist, Hips), and activity level via Framingham Physical Activity Index (Fscore). Participants reported their daily caloric intake in myFitnesspal and were instructed to maintain their current activity level and diet throughout the duration of the study. RM-MANOVAs with univariate follow-ups were conducted with significance set at P<0.05.

**Results**

No significant differences were observed between groups for all body composition and girth measures from pre- to post-supplementation. However, a significant main Time effect was exhibited with decreases in BF%, FM, and Waist (P<0.05). Additionally, trends for decreased WT (P=0.094) and increased FFM (P=0.064) were shown over time for both groups. There were no significant differences (P>0.05) reported in activity level between groups or over time, but a trend for decreased caloric intake (P=0.066) was reported from week one to week eight of supplementation for all participants.

**Conclusion**

Based on the absence of differences between groups, it appears that αGPC supplementation alone does not enhance weight-loss and induce body composition changes in overweight, active adults. The Time effect on body composition and waist measures in participants without changes in activity level demonstrates the potential efficacy of caloric monitoring for weight-loss. Despite the fact that individuals were instructed not to change dietary intake, the improved knowledge of their diet by the daily monitoring could have unassumingly influenced this and thus, the body composition and girth measures over the eight-weeks.

## A40 Eicosanoid and endocannabinoid production with exercise induced by triglyceride metabolism

### David J. Sanders, Joseph K. Pellegrino, Christopher E. Ordway, Alan J. Walker, Bridget A. McFadden, Brittany N. Bozzini, Anthony Poyssick, Marissa J. Bello, Sean P. Conway, Peter J. Gillies, Shawn M. Arent

#### IFNH Center for Health & Human Performance, Rutgers University, New Brunswick, NJ, 08901, USA

**Background**

Lipids are a major energy substrate during exercise, and mobilization of tri, di, and monoacylglycerols (TAG, DAG, MAGs) are needed for ATP production [TAG➔DAG➔MAG➔glycerol +3xFFA]. However, metabolism of these fatty acids (FA) also takes part in eicosanoid and endocannabinoid synthesis, specifically β-MAGs and arachidonoyl-DAGs. Therefore, the purpose of this study was to outline acylglycerol metabolism, particularly as it relates to markers associated with inflammatory mediation.

**Methods**

Participants were equally distributed into groups based on sex (M=20, F=20) and training background (e.g. endurance- [END] or resistance-trained [RES]) (END: M_age_ = 23.3±4.1y, M_height_ = 1.7±0.1m, M_weight_ = 65.2±6.6.1kg, M_%BF_ = 16.7±8.2%; RES: M_age_ = 22.7±2.5y, M_height_ = 1.7±0.1m, M_weigh_t = 69.3±11.3kg, M_%BF_ = 19.4±6.6%). Participants performed 45-minute aerobic cycling (AE) or 45-minute resistance exercise (RE) bouts on separate days. Serum was collected before, immediately post-, and 60-min post-exercise (T_0_, T_1_, and T_2_, respectively) and analyzed via UHPLC/MS by Metabolon for identification of acylglycerol metabolism. RM-ANOVAs were performed comparing time, training background, sex and exercise condition with significance set at P<0.05.

**Results**

Exercise elicited a decrease in β-MAGs (50±17%, p<0.05) and an increase in arachidonoyl-DAGs (28±14%, p<0.05) at T_1_, but not T_2_, regardless of exercise modality, training background, or sex. There were concomitant increases in endocannabinoids (36±26%, p<0.05) and eicosanoids (207±149%, p<0.05) at T_1_. Other MAGs and DAGs were largely unchanged and glycerol was significantly increased for all groups and exercises at T_1_ and T_2_ (73±28% and 32±5% respectively, p<0.05). No differences were seen between sex, training background or exercise condition.

**Conclusions**

Changes observed throughout the arachidonoyl-DAGs and β-MAGs, in conjunction with a lack of net changes throughout the other DAGs and MAGs, and an increase in glycerol suggest an efficient flux though acylglycerol metabolism without buildup of intermediate metabolites. Both of these observations point to funneling of these acylglycerols towards production of eicosanoids [β-MAG➔arachidonyl-DAG➔➔eicosanoids] and endocannabinoids [β-MAGs➔➔endocannabinoids]. This observation is further supported by the increased levels of both of these signaling molecules seen post-exercise. Though regulation of these pathways is not fully understood, our results suggest endocannabinoid and eicosanoid responses may be partially dependent upon substrate metabolism. Further examination of endocannabinoids and eicosanoids role in recovery from physiological stress may help elucidate mechanisms driving various physiological and psychological responses to exercise. These findings were robust across sex, training background and exercise modality.

**Acknowledgements**

Funding provided by the NJIFNH.

## A41 Changes in dietary biomarkers in male and female college soccer players over a full academic year

### Alan J. Walker, Bridget A. McFadden, David J. Sanders, Brittany N. Bozzini, Christopher E. Ordway, Harry Cintineo, Marissa L. Bello, Michelle A. Arent, Shawn M. Arent

#### IFNH Center for Health and Human Performance, Rutgers University, New Brunswick NJ, 08901, USA

**Background**

College athletes often lack the necessary nutritional knowledge to maximize their performance, recovery and health during the competitive season and off-season. Moreover, both aspects of the academic year provide unique challenges in achieving optimal nutrition. The purpose was to identify changes and deficiencies in various dietary biomarkers in male and female college soccer players over the course of a full academic year.

**Methods:**

Male (N=32; M_age_=20.0±1.4yrs; M_weight_=75.4±6.9kg) and female (N=25; M_age_=19.5±1.4yrs; M_weight_=66.1±6.2kg) Division I collegiate soccer players were monitored throughout an academic year. Polar TeamPro was used to assess practices and games by providing training load (TL), distance (DIS), and Kcal expended. Athletes participated in blood draws at the beginning of the competitive season and again during the spring off-season, with 4-week intervals between draws in each phase (C1-C4 & S1-S3, respectively). The morning of their scheduled off-day, athletes arrived fasted and euhydrated for blood draws. Cholesterol (Chol), Omega 3 (OMG3), Vitamin B (Vit-B), Vitamin D (Vit-D), Iron (Fe), and Total Cortisol (Cort) were evaluated. RM-MANOVAs with univariate follow-ups were conducted with significance set at P<.05.

**Results**

During the competitive season, there were significant Time x Sex interactions for TL, DIS, Kcal, Chol, Vit-B, and Vit-D (P<.05) while no interactions were seen during the off-season. Females experienced a significant increase from C1 in OMG3 and Cort (P<.05) and decreases in Chol, Vit-B, Vit-D, Fe during the competitive season (P<.05) with significant increases from S1 in Fe before returning to baseline at S3 (P<.05) and decreases in Chol, OMG3, and Vit-D which returned to baseline at S3 (P<.05) during the off-season. Males experienced a significant increase from C1 in Fe (P<.05) and decrease in OMG3, Vit-D, and Cort during the competitive season (P<.05) with no change in the off-season. Females had a higher TL during C1-C2 with all other time points being similar.

**Conclusion**

Male and female athletes experience a greater degree of change in nutritional status during the competitive season compared to the off-season. There appears to be sex-specific nutritional biomarkers such as Fe for females and Vit-D for males that change in response to times of high TL and may require additional nutritional attention. Females appeared to benefit from a more structured nutritional plan throughout the competitive season than the males. These results show both male and female collegiate athletes may benefit from continued nutrition education, both in-season and off-season, to optimally fuel for performance and recovery.

## A42 The effects of Teacrine and caffeine on endurance and cognitive performance during a simulated match in high-level soccer players

### Marissa L. Bello, Alan J. Walker, Bridget A. McFadden, David J. Sanders, Shawn M. Arent

#### IFNH Center for Health and Human Performance, Rutgers University, New Brunswick, NJ, 08901, USA

**Background**

Theacrine (1,3,7,9-tetramethyluric-acid) is a pure alkaloid with a similar structure to caffeine and acts comparably as an adenosine receptor antagonist. Early studies have shown non-habituating effects, including increases in energy, focus, and concentration in Teacrine®, the compound containing pure theacrine. The purpose of this study was to determine and compare the effects of Teacrine® and caffeine on cognitive performance and time-to-exhaustion during a simulated soccer game in high-level male and female athletes.

**Materials and methods**

Participants (N=24; M_Age_=20.96±2.05y, M_MaleVO2max_=55.31±3.39mL/O_2_/kg, M_FemaleVO2max_=50.97±3.90mL/O_2_/kg) completed a simulated 90-min soccer match protocol on a treadmill, with cognitive testing including simple reaction time (SRT); choice-RT during a go/no-go task; and complex-RT during a dual task of go/no go with distraction math questions at half-, and post-game. Post-game testing was followed by a run to exhaustion at 85% VO_2max_. Participants completed four sessions in randomized order consisting of ingestion of either 275mg teacrine (TCr), 275mg caffeine (Caf), 125/150mg teacrine+caffeine (TCr+Caf), or placebo(P) 30 min prior to the match. Time of day and pre-exercise nutrition was controlled. RM-MANOVAs with univariate follow-ups were conducted and significance was set at P<0.05.

**Results**

Time-to-exhaustion trended toward improvements in all conditions when compared to placebo (ES_TCr_=0.43, ES_Caf_=0.41, ES_TCr+Caf_=0.51). There was a condition main effect (P<0.05) in which Caf (0.60±0.011s) and TCr+Caf (0.59±0.012s) improved choice-RT compared to P (0.61±0.013s). There was a significant Time main effect for complex-RT errors, with improved accuracy at post compared to mid (16.46±2.02 vs. 19.20±2.13). A Time main effect also occurred for SRT, with better RT at mid compared to post (0.64±0.011s vs. 0.65±0.011s). However, a Time x Condition interaction (P<0.05) revealed that P improved from mid to post instead (0.65±0.012s vs. 0.63±0.010s).

**Conclusion**

The 27-38% improvements in time-to-exhaustion reflect an increased performance capacity with these supplements that may have important implications for “added time” scenarios. The larger improvement in choice-RT from TCr+Caf may be due to overlapping peak times for the supplements, leading athletes to sustain greater focus under fatigue for longer periods compared to the other conditions. Peak times may also play a role as the largest SRT improvements occurred at mid compared to post-game; perhaps a higher dosage would cause less of a decline during the transition between Caf and TCr. The improvement seen in accuracy post-game may indicate a training effect for allocation of resources toward the end of a game when players need greater concentration.

**Acknowledgments**

Funding provided by Compound Solutions Inc.

## A43 A comparison of biomarkers and performance between competitive seasons in a women’s division I collegiate soccer program: the impact of a nutrition program

### Bridget A. McFadden, Alan J. Walker, David J. Sanders, Brittany N. Bozzini, Christopher E. Ordway, Harry P. Cintineo, Marissa L. Bello, Michelle A. Arent, Shawn M. Arent

#### IFNH Center for Health and Human Performance, Rutgers University, New Brunswick, NJ 08901, USA

##### **Correspondence:** Bridget A. McFadden (bridget.mcfadden@rutgers.edu)

**Background**

Adequate nutrition is essential yet often undervalued aspect of training. The high caloric-expenditure of female soccer players points to the need for nutrition interventions designed to maximize recovery and enhance performance. The purpose of this study was to assess the impact of a systematic sport nutrition program on DI female soccer players.

**Methods**

Female soccer players (N_S1_=26, N_S2_=25; M_weightS1_=65.6±5.8kg, M_weightS2_=66.1±6.4kg; M_heights1_=167.8±6.3cm, M_heights2_=168.7±7.3cm) participating in a DI collegiate program were monitored for two consecutive competitive seasons (S1&S2). A sport nutrition program was implemented throughout S2 and included an emphasis on post-training nutrition and educational seminars. Fitness tests, including body composition to assess fat-free mass (FFM) and percent bodyfat (%BF), vertical jump (VJ), and VO_2max_, were performed pre- and post-season. Blood draws were performed prior to the start of preseason and every 4-weeks thereafter (T1-T4). Athletes arrived fasted and euhydrated in the morning 18-36 hours post-game. Free cortisol (FCORT), creatine kinase (CK), growth hormone (GH), insulin-like growth factor-1 (IGF1), interleukin-6 (IL6) were analyzed. Polar TeamPro was used to assess practices and games by providing training-load (TL), distance (DIS), and Kcal/Kg. RM-MANOVAs with univariate follow-ups were conducted with significance set at P<0.05.

**Results**

There were no differences at baseline between S1 and S2 for VJ, FFM, or %BF. However, S1 athletes entered the season with a higher VO_2max_ (P<0.05). Throughout S1, there were decreases in VJ (∆VJ=-2.37±1.2cm), VO_2max_ (∆VO_2max_=-2.11±0.8ml/kg/min) and %BF (∆%BF=-1.38±0.5) along with an increase in FFM (∆FFM=1.45±0.7kg) (P<0.05). In contrast, all fitness measures were maintained throughout S2. Despite greater TL (P<0.05) and trends for greater Kcal/kg (P=0.067) in S1, there was no difference in DIS. S2 had lower FCORT at T2, but elevated FCORT at T4. However, S2 had consistently lower IL-6 throughout the season (T1,T2,T4:P<0.05; T3:P=0.088). Additionally, S2 displayed greater IGF-1 at T3 (P<0.05). The only differences in CK occurred at the beginning of preseason (T1), with higher values in S2 (P<0.05). No differences were seen at any time-points for GH.

**Conclusion**

The external load on the athletes (i.e.,DIS) was similar between seasons. However, energy-expenditure, TL, and inflammation were mitigated in S2, suggesting the athletes exhibited improved physiological efficiency and recovery capability. This is particularly relevant given S2 athletes were better able to maintain performance outcomes compared to S1 who demonstrated decrements in VJ and VO_2max_ pre-to-post. This points to the efficacy of a systematic sports nutrition program and the need to further educate athletes on proper fueling strategies.

**Acknowledgements**

Funding provided by Quest Diagnostics

## A44 Characterization of the amino acid metabolomic response to acute aerobic and resistance exercise as a function of training background

### Alexa J. Chandler, Joseph K. Pellegrino, Christopher E. Ordway, Sean P. Conway, Alan J. Walker, Bridget A. McFadden, David J. Sanders, Anthony N. Poyssick, William Hoffmann IV, Peter J. Gillies, Shawn M. Arent

#### IFNH Center for Health and Human Performance, Rutgers University, New Brunswick NJ, 08901, USA

**Background**

Amino acids (AAs) play an essential role in numerous physiological functions. Many different factors can influence AA metabolism, including exercise mode, training background, and/or sex. The purpose of this study was to examine the effects of two different acute exercise bouts on AA metabolism in endurance versus resistance-trained individuals.

**Materials and methods**

Participants were split evenly into two groups based on training background and sex: endurance (END, n=20, M_age_=23.3±4.1y, M_weight_=65.2±6.61kg) and resistance (RES, n=20, M_age_=22.7±2.5y, M_weight_=69.3±11.3kg) trained individuals. All participants completed 45 min aerobic cycling (AE) and resistance exercise (RE) sessions on separate days. Serum was collected pre-, 0-minutes post-, and 60-minutes post-exercise (T_0_, T_1_, T_2_), and analyzed for markers of AA and N-acetylated AA metabolism via UHPLC-MS/MS by Metabolon. RM-ANOVAs for sex, training status, exercise type, and time were conducted with significance set at P<0.05

**Results**

Both RES and END had decreased serum AAs following acute exercise, with RES experiencing a greater number of overall significant decreases (39.7%) compared to END (20.7%) after both exercise bouts from T_0_ to T_1_. Exceptions to this included increases in AAs involved in the glucose-alanine cycle (alanine: 30±9%, P<0.05), TCA cycle (aspartate:18±5%, P<0.05; glutamate:41±21%, P<0.05), and antioxidant functions (cysteine:61±15%, p<0.05; taurine:24±9%, P<0.05) for all groups/conditions. Urea cycle metabolites decreased (arginine: 92±4%, P<0.05; ornithine: 89±4%, p<0.05) and argininosuccinate increased (137±83%, P<0.05) from T_0_ to T_1_. All markers returned towards baseline by T_2_. N-acetylated AAs were increased at T_1_ compared to T_0_, and remained elevated at T_2_ for all groups and conditions (52±38%, P<0.05). Both RES and END had decreased AA levels at T_2_ compared to T_0_, with no differences between groups or exercise mode. No sex differences were identified.

**Conclusions**

Decreased AA levels and increased urea cycling at T_1_ and T_2_ compared to T_0_ after both AE and RE suggests AA uptake and energy use during exercise. Decreases in serum AA may signal N-acetylation to counter AA catabolism**.** Greater AA metabolism in RES at T_1_, but not T_0_, suggests enhanced exercise-induced enzyme up-regulation for protein use linked to training or dietary differences. Training background appears to have a greater influence on energy pathways used during exercise compared to the acute exercise mode. These findings should be taken into consideration when implementing nutrition plans in conjunction with exercise programs.

**Acknowledgements**

Funding provided by the NJIFNH.

## A45 Carbohydrate-specific metabolomic flux following aerobic and resistance exercise in trained individuals

### Harry P. Cintineo, Joseph K. Pellegrino, Christopher E. Ordway, Marissa L. Bello, Alan J. Walker, Bridget A. McFadden, Sean P. Conway, David J. Sanders, Anthony N. Poyssick, William G. Hoffmann IV, Peter J. Gillies, Shawn M. Arent

#### IFNH Center for Health and Human Performance, Rutgers University, New Brunswick, NJ 08901, USA

**Background**

Carbohydrate (CHO) serves as a major substrate for energy during both resistance exercise and aerobic exercise. The Cori and glucose-alanine cycles mobilize glycolytic substrates, while the pentose phosphate pathway (PPP) shunts CHO from glycolysis and protects against oxidative stress. Our purpose was to identify the flux of the CHO-specific exercise metabolome to better understand the role of these pathways following different exercise modalities.

**Materials and methods**

Males (N=20, M_age_=24±4yrs, M_BF%_=12.8±5.7%) and females (N=20, M_age_=22±2yrs, M_BF%_=23.3±4.8%) were equally distributed into groups based on training background [endurance- (END) or resistance-trained (RES)]. On separate days, 45-minute aerobic cycling (AE) or resistance exercise (RE) was performed. Serum was collected pre-, 0-, and 60-min post-exercise (T0, T1, and T2) and analyzed via UHPLC/MS by Metabolon for identification of markers of CHO metabolism. Principle components analysis established metabolite profiles. RM ANOVAs for sex, training status, exercise type, and time were conducted with significance set at P<0.05.

**Results**

Serum glucose (GLU), pyruvate (PYR), lactate (LAC), and alanine (ALA) were elevated immediately post-AE and RE (19±11%, P<0.05; 247±73%, P<0.05; 293±103%, P<0.05; 30±9%, P<0.05). PYR, LAC, and ALA remained elevated at T2 (54±24%, P<0.05; 61±24%, P<0.05; 14±7%, P<0.05). LAC and ALA were more elevated following RE than AE at T1 (62%, P<0.05; 7%, P<0.05), and LAC was still more elevated following RE than AE at T2 (22%, P<0.05). LAC was also more elevated in RES than END immediately (49%, P<0.05) and 60-min post-AE and RE (39%, P<0.05). No sex differences were found. Serum ribose (RIB) decreased following RE at T1 (32±21%, P<0.05) and T2 (46±17%, P<0.05) and remained unchanged post-AE. Sugar alcohols (SA) of the PPP increased to a greater magnitude immediately post-AE than RE (28±21% vs. 18±17%).

**Conclusions**

Elevated GLU, PYR, LAC, and ALA post-exercise display the reliance on CHO via glycolysis, Cori cycling, and glucose-alanine cycling during and following AE and RE. Larger increases in LAC and ALA following RE compared to AE indicate greater glycolytic flux and transamination with this exercise mode. Differences in the RES/END cohorts suggest a training response for upregulation of these processes in RES. Lower RIB and SA post-RE compared to AE show a decreased rate of PPP activity and NADPH production, which functions to reduce reactive oxygen species through glutathione activity. Ultimately, these findings have implications for peri-exercise feeding and supplementation strategies.

**Acknowledgments**

This study was funded by the NJIFNH

## A46 Effect of sex, training background and exercise modality on the pattern of SFA, MUFA, and PUFA mobilization

### Christopher E. Ordway, Joseph K. Pellegrino, Sean P. Conway, Alan M. Walker, Anthony N. Poyssick, David J. Sanders, Bridget A. McFadden, Brittany N. Bozzini, Marissa L. Bello, William G. Hoffmann IV, Peter Gillies, Shawn M. Arent

#### IFNH, Center for Health and Human Performance, Rutgers University, New Brunswick, NJ, 08901, USA.

**Background**

Aerobic and resistance exercise elicit differential acute responses leading to chronic physiological adaptations. This may include specific patterns of fatty acid (FA) mobilization for the working muscle. The purpose of this study was to characterize FA mobilization by saturation status of the acyl chain in response to exercise in trained individuals.

**Materials andmethods**

Male (N=20, M_age_= 24.00±4.0y, M_weight_= 72.94±6.9Kg) and female (N=20, M_age_= 22.10±2.3y, M_weight_= 61.58±7.4Kg) participants were divided into four equal groups (N=10) based on sex as well as endurance (END) or resistance (RES) training background. Participants completed 45 min of either aerobic cycling (AE) or resistance exercise (RE) on separate days. Serum was collected pre, immediately after, and 60-minutes post-exercise (T_0_, T_1_, T_2_, respectively), and was analyzed by UHPLC-MS/MS by Metabolon for saturated (SFAs), monounsaturated (MUFAs), and polyunsaturated (PUFA) fatty acids. RM-ANOVAs were conducted on log-transformed-median-scaled outputs. Metabolomic heat-maps were generated. Significance was set at P<0.05.

**Results**

SFA mobilization increased during AE (P<.05), but not RE (Increase T_0_→T_1_ AE: 37±63%; RE: 10±43%). This differential response was mirrored in MUFAs and PUFAs (T_0_→T_1_ AE: 49±80% & 29±67%; RE: -3±23% & -6±28%, respectively). By T_2_, this difference was diminished for SFAs (AE>RE: T_1_: 45±61%, T_2_: 19±32%), MUFAs (AE>RE: T_1_: 78±91%, T_2_: 24±22%) & PUFAs (AE>RE T_1_: 53±54, T_2_: 18±15%). This finding was consistent across sex and training background, though PUFAs demonstrated this pattern to a greater degree in females than in males (AE>RE: F: T_1_: 65±77%, T_2_: 10±17%; M: T_1_: 39±40%, T_2_: 30±22%). In males, END displayed a more consistent mobilization response between bouts than RES (T_1_: AE>RE by 19±33% for END and 85±93% for RES).

**Conclusion**

FA mobilization was apparent during AE where intensity was conducive to the utilization of FAs as a substrate, but not RE where the higher intensity precludes this. During recovery, FA levels begin to equate as they increased post-RE and returned to baseline values post-AE. Despite differences between AE and RE, SFAs, MUFAs and PUFAs all displayed identical responses within each respective exercise bout.

**Acknowledgements**

Funding provided by the NJ institute for Food, Nutrition & Health.

## A47 The effects of sex and exercise mode on substrate partitioning and metabolism in trained individuals

### Thomas D. Cardaci, Joseph K. Pellegrino, Christopher E. Ordway, Sean P. Conway, Alan J. Walker, Marissa L. Bello, Anthony N. Poyssick, David J. Sanders, Bridget A. McFadden, Brittany N. Bozzini, Alexa Chandler, William Hoffman IV, Peter J. Gillies, Shawn M. Arent

#### IFNH, Center for Health and Human Performance, Rutgers University, New Brunswick, NJ, 08901, USA

**Background**

The bioenergetic demands of exercise are influenced by a variety of factors including sex and exercise modality. Metabolomic profiling may help with a more robust understanding of down-stream effects of nutrient utilization under varying conditions. The purpose of this study was to investigate the effects of sex and mode of exercise on substrate partitioning and metabolism following an acute exercise bout.

**Materials and methods**

Trained participants were equally distributed into groups based on sex (males [M]: n=20, M_Age_=24±4y, M_weight_=72.8±7.8kg, M_BF%_=12.8 + 5.7%; females [F], n=20, M_Age_=22±2y, M_weight_=61.5±.2kg, M_BF%_=23.3 +4.8%). On separate days, 45-min aerobic cycling (AE) or resistance exercises (RE) were performed. Serum was collected pre-, immediately after, and 60-minutes post-exercise (T0, T1, & T2), and analyzed via UHPLC-MS/MS by Metabolon for identification of long chain fatty acids (LCFA), branched-chain amino acids (BCAA) and markers of carbohydrate (CHO) metabolism. RM-ANOVA’s for sex, exercise mode, and time were run with significance set at P<0.05.

**Results**

At T1, F had significantly higher serum LCFA compared to M following AE (F>M: 69±23%, P<0.05) and similar increases at T2 in RE (F>M: 74±20%, P<0.05). Moreover, F had higher levels of ketone bodies at T2 than M with a greater magnitude following AE compared to RE (3-betahydroxybutyrate, 347±172% vs. 116±63%, respectively, P<0.05). The data showed a trend (P<0.1) for M experiencing a greater perturbation in metabolites of BCAA and CHO both at T1 and T2. This did not appear to differ as a function of exercise modality.

**Conclusions**

It appears females metabolize LCFA to a greater extent than males, specifically during AE and during recovery from RE. We also observed evidence that males rely more heavily on a mixture of BCAA and carbohydrate metabolism during and after exercise compared to females, perhaps balancing the difference observed in LCFA energy derivation. These findings may be dependent upon sex-differences in hormonal milieu, and/or different depots for fat and glycogen, as lean body mass differs between men and women. More research is needed to understand the sex-based differences in fuel metabolism during and following exercise.

**Acknowledgments**

Funding provided by NJ Institute for Food, Nutrition, & Health

## A48 Efficacy of fenugreek supplementation on men’s health: a randomized placebo-controlled double-blind trial

### Kevin RM. Coyle, Emily Barton, Heather A. Hausenblas, Kara L. Conway, Terrence Orange, L. D. Smith, Michael Esposito, B. Fry, C. Harvey, D. Oakes, C. Bergman, David R. Hooper

#### Jacksonville University, Brook Rehabilitation College of Healthcare Sciences, Jacksonville, FL, 32034, USA

**Background**

Fenugreek (Trigonella foenum-graecum L.) is a leguminous plant that has historically been used in Indian, North African, and Arabic regions as a dietary supplementation for its proposed health benefits. In recent years fenugreek has started to be cultivated worldwide and used as an anti-inflammatory, anti-diabetic, antiseptic, and libido booster. Limited quality research has been done examining the significance of the effects of fenugreek supplementation to improve men’s health, particularly aging male symptoms (AMS) and health-related quality of life (HRQoL). This was an 8-week randomized double-blind placebo controlled trial examining the efficacy of daily concentrated fenugreek seed extract (AlphaFen®) supplementation on healthy men’s total testosterone (TT), health-related quality of life, grip strength, and male aging symptoms.

**Materials and methods**

The participants (N = 57, M age = 26.05±5.77 years) were randomized to one of the following three conditions: AlphaFen® fenugreek (400 mg/d; N = 19), AlphaFen® fenugreek (500 mg/d; N = 19), or Placebo control (N = 19). Assessments were completed at Day 0, Day 30, and Day 60. Participants completed self-report questionnaires for the HRQoL and AMS during each testing visit. In addition, during the Day 0, 30, and 60 visits blood was drawn *via* venipuncture of an antecubital vein and later analyzed by enzyme-linked immunosorbent assay (ELISA) for TT. Repeated measures ANOVAs were used to determine significance (α=0.05).

**Results**

The fenugreek conditions reported significant improvements in grip strength, quality of sleep, aging male symptoms, and total testosterone compared to the control group (p < 0.05). Significant increases in grip strength were reported (p = 0.02) with 71% of individuals in the treated group showing an improvement. In respect to sleep quality 68% of individuals in the placebo compared to 95% of those in the supplemented groups reported a reduction in the number of days they felt that they did not get enough sleep (p = 0.03). Dose-response significantly affected sleep quality (p < 0.05). Significantly more participants had a positive change in their AMS Total sores in treated groups (p < 0.01), as well the treated groups demonstrating significant improvements in the sexual subscale over placebo (p = 0.05). TT increased significantly in the treated groups over placebo (p = 0.05).

**Conclusion**

Fenugreek supplementation is safe and effective for improving aging male symptoms, total testosterone concentrations, sleep quality and grip strength in healthy recreationally active men. Future researchers are encouraged to examine the health and ergogenic effects of fenugreek supplementation in hypogonadal and older populations.

**Acknowledgements**

This work was supported by a grant from Specnova , Inc (Boca Raton, FL), the makers of AlphaFen ®.

## A49 Energy system specific analytical techniques for Wingate anaerobic capacity tests

### Geoffrey M Hudson^1,2^, Avinash Chandran^2^, Anthony D Garber^3^.

#### ^1^Department of Health, Kinesiology, and Sport, University of South Alabama, Mobile, AL, 36688, USA; ^2^Department of Exercise and Nutrition Sciences, The George Washington University, Washington, D.C., 20052, USA; ^3^Department of Physical Therapy and Rehabilitation Science, University of Maryland, Baltimore, MD, 21201, USA

##### **Correspondence:** Geoffrey M Hudson (GHudson@southalabama.edu)

**Background**

The Wingate Anaerobic Test (WAnT) is used to evaluate anaerobic capacity. However, the traditional data provided by WAnT such as peak power (PP), anaerobic capacity, fatigue index (FI), and total work provide an incomplete picture of the energy systems utilized during this (typical) 30 second test. Specifically, the rate at which the power output changes during the test would provide helpful information about the energy systems utilized. Fatigue rate (FR) has been recently introduced and represents the rate that power decreases during a test. We combined FR measures and calculated area under the curve (AUC) of bioenergetic specific intervals of the WAnT output in order to provide novel analytical techniques that can be utilized to evaluate the effectiveness of exercise training programs and/or ergogenic aids designed to augment anaerobic exercise performance.

**Materials and methods**

Data from 30s WAnT tests (torque factor of 0.075) were analyzed for 37 physically active college students (22 female; mass: 73.3±12.2 kg). Data were analyzed with SAS software. The 30s (power at every 0.1 sec) of the WAnT test was divided into three sequential eight second intervals (P, PG, G) following each subject’s PP. Generally, interval P was from 1.0-8.9 s, PG from 9-16.9 s, and G from 17-24.9 s (Figure 1). Time intervals were compared using ANOVA with repeated measures, paired t-test post-hoc analyses, alpha of 0.05, and are presented as means ± SD.

**Results**

FR was significantly different for all three time intervals P, PG, G: -37.5±15.3, -25.6±7.9, and -15.0±8.1 W·s^-1^ (p<0.001). The AUC of each interval also decreased: 7305.1±1854.2, 5321.5±1577.1, and 4075.2±1297.4 J (p<0.001). FI was significantly lower (p<0.001) at interval G (21.0±8%) than P or PG (26.0±8, 26.0±8).

**Conclusions**

Information gleaned from changes in PP can be limited and there are limitations in comparing FI from WAnT tests in exercise or dietary interventions. Highlighting segments of the WAnT that correspond to the phosphagen, glycolytic, and the transition between the two systems can provide new, more meaningful data for interventions designed to improve anaerobic performance. Future studies will utilize mixed effects modeling to provide additional information about change in slope during these time intervals following sprint training and dietary supplementation.

**Acknowledgements**

Authors have no conflicts of interest.

**Fig. 1 (abstract A49).**
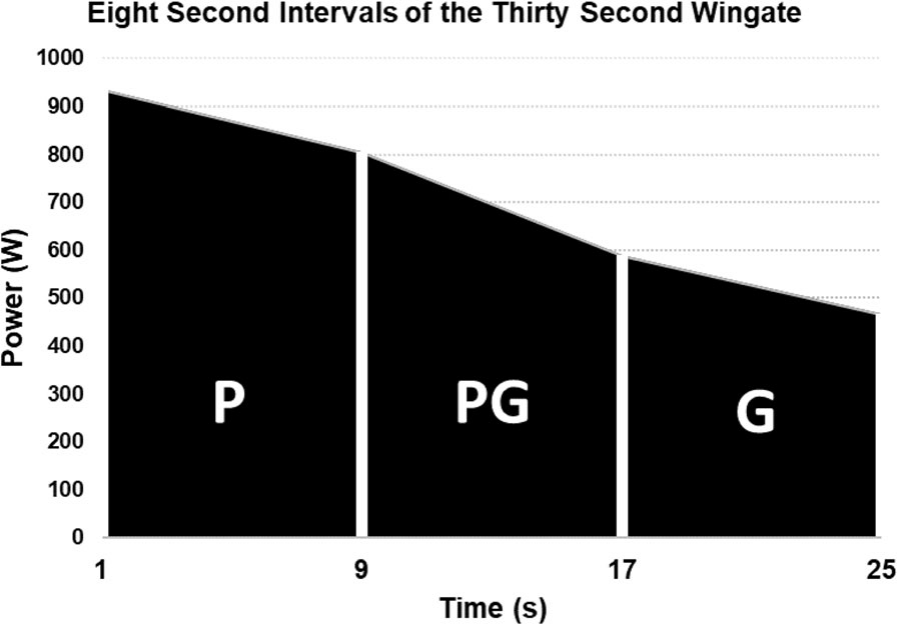
Example of eight second intervals delineated for a thirty second Wingate. P represents the phosphagen system, G glycolytic system, and PG the transition between the two systems.

## A50 An evaluation of the effects of inositol-stabilized arginine silicate (ASI; Nitrosigine®) in preventing the decline in cognitive function caused by strenuous exercise

### Malkanthi Evans^1^, Nisrine Zakaria^1^, Mohammed Marzuk^1^, Sara Perez Ojalvo^2^, Sarah Sylla^2^, James Komorowski^2^

#### ^1^KGK Science Inc, London, Ontario, N6A 5R8, Canada; ^2^Nutrition N21, LLC, Purchase, NY 10577, USA

##### **Correspondence:** James Komorowski (jkomorowski@nutrition21.com)

**Background**

Inositol-stabilized arginine silicate (ASI; Nitrosigine®) is a popular pre-workout ingredient that has been shown to increase nitric oxide production, blood flow, muscle recovery, energy, and cognitive function. ASI has been shown to significantly increase mental acuity, focus and processing speed within 15 minutes of taking a single dose [1]. However, the effect of ASI on cognitive function following exercise had not yet been evaluated. Because intense physical activity can cause mental fatigue which can then have a negative impact on decision making and physical performance, preventing cognitive impairment would be advantageous for athletes in maintaining focus and mental acuity. A randomized, double-blind, placebo-controlled, crossover study evaluated the effects of an acute dose of ASI (1,500 mg) on cognitive function following intense aerobic exercise.

**Materials and methods**

Twenty-four healthy male adults 18-40 years of age and BMI 18.5-30 kg/m^2^ were randomized equally to two study arms, separated by a 2-week washout period. Participants took a single dose of ASI (1,500 mg) or placebo 30 minutes prior to a treadmill maximally Graded Exercise Test (mGXT). A Trail Making Test (TMT), composed of two parts (TMT-A and TMT-B) was used to measure cognitive function prior to dosing and immediately after exercise. The time to complete the TMT measured mental acuity, focus and processing speed, with an increase in time indicating a decrease in cognitive function, and a decrease in time indicating an improvement in cognitive function.

**Results**

A single dose of ASI significantly improved cognitive function parameters of mental acuity, focus and processing speed after intense exercise, compared to placebo (p ≤ 0.05). Following strenuous exercise, time to complete TMT-A and TMT-B increased by a significant 51% and 11% respectively in the placebo group, while it decreased by 5% for TMT-A and 7% for TMT-B when participants consumed an acute dose of ASI (p ≤ 0.05; Figure 1).

**Conclusions**

The results of this study showed that ASI prevents the decline in cognitive function seen following strenuous exercise. Acute consumption of ASI prevented the intense exercise induced cognitive function decline of 51% seen in the placebo. These results could be of interest to individuals looking to maintain a strong cognitive state after expending energy during intense athletics, as well as everyday life.

**Acknowledgements**

This study was conducted at KGK Science Inc. and funded by Nutrition 21, LLC.

**References**

1. Kalman D, Harvey PD, Perez Ojalvo S, Komorowski J. Randomized prospective double-blind studies to evaluate the cognitive effects of inositol-stabilized arginine silicate in healthy physically active adults. *Nutrients* 2016, 8:736.

**Fig. 1 (abstract A50).**
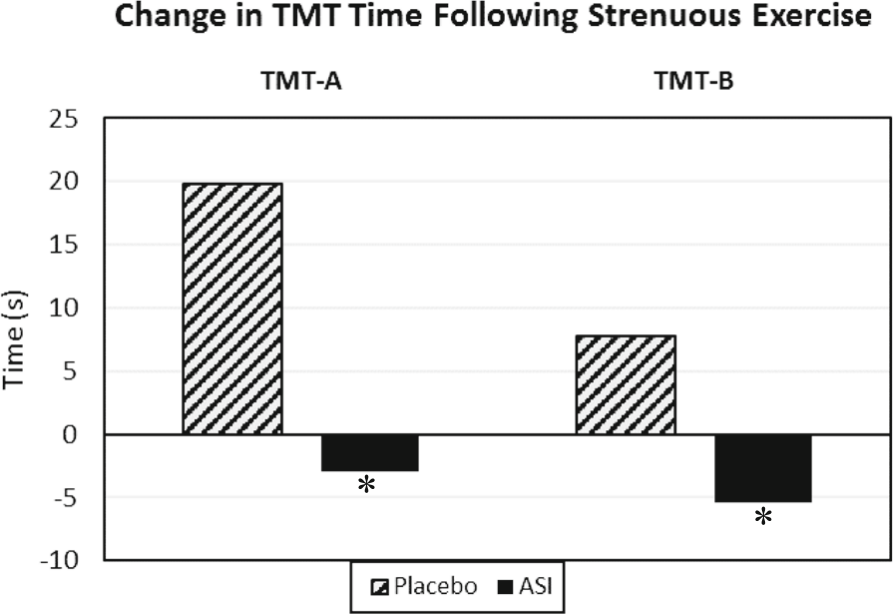
This graph compares the change in TMT-A and TMT-B times following strenuous exercise in the ASI group verses placebo. In the placebo group, TMT-A and TMT-B times increased by 19.8 and 7.7 seconds respectively, while time decreased by 2.8 seconds for TMT-A and 5.5 seconds for TMT-B in the ASI group (*p ≤ 0.05 between groups).

## A51 The effect of a proprietary maca powder (Maca-N21) on endurance capacity in exercised rats

### Sarah Sylla^1^, Sara Perez Ojalvo^1^, James Komorowski^1^, Cemal Orhan^2^, Nurhan Sahin^2^, Kazim Sahin^2^

#### ^1^Nutrition 21, LCC, Purchase, NY 10577, USA; ^2^Firat University, Elazig, Turkey

##### **Correspondence:** James Komorowski (jkomorowski@nutrition21.com)

**Background**

*Lepidium meyenii*, commonly known as maca, is a Peruvian plant that has been used for centuries to enhance mood, libido, and energy. Maca-N21 is a propriety blend of maca that has been shown to enhance cellular energy production and the activity of signaling proteins involved in muscle energy metabolism. The purpose of this preclinical study was to assess the effect of Maca-N21 on endurance capacity in exercised rats.

**Materials and methods**

Twenty-eight Sprague-Dawley rats (age: 8 weeks, weight: 180 ± 20 g) were randomly divided into four groups (n=7 per group): 1) Control (vehicle), 2) Maca-N21 powder (40 mg/kg body weight), 3) Exercise control (vehicle), 4) Exercise + Maca-N21 powder (40 mg/kg body weight). Groups received treatment via oral gavage once per day for 21 consecutive days. All rats completed a swimming acclimation schedule. On the 14^th^ day of the experiment, 30 minutes after administration of study product, a weight-loaded forced swim test (5% body weight) was employed and rats were timed to exhaustion. On the 21^st^ day of the experiment, after administration of study product and a non-loaded swim test, rats were sacrificed. Serum and tissue samples were collected to measure levels of lactate and oxidative markers such as superoxide dismutase (SOD), glutathione peroxidase (GSH-Px), and malondialdehyde (MDA).

**Results**

Swimming time to exhaustion in the Exercise + Maca-N21 group was 44% higher than the Exercise group (p < 0.05) (Figure 1). Following exercise, serum lactate levels were 42% lower in the Exercise + Maca-N21 group (12.74 mg/dL) compared to the Exercise group (21.89 mg/dL) (p < 0.05). Both exercised and non-exercised rats supplemented with Maca-N21 had lower levels of oxidative stress marker MDA in the serum, liver, and muscle compared to corresponding control groups (p < 0.05). Comparing exercise groups, muscle MDA levels were 19% lower in the Maca-N21 group (p < 0.05). Moreover, levels of muscle GSH-Px, an enzyme known to protect against oxidative damage, were higher in the Maca-N21 groups compared to corresponding control groups (p < 0.05). Comparing exercise groups, muscle GSH-Px levels were 44% higher in the Maca-N21 group (p < 0.05).

**Conclusions**

The results of this preclinical study showed that Maca-N21 significantly improved swimming time to exhaustion in rats, as well as serum lactate and oxidative stress marker levels after exercise. These results support the use of Maca-N21 as an anti-fatigue and endurance enhancing ingredient for sports nutrition.

**Acknowledgements**

This study was conducted at Firat University and funded by Nutrition 21, LLC.

**Fig. 1 (abstract A51).**
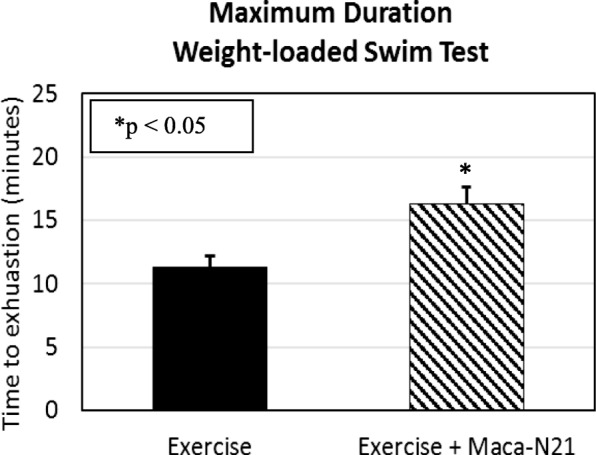
See text for description

## A52 An evaluation of the bioavailability and cognitive effects of a novel magnesium complex (Mg-N21) in high fat fed rats

### James Komorowski^1^, Sara Perez Ojalvo^1^, Sarah Sylla^1^, Cemal Orhan^2^, Mehmet Tuzcu^2^, Nurhan Sahin^2^, Kazim Sahin^2^

#### ^1^Nutrition 21, LLC, Purchase, NY 10577, USA; ^2^Firat University, Elazig, 23119, Turkey

##### **Correspondence:** James Komorowski (jkomorowski@nutrition21.com)

**Background**

Magnesium (Mg) is a mineral that plays an important role in many physiological functions and recently has been shown to improve learning and memory. Suboptimal magnesium intake, which is associated with various health issues, is common in the average American diet and therefore supplementation with bioavailable forms of magnesium is important for adequate magnesium intake and optimal health. The following preclinical study was carried out to compare the bioavailability and effects on learning and memory of magnesium oxide (MgO), a commonly used form of magnesium, to Mg-N21, a novel magnesium complex.

**Materials and methods**

Forty-two male Wistar rats (age: 8-week, weight: 180 ± 20 g) were randomly divided into the following groups (n=7 per group): Control (no treatment), MgO, Mg-N21, HFD (fed a high fat diet), HFD+MgO, and HFD+Mg-N21. Rats were supplemented for 8 weeks. Magnesium was dosed at 500 milligrams of elemental Mg/kg diet. Rats completed a Morris water maze four times a day for six consecutive days, with swimming pathway and latency in locating a hidden platform recorded for each trial. On the seventh day, rats were tested with the platform removed, with increased memory demonstrated by more platform crossings and time in target area. At the end of treatment, plasma and tissue samples were obtained for analysis.

**Results**

After 8 weeks, serum and liver magnesium levels were higher in both Mg-N21 groups verses corresponding control and MgO groups, while fecal levels were lower verses MgO groups (p < 0.05), indicating greater absorption and retention. Brain magnesium levels were higher in the Mg-N21 group compared to the MgO and Control groups (p < 0.05). Levels of brain antioxidant enzymes (CAT, GSH-Px, and SOD) and proteins involved in learning and memory (presynaptic synapsin I, PSD95, and PSD93) were higher in the HFD+Mg-N21 group compared to the other HFD groups (p < 0.05). Finally, in the HFD+Mg-N21 group, the number of platform crossings during the water maze (Figure 1) and time in target area were greater than the Control group (p < 0.05), while that number and time remained unchanged for the MgO group.

**Conclusions**

Results showed that Mg-N21 is a highly bioavailable form of magnesium that improves learning and memory compared to magnesium oxide. These results support the use of Mg-N21 as a well absorbed and retained form of magnesium to enhance cognition.

**Acknowledgements**

This study was conducted at Firat University and funded by Nutrition 21, LLC.

**Fig. 1 (abstract A52).**
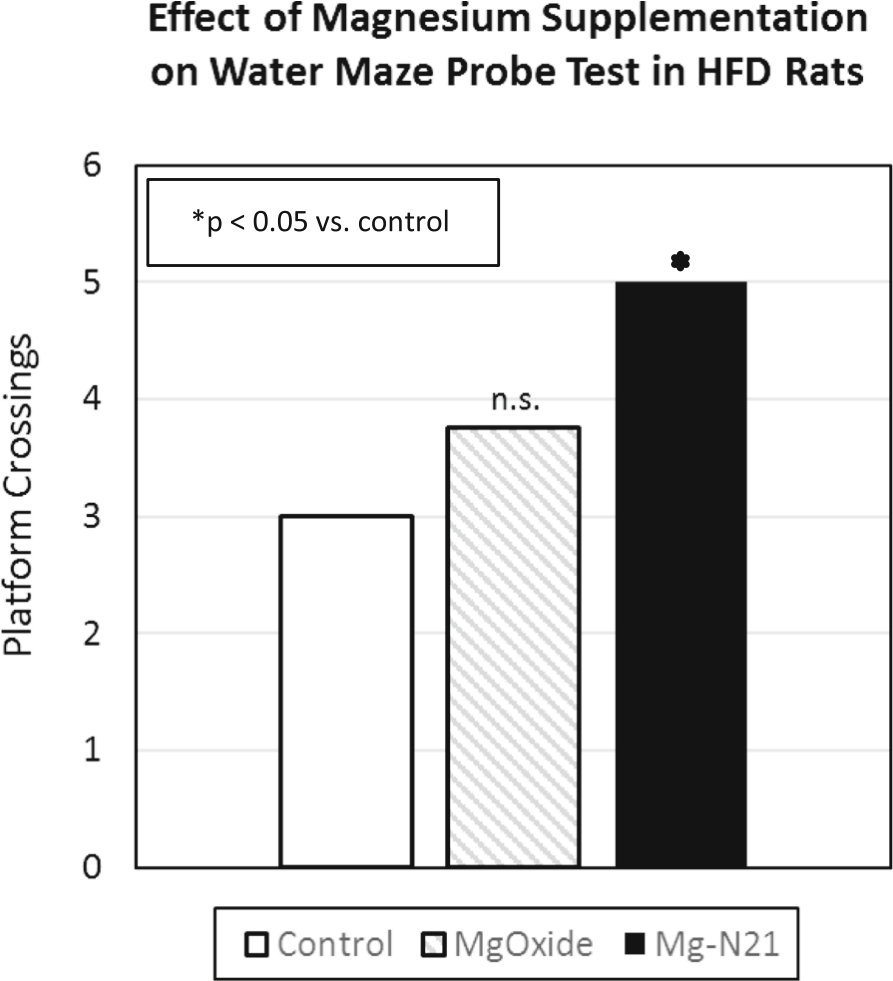
See text for description

## A53 The addition of an amylopectin/chromium complex to protein enhances the activity of muscle protein synthesis signaling factors

### James Komorowski^1^, Sara Perez Ojalvo^1^, Sarah Sylla^1^, Mehmet Tuzcu^2^, Cemal Orhan^2^, Kazim Sahin^2^

#### ^1^Nutrition 21, LLC, Purchase, NY 10577, USA; ^2^Firat University, Elazig, 23119, Turkey

##### **Correspondence:** James Komorowski (jkomorowski@nutrition21.com)

**Background**

An amylopectin/chromium complex (ACr; Velositol®) has been shown to significantly enhance muscle protein synthesis (MPS) when added to whey protein (WP) [1], branched chain amino acids (BCAAs), and pea protein. ACr is believed to have this effect by enhancing the mammalian target of rapamycin (*mTOR*) *signaling pathway*. This pathway is stimulated by insulin in the presence of adequate amino acids. Because chromium is known to enhance insulin sensitivity, ACr combined with protein may further enhance mTOR signaling via improved insulin action. To explore this hypothesis, the phosphorylation of mTOR signaling factors, such as mTOR, S6K1, and 4E-BP1, were analyzed in rat skeletal muscle following exercise and supplementation with protein and ACr.

**Materials and methods**

Young (8-week old) male Wistar rats weighing 250-300g were randomized into a control group or twelve different protein groups (n=8 per group) (Table 1). All rats completed a 10-day treadmill acclimation schedule and on the day of the single-dose experiment, rats were exercised at 26 m/min for 2 hours and then fed their assigned product immediately after exercise. Test products were dissolved in water and administered via oral gavage. One hour later, rats were sacrificed, and muscle tissue samples were taken to determine the phosphorylation of mTOR, S6K1, and 4E-BP1 via western blot methods.

**Results**

The addition of ACr to all forms of protein increased the activity of mTOR signaling factors compared to protein alone groups, over control. The addition of ACr to BCAAs increased mTOR phosphorylation by 64% (p < 0.05), S6K1 phosphorylation by 36% (p < 0.05), and 4E-BP1 by 23% (n.s). When added to pea protein, ACr enhanced the phosphorylation of mTOR by 68% (p < 0.05), S6K1 by 80% (p < 0.05), and 4E-BP1 by 28% (n.s). At all WP doses, ACr increased mTOR phosphorylation (p < 0.05), and at the 6g and 20g equivalent doses, ACr increased S6K1 phosphorylation (p < 0.05). At all WP doses, except for the 6 g WP equivalent group, ACr enhanced 4E-BP1 phosphorylation (p < 0.05).

**Conclusions**

When added to escalating doses of WP, as well as BCAAs and pea protein, ACr significantly increased the phosphorylation of MPS signaling proteins including mTOR, S6K1, and 4E-BP1. The results of this preclinical study confirm activity of the mTOR signaling pathway and its downstream targets through which ACr enhances MPS when added to various sources of protein.

**Acknowledgements**

This study was conducted at Firat University and funded by Nutrition 21, LLC.

**References**

1. Ziegenfuss TN, Lopez HL, Kedia A, Habowski, SM, Sandrock JE, Raub B, Kerksick CM, Ferrando AA: Effects of an amylopectin and chromium complex on the anabolic response to a suboptimal dose of whey protein. *JISSN* 2017, 4:6.

**Table 1 (abstract A53).** See text for description

	Treatment Group (Human Equivalent Dose)	Protein dose (g/kg BW)	Human protein dose equivalent (g)	ACr dose (g/kg BW)	Human ACr dose equivalent (g)
I	Exercise Control Group	0	0	0	0
II	(6g) Pea protein	0.456	6	0	0
III	(6g) Pea protein + ACr	0.456	6	0.155	2
IV	(6g) BCAA	0.456	6	0	0
V	(6g) BCAA + ACr	0.456	6	0.155	2
VI	(6g) Whey protein	0.456	6	0	0
VII	(20g) Whey protein	1.55	20	0	0
VIII	(30g) Whey protein	2.33	30	0	0
IX	(40g) Whey protein	3.10	40	0	0
X	(6g) Whey protein + ACr	0.465	6	0.155	2
XI	(20g) Whey protein + ACr	1.55	20	0.155	2
XII	(30g) Whey protein + ACr	2.33	30	0.155	2
XIII	(40g) Whey protein + ACr	3.10	40	0.155	2

## A54 An evaluation of the effects of inositol-stabilized arginine silicate (ASI; Nitrosigine®) on cognitive flexibility

### Douglas Kalman^1^, Susan Hewlings^2^, Sarah Sylla^3^, Sara Perez Ojalvo^3^, James Komorowski^3^

#### ^1^QPS-Miami, Miami, FL 33143, USA; ^2^Central Michigan University, Mt. Pleasant, MI 48859, USA; ^3^Nutrition 21, LLC, Purchase, NY 10577, USA

##### **Correspondence:** Douglas Kalman (douglas.kalman@qps.com)

**Background**

The Trail Making Test (TMT) is a widely-used instrument to assess cognitive processing speed and executive functioning. The test consists of two parts, A and B. Each test is measured by the time to completion, with lower scores indicating greater performance. TMT-A involves connecting an ascending sequence of 25 numbers, while TMT-B involves connecting an alternating sequence of 25 numbers and letters. While Part A of the TMT is a simpler test, Part B is a more complex test that requires the ability to mentally switch between tasks. Cognitive flexibility, or the mental ability to switch between concepts, is important for task-switching. Therefore, the difference between TMT-B and TMT-A scores, the TMT B-A score, has been established to emphasize the complexity of TMT-B and be a more direct measure of cognitive flexibility. A reduction in the TMT B-A score demonstrates higher cognitive flexibility, which is vital for performance in various sports that require constant shifting between cognitive tasks. Because ASI (inositol-stabilized arginine silicate; Nitrosigine**®**) has been shown to significantly improve TMT A and B scores individually compared to placebo [1], this post hoc analysis was carried out to examine the effects of ASI on the TMT B-A score.

**Materials and methods**

A randomized, crossover, double-blind, placebo-controlled trial was conducted to evaluate the acute effects of ASI (1,500 mg) on cognitive function in sixteen healthy, active male subjects (aged 18 to 35 years, BMI 19 to < 30 kg/m^2^) [1]. TMT B-A scores were calculated in each group before and after supplementation by subtracting TMT-A time from TMT-B time.

**Results**

After a single dose, the decrease in the mean TMT B-A score from baseline was significantly greater in the ASI group (-14.4 sec; -45%) compared to placebo (-1.5 sec; -4%) (Figure 1) (p < 0.05 between groups).

**Conclusions**

The results of this analysis show that ASI significantly improves TMT B-A scores, supporting the use of ASI to boost cognitive flexibility and improve athletic performance. Cognitive flexibility is vital for various performance activities, from video games to traditional sports such as soccer that require task switching and rapid reactions to various visual and auditory cues. Therefore, supplementation with ASI may heighten gamers’ and other athletes’ performance by enhancing cognitive flexibility after just a single dose.

**Acknowledgements**

This study was conducted at QPS-Miami and funded by Nutrition 21, LLC.

**References**

1. Kalman D, Harvey PD, Perez Ojalvo S, Komorowski J. Randomized prospective double-blind studies to evaluate the cognitive effects of inositol-stabilized arginine silicate in healthy physically active adults. *Nutrients* 2016, 8:736.

**Fig. 1 (abstract A54).**
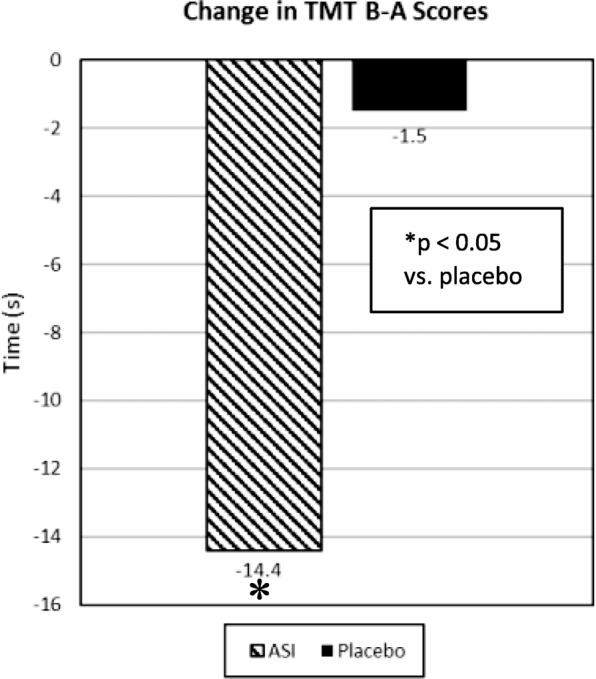
See text for description

## A55 The effect of a novel magnesium complex (Mg-N21) on metabolic function and body composition in high fat fed rats

### James Komorowski^1^, Sara Perez Ojalvo^1^, Sarah Sylla^1^, Cemal Orhan^2^, Mehmet Tuzcu^2^, Nurhan Sahin^2^, Kazim Sahin^2^

#### ^1^Nutrition 21, LLC, Purchase, NY 10577, USA; ^2^Firat University, Elazig, 23119, Turkey

##### **Correspondence:** James Komorowski (jkomorowski@nutrition21.com)

**Background**

Magnesium (Mg) is a mineral that acts as a co-factor for more than 300 enzymatic reactions in the body. It plays an important role in glucose metabolism and insulin sensitivity. Lower magnesium intake has been associated with increased risk for developing type 2 diabetes, metabolic syndrome, and insulin resistance. Supplementation with bioavailable forms of magnesium may be important for healthy metabolic function and body composition. This preclinical study was conducted to compare the effects of a novel form of magnesium, Mg-N21, to magnesium oxide (MgO) on metabolic function and body composition in rats.

**Materials and methods**

Forty-two male Wistar rats (age: 8-week, weight: 180 ± 20 g) were randomly divided into the following groups (n=7 per group): Control (no treatment), MgO, Mg-N21, HFD (fed with a high fat diet), HFD+MgO, and HFD+Mg-N21. Magnesium was dosed at 500 milligrams of elemental Mg/kg diet. All rats were supplemented for 8 weeks. At the end of treatment, plasma and tissue samples were obtained for analysis.

**Results**

Compared to the HFD group, visceral fat was 43% (-8.9 g) lower in the HFD+Mg-N21 group and 18% (-3.7 g) lower in the HFD+MgO group (p < 0.05 between groups) (Figure 1). While leptin levels were 10% lower in the HFD+MgO group compared to the HFD group, levels were 31% lower in the HFD+Mg-N21 group (p < 0.05 between groups). Glucose and insulin levels were lower in the HFD+Mg-N21 group compared to the HFD and HFD+MgO groups (p < 0.05 between groups). Moreover, glutamate receptor 1 and 2 levels were higher in the HFD+Mg-N21 group compared to the HFD and HFD+MgO groups (p < 0.05 between groups). Serum and liver levels of the oxidative stress marker MDA were reduced in the HFD+Mg-N21 group compared to the HFD and HFD+MgO groups (p < 0.05 between groups). No differences were observed between groups that were fed a normal diet.

**Conclusions**

The results of this study show that compared to MgO, Mg-N21 significantly improves indicators of healthy metabolic function, as well as body composition in rats fed a high fat diet. It is hypothesized that Mg-N21 may exert these effects by controlling hunger, improving glucose and insulin action, and inhibiting oxidative stress.

**Acknowledgements**

This study was conducted at Firat University and funded by Nutrition 21, LLC.

**Fig. 1 (abstract A55).**
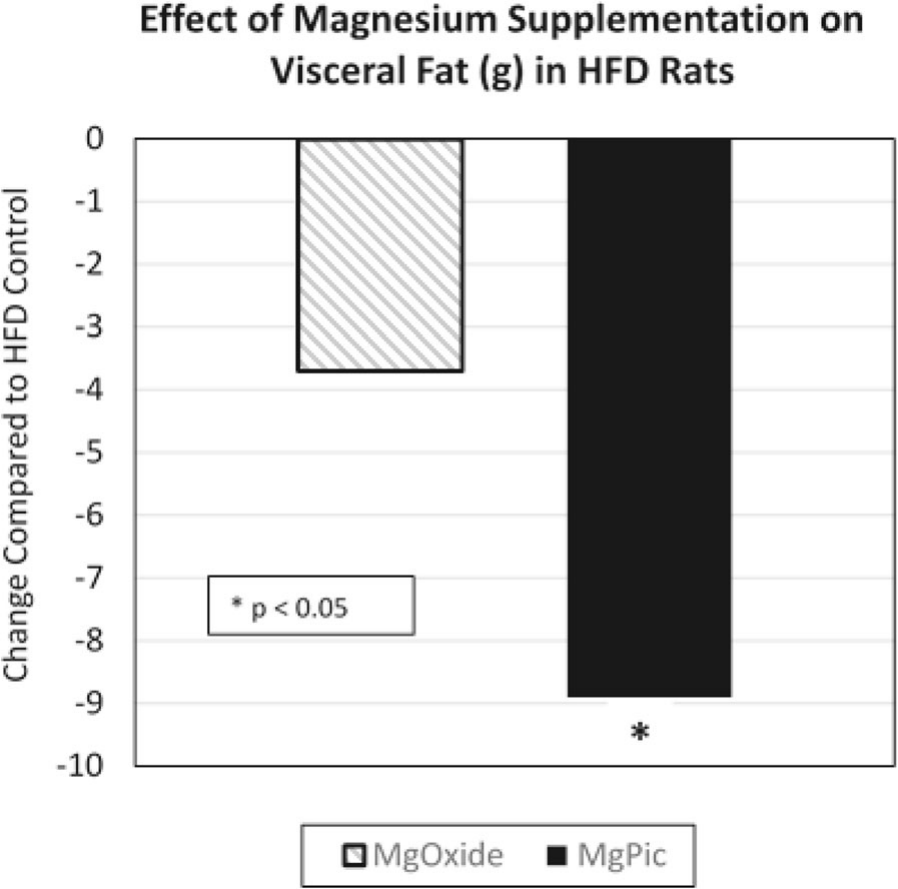
See text for description

## A56 Toward better reporting in sports nutrition: have we progressed?

### Jason M. Cholewa^1^, Brandon Roberts^2^, Christopher R. Harnish^3^, Jessica Kutz^3^, Neil Johannsen^4^, Conrad P. Earnest^5^

#### ^1^Department of Kinesiology, Coastal Carolina University, Conway, SC, 29526, USA; ^2^UAB Center for Exercise Medicine, University of Alabama at Birmingham, Birmingham, AL, 35487, USA; ^3^Department of Exercise Science, Shenandoah University, Shenandoah, VA, 22601, USA; ^4^School of Kinesiology, Louisiana State University, Baton Rouge, LA 70803, USA; ^5^Exercise and Sports Nutrition Laboratory, Texas A&M University, Galveston, TX, 77554, USA

##### **Correspondence:** Conrad P. Earnest (cearnest@tamu.edu)

**Objective**

The interpretation of research studies involving a treatment is dependent upon a robust trial design and the accurate reporting of subsequent findings. An essential component to interpreting the research findings of any study is the presentation of a scientific report in respective journals. On an intuitive level, consistency across journals relative to reporting standards should be the norm. However, we contend that a misunderstanding and lack of consistency regarding data reporting in intervention trials involving sports nutrition still exists. Therefore, the aim of our study was to examine the robustness of publications reporting intervention trials in sports nutrition relative to The Consolidated Standards of Reporting Trials (CONSORT) recommendations.

**Methods**

We examined 236 papers from ten journals published in 2016. The primary outcome was statistical variance associated with treatment (correct SD vs. SEM or CI). Secondary outcomes included reported: (a) effect sizes (Y/N), (b) outcome prioritization (Y/N; primary, secondary etc.) and (c) statistical variance relative to change from baseline expressed CI (recommended CI vs. SD or SEM) and (d) directed hypotheses (specific, non-specific, none), and an exploratory analysis for journal prevalence regarding each factor. Prevalence was examined using chi-square analyses and between pairwise between category comparisons were Bonferonni adjusted for multiple comparisons.

**Results**

Overall, we observed significant trends for all analyses (*all*, p<0.001). For our primary outcome, 128 (59%) articles correctly used SD to denote treatment variance, while 79 (36%) and 11 (5%) articles used SEM and CI, respectively. For secondary outcomes, 63 articles (29%) reported effect sizes and 155 (71%) did not. 188 articles (86%) failed to prioritize outcomes, 134 articles (61%) stated no hypotheses and 40 (19%, out of 100) articles correctly used CI to denote change from baseline, while SD (19%, n=41), SEM (n=10, 5%) no variance term (n=8, 4%) were incorrectly used. Between category comparisons were all significant (p<0.002).

**Conclusions**

Journals reporting on sports nutrition fail to adhere to more robust CONSORT based recommendations. We suggest that the initial burden falls on publishers. However, Editors-in-Chief and peer reviewers are equally responsible for enhancing data reporting standards. Despite the evolution of advanced measurement techniques in science, it is apparent that reporting standards still lag behind. The time has come for the sports sciences to raise the bar of scientific reporting.

## A57 The effects of a multi-ingredient preworkout supplement vs. caffeine on strength-based performance: a double-blind, randomized, placebo controlled trial

### Haley McNulty, Jason M. Cholewa

#### Department of Kinesiology, Coastal Carolina University, Conway, SC, 29528, USA

##### **Correspondence:** Jason M. Cholewa (jcholewa@coastal.edu)

**Background**

Multi-ingredient pre-workout supplements (PWOS) have been shown to improve various metrics of muscular performance during resistance exercise. Caffeine, the main ingredient in most PWOS, appears to be largely responsible for these ergogenic effects, with little research examining whether the ancillary ingredients (i.e.: taurine, agmatine sulfate, Teacrine, etc.) confer additional benefits. To our knowledge, nearly all PWOS studies have been compared against a placebo, and never directly against caffeine. Therefore, the purpose of this pilot study was to conmpare the effects of a PWOS against a caffeine matched condition.

**Methods**

Men (*n*=5; 22.4±3.8 years, 178.1±6.4 cm, 84.7± 10.8 kg, 15.5±5.9 BF%) with at least one year of resistance training were randomly assigned a placebo (P), caffeine (C: 250 mg), or a PWOS (Perform Kinetics, ATP Nutrition, Tempe, AZ) treatment 30 min prior to each session. Testing was separated by one week and consisted of the following: vertical jump, medicine ball chest pass, and one set of back squat and bench press to failure with 70% 1RM. Subjects were instructed to perform squat and bench press repetitions at maximal velocity. A linear transducer was used to measure mean power during the squat and bench press, and quality repetitions were recorded as repetitions performed at or above 90% mean power. Statistical analyses were performed using separate repeated measures ANOVAs with an alpha level of *p* ≤ .05.

**Results**

There were no significant differences between conditions for vertical jump (P: 76.0±7.0; C: 75.2±6.3.; PWOS: 74.5±5.6 cm), chest pass velocity (P: 5.6± 1.4; C: 6.1± 0.9; PWOS: 5.9±1.6 m/s), squat repetitions (P: 16±2.6; C: 17±5.4; PWOS: 17.8±4.3), bench press repetitions (P: 13.6±3.0; C: 14.0±3.4; PWOS: 14.0±3.3), or bench press quality repetitions (P: 2.6±1.8; C 3.8±1.3; PWOS 4.2±2.3). Significant differences were found between groups for squat quality repetitions: more quality repetitions were performed during the PWOS condition (5.2±1.3) than the caffeine (2.6±2.2) or placebo (3.8±1.3) condition.

**Conclusions**

The preliminary results of this study do not seem to suggest a strong synergistic effect between the ingredients in a PWOS. However, these results should be interpreted with caution, as the current sample size is small and when compared to placebo, a large (*d*=0.875) effect size was found for quality squat repetitions in the PWOS condition. Our preliminary results do not discount the possibility that a PWOS may be more ergogenic than a matched dose of caffeine, but rather support the need for ongoing data collection.

**Acknowledgements**

ATP Nutrition provided the supplements for this study.

## A58 The effect of 6 weeks of static stretching and foam rolling on ankle dorsiflexion and range of motion

### Sydney Brown, Mary Francis Aini, MacGregor Hall, Brandi Washell, Jason Chad Smith

#### Department of Kinesiology, Coastal Carolina University, Conway, SC, 29528, USA

##### **Correspondence:** Jason Chad Smith (Jsmith6@coastal.edu)

**Background**

Foam rolling is used by athletes and the general population to improve range of motion (ROM) and ease pain associated with delayed onset muscle soreness. While several studies have documented acute increases in ROM using foam rolling, only one chronic study has been conducted, with similar increases between foam rolling and PNF stretching. The acute and chronic effects of static stretching on ROM have been studied extensively, however, traditional static stretching and foam rolling have not yet been directly compared. Therefore, the purpose of this investigation was to evaluate the acute and chronic effects of 6 weeks of static stretching and foam rolling, separately and combined, on ankle dorsiflexion ROM.

**Materials and methods**

Forty-one participants completed two sessions per week for a total of six weeks, with each session separated by at least 48 hours. Subjects were randomly assigned to one of three training groups (Static Stretching (n = 16), Foam Rolling (n = 14), and a combination of both (n = 11). A metronome was used to control speed of the foam rolling. After thirty seconds of foam rolling, the participants switched leg positions and foam rolled the opposite leg. A total of three sets of 30 seconds of foam rolling was performed on each leg. Three dorsi-flexion ROM measurements were taken on the dominant leg, both before and following the first session (acute) and last session (chronic) with the largest measurement recorded for analysis. Repeated measures ANOVA with a between-subjects effect was used to assess statistical differences across time in ROM. Paired t-tests with a Bonferroni adjustment was used as a post-hoc analysis. The alpha level was set to 0.05.

**Results**

There were no differences in ROM acutely. ROM significantly increased chronically (*p*<.001). In the present study, we observed a 3.5%, a 4.7% and a 4.3% acute increase in ROM for static stretching, foam rolling, and foam rolling combined with static stretching, respectively, after training; however, there were no differences between groups.

**Conclusions**

Foam rolling appears to be just as effective as static stretching in terms of increasing ROM in the healthy, young adult population after 6 weeks of training. Given that the neurological effects of static stretching and foam rolling are transient, and adaptations in this study may have been dissipated following 4 days of recovery between weekly sessions, future investigations should study the effects of more frequent foam rolling on ROM.

## A59 Beta-Alanine does not influence the hypertrophic outcomes associated with blood flow restriction or conventional upper body resistance training

### Kassiana Araújo Pessôa^1,2^, Fernanda Lima-Soares1^1,2^, Xia Zhi5^3,4^, Alessandro Moura Zagatto^5^, Antonio Herbert Lancha-Jr^6^, Jason Cholewa^7^, Fabrício Eduardo Rossi^8^, Nelo Eidy Zanchi^1,2^

#### ^1^Department of Physical Education, Federal University of Maranhão, São Luís, 06140-052, Brazil; ^2^Laboratory of Cellular and Molecular Biology of Skeletal Muscle (LABCEMME), São Luís, 06140-052, Brazil; ^3^Exercise Physiology and Biochemistry Laboratory, College of Physical Education, Jinggangshan University, Ji'an, 331300, China; ^4^Department of Sports Medicine, Chengdu Sport University, Chengdu, 610000, China; ^5^São Paulo State University (UNESP), Department of Physical Education, Bauru, 17010, Brazil; ^6^Laboratory of Applied Nutrition and Metabolism, School of Physical Education and Sports, University of Sao Paulo, Sao Paulo, 14780, Brazil; ^7^Department of Kinesiology, Coastal Carolina University, Conway, SC, 29528, USA; ^8^Immunometabolism of Skeletal Muscle and Exercise Research Group, Department of Physical Education, Federal University of Piauí (UFPI), Teresina-PI, 64000, Brazil

##### **Correspondence:** Nelo Eidy Zanchi (nelo@ig.com.br)

**Introduction**

Chronic beta-alanine (BA) supplementation has been shown to increase the intramuscular H^+^ buffer carnosine, and several studies suggest BA enhances the muscular performance and hypertrophy outcomes associated with resistance training. Blood flow restriction resistance training (BFR) relies on glycolytic metabolism, and has also been shown to promote hypertrophic outcomes, potentially through the generation of metabolic byproducts (i.e.: lactate, H^+^ ions). Theoretically, a greater glycolytic flux during BFR as a result of elevated intramuscular carnosine should result in greater mechanical work and metabolic byproducts, thereby accentuating hypertrophy following BFR training. Therefore, the purpose of this study was to investigate the interaction between BA supplementation and BFR training.

**Methods**

Untrained male subjects (*n*=20, 22.2±2.9 years, 174.5±0.5 cm, 70.0±8.2 kg) were randomly divided into a placebo or BA (4.8 g/day) group in a double-blind fashion. Subjects trained each arm with either a BFR (3-4 sets of 15-30 repetitions at 30% 1RM) or traditional (3-4 sets of 10-12 repetitions at 70% 1RM) arm flexion protocol on separate days twice weekly for 6 weeks. Protocol assignment was done randomly to ensure an equal distribution between dominant and non-dominant arms between protocols. Arm flexion 1RM and muscle thickness (B-mode ultrasound) were measured pre- and post-trial. A mixed factorial ANOVA with repeated measures was used to compare within subjects (BFR vs. traditional), between groups (BA vs. placebo), across time (pre- vs. post-testing), and to assess for any conditions x group x time interactions with an alpha of *p* ≤ .05.

**Results**

There were no significant differences between groups or conditions at baseline. 1RM increased across time and significantly (*p*<.001) greater increases in 1RM were found for traditional compared to BFR training with no differences between supplement groups. Muscle thickness also increased across time, with significantly (*p*=.003) greater increases in the BFR compared to traditional protocols (9.0±6.4 vs. 5.3±4.4 %), but again without differences between supplement groups.

**Conclusions**

The main findings of this study were that arm flexion BFR training induced significantly greater hypertrophic outcomes compared to traditional resistance training, and BA supplementation did not accentuate these results in either training condition. We were unable to measure lactate or blood pH, and therefore future research is necessary to investigate how BA effects the metabolic response to BFR training. Future research using this unilateral design should also investigate the interaction between BA and BFR training in the larger, lower body musculature.

## A60 Perceptions of energy and macronutrient intake in a group of collegiate female lacrosse athletes

### Chad M. Kerksick, Hannah Zabriskie, Patrick S. Harty, Richard Stecker, Andrew R. Jagim

#### Exercise & Performance Nutrition Laboratory, Lindenwood University, St. Charles, MO 63301, USA

##### **Correspondence:** Chad M. Kerksick (ckerksick@lindenwood.edu)

**Background**

The purpose of this study was two-fold: 1) to identify the discrepancies that exist in female collegiate athletes between their perceived energy and macronutrient needs and their perceived intake, and 2) to identify the differences between perceived intake and actual intake of energy and macronutrients.

**Materials and methods**

Eighteen female NCAA Division II lacrosse players (169.5 ± 6.3 cm; 68.9 ± 10.3 kg; 26.7 ± 3.1 % fat) completed a 4-day monitoring period during in-season competition. Over four consecutive days, athletes were outfitted with a combined heart rate and activity monitor (Acti-Heart, CamNTech) to assess total daily energy expenditure (TDEE) and completed four-day food and fluid records to assess dietary intake. Dietary intake was self-reported using a commercially available food tracking program (*MyFitnessPal©, USA*). Daily average values were calculated for total and relative energy, protein, carbohydrate, and fat intake. In addition, each athlete recorded their perceived nutrient needs and their perceived nutrient intake over the collection period. Due to widespread lack of normality found within the perceived data, Wilcoxon Signed Rank tests were used to assess differences between perceived needs and perceived intake as well as between perceived intake and actual intake.

**Results**

As seen in Table 1, widespread variability existed within the perceived needs and intake data resulting in large deviations from normality. Significant differences between the perceived needs and perceived intakes were for absolute CHO, relative CHO, absolute fat, and relative fat while significant differences between perceived intake and actual intake were found for relative protein, absolute fat, and relative fat. All perceived needs, perceived intake and actual dietary intake data is provided in Table 1.

**Conclusions**

When examining athletes’ perceptions of their energy and macronutrient needs as well as their perceived intake against their actual intake, widespread discrepancies are found. In particular, perceptions associated with how much dietary carbohydrate and fat is needed and how much is consumed displayed massive standard deviations and impressive ranges suggesting that some athletes lack even a basic understanding of their daily needs. Moreover, female lacrosse athletes perceived their needs for protein to be less than what they are actually consuming. On a positive note, athletes seem to have a better grasp of the amount of calories needed in their diet. Results from this data suggest that collegiate athletes lack appropriate understanding of basic nutrition needs and could benefit from basic nutrition education as it pertains to their health and performance.

**Table 1 (abstract A60).** Perceived needs, perceived intake and actual intake

n=18	Perceived Needs (MIN, MAX)	Perceived Intake (MIN, MAX)	Delta Perceived p-value*	Actual Intake	Delta Intake p-value*
Absolute Energy Intake (kcal/d)	2211 ± 617 (1200, 4000)	2314 ± 782 (1250, 4000)	-102.8 ± 530 *0.690*	2,135 ± 405	178.4 ± 742 *0.306*
Relative Energy Intake (kcal/kg/d)	31.2 ± 7.8 (18.6, 61.9)	34.4 ± 12.8 (17.1, 61.9)	-1.50 ± 7.8 *0.557*	31.7 ± 8.1	2.66 ± 11.0 *0.327*
Absolute CHO (g/d)	185.1 ± 247 (10, 800)	392 ± 710 (15, 3000)	-207 ± 685 *0.002*	243 ± 71	149.3 ± 667 *0.616*
Relative CHO (g/kg/d)	2.94 ± 4.1 (0.15, 13.0)	5.9 ± 10.1 (0.19, 41.9)	-2.96 ± 9.6 *0.003*	3.6 ± 1.16	2.29 ± 9.5 *0.679*
Absolute PRO (g/d)	67.6 ± 92.9 (15, 400)	72.8 ± 125 (5, 500)	-5.14 ± 36.1 *0.304*	77.7 ± 19.8	-4.93 ± 128.4 *0.028*
Relative PRO (g/kg/d)	1.06 ± 1.55 (0.2, 6.5)	1.16 ± 2.1 (0.08, 8.1)	-0.10 ± 0.60 *0.360*	1.17 ± 0.40	-0.007 ± 2.1 *0.028*
Absolute FAT (g/d)	60.3 ± 111.9 (7, 500)	(140.5 ± 296 (10, 1200)	-80.3 ± 273 *0.007*	88.0 ± 23.2	52.4 ± 299 *0.035*
Relative FAT (g/kg/d)	0.94 ± 1.82 (0.07, 8.1)	2.1 ± 4.3 (0.14, 16.8)	-1.15 ± 3.8 *0.008*	1.31 0.44	0.78 ± 4.3 *0.035*

Data presented as mean ± SD. Ranges of perceived needs and perceived intakes are provided in parentheses. MIN = Minimum value. MAX = Maximum value. Delta perceived = Perceived needs – perceived intake. Actual intake = Perceived intake – actual intake. * = p-value from Wilcoxon Signed Rank Test.

## A61 The relationship between absolute and relative lean mass with cardiometabolic outcomes

### Katie R. Hirsch^1,2^, Kara C. Anderson^1^, Alexis A. Pihoker^1^, Meredith G. Mock^1^, Malia M.N. Blue^1,2^, Austin M. Peterjohn^1^, Eric T. Trexler^1,2^, Abbie E. Smith-Ryan^1,2^

#### ^1^Applied Physiology Laboratory, Department of Exercise and Sport Science, University of North Carolina, Chapel Hill, NC, 28804, USA; ^2^Human Movement Science Curriculum, Department of Allied Health Science, University of North Carolina, Chapel Hill, NC, 28804, USA

##### **Correspondence:** Abbie E. Smith-Ryan (abbsmith@email.unc.edu)

**Background**

Lean mass (LM) plays an important role in health, functionality, and quality of life. Although greater LM is often considered advantageous, absolute LM is highly influenced by other factors such as weight, height, and fat mass (FM). The purpose of this study was to evaluate the relationship between different indices of LM with cardiometabolic outcomes.

**Materials and methods**

A total of 174 individuals (Males=52; Females=122; Mean ± SD [Range]: Age: 26.2 ± 9.2 yrs [18-54 yrs]; BMI: 25.6 ± 6.0 kg·m^-2^ [18.5-52.0 kg·m^-2^]) were evaluated. Absolute FM (kg) and LM (kg) were measured using dual energy x-ray absorptiometry. To account for weight, height, and FM, percent LM (%LM=[LM/body mass (kg)]*100), lean mass index (LMI=LM·m^-2^), and LM to FM ratio (LM:FM) were calculated, respectively. Total cholesterol (TC), high-density lipoproteins (HDL), non-high-density lipoproteins (non-HDL), TC to HDL ratio (TC/HDL), and fasting glucose (GLUC) were evaluated from a blood sample. Relationships between indices of LM and cardiometabolic outcomes were evaluated with Pearson correlations.

**Results**

In the full sample, greater absolute LM was significantly associated (p<0.05) with less favorable cardiometabolic outcomes [HDL (R= -0.415); non-HDL (R=0.312); TC/HDL (R=0.434); TC and GLUC (p>0.05)]. A higher LMI was also significantly associated (p<0.05) with less favorable cardiometabolic outcomes [TC (R=0.209), non-HDL (R=0.395), TC/HDL (R=0.494), HDL (R= -0.456); GLUC (p=0.223)]. In contrast, a greater %LM and LM:FM was significantly associated (p<0.05) with more favorable cardiometabolic outcomes [TC (R= -0.417 and -0.396), non-HDL (R= -0.388 and -0.348), TC/HDL (R= -0.250 and -0.214); HDL and GLUC (p>0.05)]. Results were consistent when stratified by sex, with the exception of absolute LM in females was not associated with any cardiometabolic outcomes (p>0.05). In females, there was also a weak positive association among %LM and LM:FM ratio with GLUC (R=0.201 and R=0.199; p<0.05).

**Conclusion**

The association between LM and cardiometabolic risk factors is highly dependent on the index used. Greater absolute LM and LMI were associated with less favorable cardiometabolic outcomes, which likely reflects the influence of FM and may be better indicators of stature. When corrected for weight, relative indices of LM (%LM and LM:FM), demonstrated a more favorable association with cardiometabolic outcomes and may be more insightful for evaluating LM when accounting for FM. Differences in associations observed in males and females require further investigation.

## A62 The effects of TeaCrine®, caffeine, or a combination of both on muscular strength and endurance in resistance-trained men

### Gustavo A. Ramos, Kyle C. Cesareo, Patrick G. Saracino, Justin R. Mason, Margaret C. Morrissey, Michael J. Ormsbee

#### Institute of Sport Sciences & Medicine, Department of Nutrition, Food & Exercise Sciences, Florida State University, Tallahassee, FL, 32306, USA

##### **Correspondence:** Michael J. Ormsbee (mormsbee@fsu.edu)

**Background**

TeaCrine® is the synthetic version of naturally occurring theacrine (1, 3, 7, 9-tetramethyluric acid) found in the leaves of the *Camellia kucha* tea plants. Data exist on the effects of TeaCrine® on cognitive function, but no research exists examining its effects on exercise performance. The purpose of this study was to determine the efficacy of TeaCrine® on muscular strength and muscular endurance performance in resistance-trained men compared to caffeine, a combination of Teacrine® + caffeine, and a placebo.

**Materials and methods**

Twelve resistance trained men (age: 23 ± 3 years; height: 176.5 ± 5.9 cm, weight: 83.2 ± 7.2 kg) participated in this study. Each participant performed (in order) one repetition maximum (1RM) bench press, 1RM squat, bench press repetitions (reps) to failure (RTF) at 70% 1RM, squat RTF at 70% 1RM, and 2-km rowing time trial after the consumption of (in random order): (1) Caffeine 300 mg (CAF300); (2) TeaCrine® 300 mg (TEA300); (3) TeaCrine® + Caffeine combo (150 mg/150 mg) (COMBO); (4) Placebo 300 mg (PLA). Power and velocity were measured using a TENDO power analyzer. Visual analog scales for energy, focus, motivation to exercise, and fatigue were administered at baseline and 90 min post-treatment ingestion. Rating of perceived exertion was assessed after bench press and squat RTF. Magnitude-based inferences were utilized to examine performance effects in 1RM and RTF for bench press and squat. Performance and perceptual data were assessed using null hypothesis testing using SPSS. Data are presented as mean ± SD (ES: effect size).

**Results**

There were no differences in 1RM bench press (CAF300: 120 ± 16 kg (0.14), TEA300: 119 ± 16 kg (0.08), COMBO: 120 ± 16 kg (0.14), or PLA: 117 ± 16 kg) or squat (CAF300: 151 ± 24 kg (0.12), TEA300: 148 ± 25 kg (0.01), COMBO (150 ± 24 kg (0.09), PLA: 148 ± 21 kg) vs. PLA. There was no difference in RTF for bench press (CAF300: 12 ± 3 reps (0.05), TEA300: 13 ± 3 reps (0.02), COMBO: 13 ± 3 reps (0.06), PLA: 12 ± 3 reps) or squat (CAF300: 13 ± 3 reps (0.28), TEA300: 11 ± 3 reps (0.02), COMBO: 12 ± 4 reps (0.06), PLA: (11 ± 4 reps) vs. PLA.

There were no significant differences in peak or average power or velocity for bench press or squat between groups. Only CAF300 resulted in significant increases in perceived energy and motivation to exercise vs. TEA300 and PLA (Energy: +9.8%, +15.3%; Motivation to exercise: +8.9%, +14.6%, respectively) and increased focus (+9.6%) vs. TEA300, but there were no differences between CAF300 and COMBO.

**Conclusion**

In resistance-trained men, CAFF300, TEA300 and COMBO had no significant effect on 1RM, RTF, power or velocity in the bench press and squat. CAFF300 improved focus, energy and motivation to exercise while TEA300, COMBO, and PLA did not.

**Acknowledgements**

Compound Solutions, Inc. grant

## A63 Bone mineral density in NCAA Division I female athletes

### Malia N. M. Blue^1,2^, Katie R. Hirsch^1,2^, Eric T. Trexler^1,2^, Kara C. Anderson^1^, Alexis A. Pihoker^1^, Austin M. Peterjohn^1^, Abbie E. Smith-Ryan^1,2^

#### ^1^Applied Physiology Laboratory, Department of Exercise and Sport Science, University of North Carolina, Ashville, NC, 28804, USA; ^2^ Human Movement Science Curriculum, Department of Allied Health Science, University of North Carolina, Ashville, NC, 28804, USA

##### **Correspondence:** Abbie E. Smith-Ryan (abbsmith@email.unc.edu)

**Background**

Bone mineral density (BMD) may indicate an athlete’s risk of bone injury or predisposition to osteopenia and/or osteoporosis, which is especially important for females. Assessing sport-specific differences in BMD may provide insight about risk for bone-related injury, leading to more targeted nutritional and resistance training interventions. The purpose of the current study was to evaluate sport-specific BMD and the prevalence of low BMD in Division I female athletes. Secondary analyses investigated the association of BMD with body composition.

**Materials and methods**

One-hundred and twenty-five female Division I athletes (Mean ± SD; Age: 19.2 ± 1.2 yrs, Weight: 59.8 ± 7.7 kg, %Fat: 22.6 ± 3.3 %) were evaluated between the years 2013-2017. Dual-energy X-ray absorptiometry was used to measure BMD, body fat percentage (BF%), and fat free mass index (FFMI). Participants were classified by sport: Gymnastics (GYM; n=37), Cross Country (XC, n=38), Track (n=22), and Swimming and Diving (SWIM; n=28). An analysis of variance evaluated differences between sports. Pearson correlation coefficients assessed the relationship of BMD, FFMI, and BF%.

**Results**

The percentage of all female athletes below the NHANES 50^th^ percentile for BMD was 27.2%. The prevalence for SWIM was 60.1%, XC= 42.1%, Track= 4.5%, and no gymnasts were below the 50^th^ percentile. There was no significant difference in BMD (p=0.102) between GYM (1.271±0.016 g/cm^2^) and Track (1.211±0.021 g/cm^2^), and no significant difference (p=0.162) between SWIM (1.075±0.18 g/cm^2^) and XC (1.125±0.16 g/cm^2^). BMD was significantly lower in SWIM than GYM (p<0.001) and Track (p<0.001), and significantly lower in XC than GYM (p<0.001) and Track (p=0.006). For all athletes, BMD was positively correlated to FFMI (R=0.430, p<0.001), and not significantly correlated to BF% (p=0.904). FFMI and BF% were not significantly correlated (p=0.952).

**Conclusions**

Swimmers and XC runners had the highest prevalence of low BMD and had significantly lower BMD compared to gymnasts and track athletes. BMD was positively associated with FFMI and not associated with BF%, therefore FFMI may be useful to establish body composition goals as opposed to BF% alone. Athletic support staff should focus on nutritional and resistance training interventions to increase BMD for female swimming and XC athletes. Future investigations should evaluate changes in BMD following interventions in at-risk female athletes.

## **A64 Effect of sensoril**® **on strength training adaptations, body composition, muscular performance, and recovery: the star trial**

### Tim N. Ziegenfuss, Scott Habowski, Jennifer E. Sandrock, Betsy Raub, Hector L. Lopez

#### ^1^The Center for Applied Health Sciences, 4300 Allen Road, STE 120/130, Stow, Ohio 44224, USA

##### **Correspondence:** Tim N. Ziegenfuss (TZ@appliedhealthsciences.org)

**Background**

Withania somnifera (Ashwagandha) is an Ayurvedic herb categorized as having “rasayana” (rejuvenator), longevity, and revitalizing properties. Sensoril® is a standardized aqueous extract of the roots and leaves of Withania somnifera. The purpose of this study was to compare the effects of supplementation with Sensoril® vs. placebo on strength training adaptations, body composition, muscular performance, and recovery.

**Materials and methods**

Using a randomized, double-blind design, 66 recreationally active men (mean ± SD age, height, weight, % body fat: 26.3 ± 6.7 y, 180 ± 6.7 cm, 87.0 ± 12.8 kg, 24.3 ± 6.5 %) were matched for training experience, body weight, and strength prior to being randomized into 1 of 3 groups: placebo (P), 250 mg/d Sensoril^**®**^ (S250) or 500 mg/d Sensoril^**®**^ (S500). Body composition (DEXA), muscular performance (upper/lower body strength [1-RM bench/squat], power [Tendo], repetitions to failure [RTF: 3 sets x 65% max, 60-sec rest between sets]), 7.5 km time trial, perceived recovery (VAS) and clinical blood chemistries were measured at baseline and after 12 weeks of supplementation and training. Subjects were required to maintain their normal dietary habits and follow a specific, progressive overload resistance-training program (4-d/wk, upper body/lower body split). Data were analyzed via ANOVA/ANCOVA and statistical significance was set *a priori* at p≤0.05.

**Results**

Gains in 1-RM squat were significantly greater (p<0.03) in S500 (+19.1 ± 13.0 kg [18.2% incr]) vs. S250 (+13.3 ± 9.5 kg [14.5% incr]) and vs. P (+10.0 ± 6.2 kg [9.6% incr]). Significant within-group improvements for S500 in peak power bench (11.3%, p<0.007), peak power squat (8.5%, p<0.001), 7.5 km time trial (21.3%, p<0.001), RTF bench (28.2%, p<0.001), and VAS (14.4%, p<0.003) were greater than S250 and P. No statistically significant differences in body composition or systemic hemodynamics (heart rate, systolic and diastolic blood pressures) were noted, and aside from a slight polycythemia effect in the P group, blood chemistries (glucose, BUN-to-creatinine ratio, sodium, potassium, serum protein, albumin-to-globulin ratio, bilirubin, alkaline phosphatase, alanine aminotransferase, aspartate aminotransferase, total cholesterol, HDL-cholesterol, triacylglycerol, LDL-cholesterol, CBC) remained within normal clinical limits.

**Conclusions**

These preliminary data indicate that S500 administration augments performance adaptations to training in recreationally active men. Future studies are needed to confirm and clarify these results.

**Acknowledgements**

Supported in part by a research grant from Natreon, Inc

## A65 The acute effects of rugby competition on cognitive function- a pilot study

### Troy Purdom, Stephen Cain, Josh Morgan, Lindsey Byers, L.

#### Department of Health, Athletic Training, Recreation, and Kinesiology, Longwood University, Farmville, VA, 23909, USA

##### **Correspondence:** Troy Purdom (purdomtm@longwood.edu)

**Background**

The nature of rugby competition inherently stresses the body and brain through long duration and high intensity exercise, which has been shown to induce temporary hypoxemia [1]. Furthermore, rugby is a game fraught with repetitive minor head collisions [2]. Reduced oxygen availability [3] and acute head trauma [2] have been shown to affect critical thinking and decision making. Therefore, the aim of this study is to investigate if rugby effects cognition immediately after a collegiate club rugby match.

**Materials and methods**

Twelve male club rugby players (mean ± SD: 20.8 ± 1.1yrs, 181.0 ± 6.9cm, 90.0 ± 11.1kg, 322.5 ± 96.7min slept, 46.7 ± 15.0min played) were used to evaluate the effect of a rugby match on critical thinking and decision making. Prior to testing, all participants attended a familiarization session to obtain informed consent and to introduce the Stroop Test (ST) electronic application and testing protocol. Prior to all testing, demographic information was measured. Pre-test guidelines asked that participants refrain from alcohol, nicotine (tobacco or vaping), caffeine and other stimulants/depressants for a minimum of 24 hours prior to the competition. Participants completed the ST with an electronic device on the field prior to a club rugby match and then again immediately after exiting the game. Exclusion criteria consisted of players who violated pre-test guidelines, testing protocol, and/or played <30min of the match. Paired t-tests were used to compare the pre/post ST results. Data are presented as mean ± SD.

**Results**

Paired t-tests revealed a significant reduction (*p* < 0.005) in pre and post ST indicating a decrement in cognitive function (PRE: 20.6 ± 9.4; POST: 14.8 ± 6.9). Participants that played ≥30min experienced a 16.4% decrease in cognitive function.

**Conclusion**

Results show that rugby competition negatively impacted cognitive function in collegiate club rugby athletes. The high intensity [1] and sustained duration of rugby competition can perpetuate a repetitive hypoxemic state in addition to the subconcussive head trauma experienced regularly by rugby athletes [2], which can negatively affect cognition. Further research is warranted to better understand the negative effects the sport of rugby has on hierarchal brain function and how to better protect athletes during high risk athletic competitions.

**Acknowledgements**

A special thank you to the Longwood University Men’s Club Rugby Team.

**References**

1. Dempsey, JA. Wagner, PD. Exercise-induced arterial hypoxemia. Journal of Applied Physiology. 1999; 87(6): 1997-2006.

2. Johnson, B. Neuberger, T. Gay, M. Hallett, M. Slobounov, S. Effects of subconcussive head trauma on the default mode network of the brain. Journal of Neurotrauma. 2014; 31(23): 1907-1913.

3. Asmaro, D. Mayall, J. Ferguson, S. Cognition at altitude: impairment in executive and memory processes under hypoxic conditions. Aviation, Space, and Environmental Medicine. 2013; 84(11): 1159-1165.

## A66 A double-blind, placebo-controlled crossover study of low dose creatine on cognitive function before and after high intensity exercise

### Jacob Giles, Tristin Wind, Carter Leake, Samuel Yardley, Kyle Levers, Troy Purdom

#### Department of Health, Athletic Training, Recreation, and Kinesiology, Longwood University, Farmville, VA, 23909, USA

##### **Correspondence:** Troy Purdom (purdomtm@longwood.edu)

**Background**

To observe the effect of low-dose creatine supplementation and high-intensity exercise on cognitive function in recreationally trained college aged males.

**Materials and methods**

Thirteen recreationally trained males (mean±SD: 20.9±1.3yrs, 79.4±9.9kg, 175.2±6.6cm, 16.4±6.2%BF) were used to evaluate low dose creatine on cognitive function. Cognition was measured using the Stroop Test (ST) where participants had one-minute to correctly select color-word combinations (i.e.: if the word “green” was written in red, the correct answer would be red. Prior to all procedures, participants attended a familiarization session to provide informed consent and acclimate to the ST. Demographic information was measured prior to completing the control (CONT) where ST occurred PRE/POST a standardized forty-minute high intensity bodyweight exercise bout. Participants then completed two seven day identical blinded supplement protocols in randomized fashion separated by a seven day washout period: 1^st^ double-blind (1DB); washout; second double-blind (2DB). The double-blind (DB) supplement protocol included prepackaged servings of placebo (PLA) or creatine monohydrate (CRE) ingesting 4g in the morning and at night for seven days. After each DB, participants repeated the exercise and ST protocol separated by a seven day washout. Two ANOVA tests analyzed cognition before and after three separate conditions: CONT, PLA, and CRE before exercise (PRE), post-exercise (POST), and PRE/POST exercise for each condition. LSD post hoc tests evaluated pairwise comparisons when significance was observed (*p* < 0.05).

**Results**

Statistical analysis revealed that ST were affected in the PRE (F_1,12_ = 16.0, *p* < 0.002, η^2^ = 0.58) and POST (F_1,12_ =17.0, *p* = 0.001, η^2^= 0.59) timepoints. PRE/POST comparisons revealed that both PLA and CRE had an effect (PLA: F_2,12_ =100.6, *p* < 0.001; η^2^ = 0.89) (CRE: F_2,12_ =112.4, *p* < 0.001; η^2^= 0.90). Pairwise comparisons revealed no differences in CONT PRE/POST exercise (*p* > 0.05), however PLA and CRE both significantly increased ST scores PRE/POST exercise (PLA: PRE 24.2±13.2, POST: 29.3±9.4; *p* = 0.004) (CRE: 29.4±10.4, POST 34.2±11.6; *p* = 0.002) respectively. PRE and POST timepoints revealed PLA and CRE were significantly different from baseline (PRE: PLA *p* = 0.005, CRE *p* = 0.002) (POST: PLA *p* = 0.002, CRE *p* = 0.001), but PLA and CRE were not different in either time point (*p* > 0.05).

**Conclusions**

Significant increases by condition PRE/POST exercise suggest that exercise, PLA and CRE increase cognition. Lack of relevant differences within timepoints (PRE and POST) despite cognition being 16.0% and 12.6% higher with CRE compared to PLA is likely due to high within group variation.

**Acknowledgments**

A special thank you to the Longwood University and Hampden Sydney College ROTC cadets for their participation in this study.

## A67 Resting metabolic rate and heart rate are not effected by music or smart phone use in college aged women

### Edward H. Robinson IV ehrobinson@meredith.edu

#### Exercise and Sports Science, Department of Nutrition, Health, and Human Performance, Meredith College, 3800 Hillsborough St, Raleigh, NC, 27607, USA

**Background**

Determining energy need is a common and often important component of nutritional assessment. In the field of clinical dietetics and nutritional research, it is often necessary to measure and insure a proper balance between energy intake and expenditure. The use of indirect calorimetry to measure resting metabolic rate (RMR) is a standard test. The testing procedures are usually carried out with the participant sitting or lying supine in an isolated and quiet environment to insure that a true RMR is achieved. However, there has been little to no research conducted to determine if this is a necessary component of the testing procedure [1]. To date, no study has examine the effects of self-selected music or light smart phone usage effect on RMR.

**Methods**

Twenty-one healthy women (Age: 20±0.74 yrs; Weight; 65.14±9.76 Kg) volunteered to participate in this study. All participants were requested to refrain from caffeine intake for 12 hours and eating 4 hours prior to testing. RMR was measured in one 45 minute, crossover design session with three randomly assigned 15 minute measurement periods where individuals either relaxed with no external stimulation, were allowed to play a self-selected playlist of music, or were allowed to use technology for light functions—reading social media or testing, but no applications involving sound. VO_2_ and heart rate were measured for 15 minutes with the first 5 minutes of each treatment segment discarded and the remaining 10 minutes analyzed. Outcomes were measured in SPSS utilizing a repeated measures ANOVA.

**Results**

The repeated measures ANOVA determined there were no significant differences observed for any of the treatment conditions for RMR (*F*(1.489,0.179)=0.948, *p*=0.395). No significant differences were seen for heart rate (*F*(1.787, 28.291)=2.588, *p*=0.098) between the three testing conditions either.

**Conclusions**

These findings suggest that self-selected music or light smart phone usage do not alter RMR in college aged women. The common practice of isolating or restricting individuals from external stimuli during testing may not be necessary to obtain a true RMR.

**References**

1. Compher C, Frankenfield D, Keim N, Roth-Yousey L. Best practice methods to apply to measurement of resting metabolic rate in adults: a systematic review. Journal of the American Dietetic Association. 2006 Jun 1;106(6):881-903.

## A68 Similar results for heart rate variability threshold during arm ergometry exercise in normobaric hypoxia and normoxia

### Nicolas W. Clark^1^, David H. Fukuda^1^, Michael B. La Monica^1^, Tristan M. Starling-Smith^1^, Valéria L. G. Panissa^2^, Jeffrey R. Stout^1^

#### ^1^University of Central Florida, Orlando, FL, 32816, USA; ^2^University of São Paulo, São Paulo, SP, 05508-010, Brazil

##### **Correspondence:** Nicolas W. Clark (nicolas.clark@knights.ucf.edu)

**Background**

Heart rate variability (HRV) has gained popularity due to its feasibility in monitoring autonomic nervous system activity. During exercise, HRV threshold (HRVT) may signify vagal withdrawal while coinciding with the first ventilatory threshold. Among time domain methods to analyze HRVT, the standard deviation of normal R-R intervals (SDNN) and the root mean square of successive differences of R-R intervals (RMSSD) are the most common in the literature, while non-linear analysis of Poincaré plots and standard deviation 1 (SD1) has also been employed. Normobaric hypoxia has been successfully utilized as a method to validate the sensitivity of HRVT and other fatigue thresholds during lower-body aerobic exercise. However, HRVT response during upper-body exercise has yet to be examined. The purpose of this study was to evaluate the sensitivity of HRVT during a graded exercise test (GXT) utilizing arm ergometry under hypoxic and normoxic conditions.

**Material and methods**

Fifteen recreationally-active men (20.3±5.7 y; 176.5±0.05 cm; 85.5±11.9 kg) volunteered for this study. Participants performed the GXT under normobaric hypoxia (FiO2 = 14.0±0.1%) and normoxia (FiO2 = 20.1±0.2%). Prior to testing, each participant was fitted with a heart rate monitor to record R-R intervals. Data were recorded and later analyzed via Kubios HRV software. Heart rate variability assessed using SDNN, RMSSD, and SD1 values was quantified every 5 seconds using a 30-second rolling average. Two separate evaluators determined the HRVT by visual inspection. In the case of disagreement, a third evaluator made the final determination. A two-way (condition×threshold) repeated measures ANOVA was used to compare absolute and relative power output values at HRVT under hypoxic and normoxic conditions.

**Results**

No significant main effects or condition×threshold interactions (p>0.05) were noted for absolute (59-65 watts) or relative (44-47% of peak) power outputs at HRVT. The third evaluator was required for 17 out of the 90 tests analyzed, 13 being from hypoxia and 4 from normoxia.

**Conclusions**

The evaluation of HRVT using SDNN, RMSSD, and SD1 did not differ during arm ergometry exercise under hypoxic and normoxic conditions. Previous studies have established that the increased proportion of type II muscle fibers and reduced absolute muscle mass in the upper-body compared to lower body results in decreased aerobic capacity and a greater reliance on the anaerobic system during exercise. Therefore, unique morphological and physiological characteristics of the upper body musculature may have limited the influence of altered environmental conditions on HRVT.

**Acknowledgements**

This research did not receive any specific grant from funding agencies in the public, commercial, or not-for-profit sectors.

## A69 Evaluation of caloric expenditure predictors and RMR estimation equations throughout the annual training cycle in division I female soccer athletes

### Kyle S. Levers, Troy M. Purdom, Jacob Giles, Lindsey Brown, Chase McPherson, Patrick Martin, Natalie Fry

#### Department of Health, Athletic Training, Recreation and Kinesiology, Longwood University, Farmville, VA 23901, USA

##### **Correspondence:** Kyle S. Levers (leverskyle@gmail.com)

**Background**

Estimating resting metabolic rate (RMR) is common for nutrition professionals, particularly those working with large groups of athletes. RMR estimation equations (RMREEs) utilize various factors to predict caloric expenditure, including: height, body weight (BW), age, gender, and fat free mass (FFM). Currently, the impact of collegiate annual periodized training on anthropometrics utilized to estimate caloric expenditure (RMREEs) is unknown. Therefore, the purpose of this study was to evaluate how RMR predictors fluctuated across the annual training cycle and their effect on multiple RMREEs.

**Materials and Methods**

Caloric expenditure was estimated using four equations: Harris-Benedict (HB), Cunningham (CH), Owen, and Livingston & Kohlstadf (LK) in 17 Division I female collegiate soccer athletes (Mean ± SD: 19.24 ± 1.03yrs, 165.68 ± 6.95cm, 61.77 ± 6.06kg, 49.17 ± 4.17kg FFM, 20.24 ±3.29% BF). Demographic and anthropometric data was collected across six blocks: post-2016 competitive season (B1), detraining (B2), spring season (B3), and pre- (B4), mid- (B5), and post- 2017 competitive season (B6). Trained researchers utilized the Jackson and Pollock female- specific 3-site skinfold to measure body density and the Brozek formula to estimate body composition. Two repeated measure ANOVAs evaluated changes in demographics, anthropometrics, and RMREEs across all blocks. Separate one-way ANOVAs compared RMREEs within each block.

**Results**

FFM increased from B2-B3 (Δ1.79kg, *p*=0.032) and B2-B6 (Δ1.79kg, *p*=0.011) with no change in BW or height across all blocks (*p*>0.05). CH RMR estimates paralleled FFM increases from B2-B3 (Δ 39.46kcal, *p*=0.032) and B2-B6 (Δ 28.10kcal*, p*=0.011). Compared to CH, estimated RMRs from HB (avg -122.69kcal, *p*<0.009), Owen (avg -219.89kcal, *p*<0.001), and LK (avg -189.17kcal, *p*<0.001) were significantly lower at every time point. HB-derived RMRs were significantly higher than Owen in B1 (108.31kcal, *p*=0.008), B2 (102.71kcal, *p*=0.010), and B5 (88.77kcal *p*=0.039). Participant age increased in B4 (Δ0.59yrs, *p*=0.003), B5 (Δ0.94yrs, *p*<0.001), and B6 (Δ0.76yrs, *p*<0.001) from B1.

**Conclusions**

Results indicate that CH RMR estimations follow FFM fluctuations across the annual training calendar and are considered more accurate for collegiate athlete populations [1]. HB, Owen, and LK RMR estimations did not change across the year, paralleling the lack of change in their RMR predictors: BW and/or height. Participants age increased throughout the year, but age did not impact HB and LK caloric estimations. HB/Owen RMR prediction differences are likely attributed to HB height inclusion. In this population, BW and height did not change, therefore the RMREEs that utilize these metrics are less sensitive. CH utilizes FFM-based RMR estimation and therefore is more sensitive to annual training load variation in athletic populations. Further research is necessary to validate CH RMR sensitivity with training load periodization across the annual calendar.

**References**

1. Burke, L. and Deakin, V. **Energy requirements of the athlete: assessment and evidence of energy efficiency.** In *Clinical Sports Nutrition*. 5^th^ Ed. 2015. McGraw-Hill Education. 120-127.

## A70 The effect of a 6-week NFL draft preparation training program on standing broad jump performance

### Matthew A Lee^1^, Trisha A VanDusseldorp^1^, Joe Boone^1^, Gannon Hampton^1^, Jacob McNabb^1^, Matthew T Stratton^1^, Megan Barie^1^, Andrew Modjeski^1^, Yuri Feito^1^, Robert Wildman^2^, Gerald T Mangine^1^

#### ^1^Department of Exercise Science, Kennesaw State University, Kennesaw, GA, 30144, USA; ^2^Department of Nutrition & Food Sciences, Texas Women University, Denton, TX, 76204, USA

##### **Correspondence:** Trisha A VanDusseldorp (tvanduss@kennesaw.edu)

**Background**

The broad jump drill can be used as a measurement for numerous athletic qualities, such as leg power and strength, as well as balance. These certain variables have been demonstrated to improve with training that consists of speed, agility, plyometric, and resistive type work. This study’s purpose was to determine the effect of a 6-week NFL combine-preparation training program on broad jump performance, specifically changes in peak power, peak force, peak rate of force development, peak velocity, and acceleration.

**Methods**

Seventeen collegiate football players (21.4 ± 1.1 yrs, 185.7 ± 5.6 cm, 97.6 ± 13.8 kg) participated in a 6-week NFL Combine/Pro Day training program. The training program consisted of a traditional and assisted/resisted speed training regimen, agility and reaction drills, as well as resistance and plyometric training four days per week; split by one aquatic recovery day weekly. Baseline (PRE) and after 6-weeks of training (POST) values of peak power, peak force, peak rate of force development [peak RFD], peak velocity, and acceleration were obtained with a 1080 sprint kinetics device. The statistical analysis consisted of paired-samples T-tests of these PRE and POST values to determine if changes were significant (p ≤ 0.05).

**Results**

No significant changes (p > 0.05) were seen concerning the following variables, PRE – POST respectively: peak power (219.5 ± 54.4 W – 241.7 ± 29.8 W), peak force (51.6 ± 6.0 N – 53.9 ± 3.0 N), peak RFD (2872.8 ± 729.6 – 2925.3 ± 650.9 N · s^-1^), peak velocity (4.8 ± 0.9 – 5.1 ± 0.4 m/s), and acceleration (25.0 ± 6.4 – 27.4 ± 6.0 m/s^2^).

**Conclusion**

This 6-week NFL combine-preparation training program had no effect on broad jump performance, as there were no significant changes in peak power, peak force, peak RFD, peak velocity, or acceleration.

## A71 The effect of a 6-week NFL draft preparation training program on bilateral differences in the 40yd sprint

### Paul Serafini^1^, Trisha A VanDusseldorp^1^, Joe Boone^1^, Gannon Hampton^1^, Jacob McNabb^1^, Matthew T Stratton^1^, Megan Barie^1^, Andrew Modjeski^1^, Yuri Feito^1^, Robert Wildman^2^, Gerald T Mangine^1^

#### ^1^Department of Exercise Science, Kennesaw State University, Kennesaw, GA, 30144, USA; ^2^Department of Nutrition & Food Sciences, Texas Women University, Denton, TX, 76204, USA

##### **Correspondence:** Trisha A VanDusseldorp (tvanduss@kennesaw.edu)

**Background**

The 40yd sprint for time is a test commonly included in the NFL Combine and Pro Days, used to evaluate the potential of NFL draft candidates. Examining and improving bilateral performance differences at various stages of the sprint (i.e., first five strides, start to peak velocity, and total sprint) may improve acceleration to peak velocity, maximal sprint velocity, total sprint time, and reduce the occurrence of injuries. The aim of this study was to determine the effect of a 6-week NFL draft preparation-training program on bilateral performance and sprint kinetics for the 40yd sprint.

**Methods**

Fifteen collegiate football players (physical characteristics: 21.5 ± 1.1 yrs; 186.1 ± 5.8 cm; 97.2 ± 14.7 kg) were included in this study. All participants reported to a sports performance facility to engage in a comprehensive, 6-week training program (TP) intended to improve a broad range of performance and skill-related tasks specific to NFL Draft testing. The TP consisted of four weekly sessions of resistance training (RT) and plyometric training, traditional and resisted/assisted speed, agility, and reaction training. Participants attended a pool recovery session once per week. RT sessions consisted of loads corresponding to a 5-12 repetition maximum. Values for sprint kinetics (peak velocity, peak power, distance, time, average force, average power) were collected from the first five strides (5S), start to peak velocity (SPV), and total sprint (TS) on a 1080 sprint device. All testing of bilateral 40yd sprint performance occurred at baseline and 6-weeks post.

**Results**

Paired samples T-tests revealed significant improvement (p<0.05) in bilateral percent differences from pre to post for peak velocity (3.2 ± 2.3%; m/s) and peak power (9.1 ± 7.9%; W) during SPV. TS significant improvements (p<0.05) were observed in bilateral percent differences from pre to post for distance (7.1 ± 7.6%; m), time (6.4 ± 6%; s), peak velocity (1.9 ± 1.4%; m/s), average force (2.7 ± 5.1%; N), peak power (7.4 ± 7.4%; W), and average power (4.6 ± 7.4%; W). No differences were found among other time points for sprint time.

**Conclusion**

These data indicate that following a 6-week NFL Draft training program, participants demonstrated an improved equalization of leg contribution across a 40yd sprint.

## A72 Effect of Dynamine™ with and without Teacrine^®^ over four weeks of continuous use on cardiovascular function and psychometric parameters: a pilot study

### Matthew T. Stratton^1^, Alyssa J. Holmes^1^, Alyssa R. Bailly^1^, Andrew Modjeski^1^, Megan Barie^1^, Paul Serafini^1^, Yuri Feito^1^, Gerald T. Mangine^1^, Karleena R. Tuggle^2^, Tiffany A. Esmat^1^, Garrett M. Hester^1^, Trisha A. VanDusseldorp^1^

#### ^1^Department of Exercise Science and Sport Management, Kennesaw State University, Kennesaw, GA 30144, USA; ^2^Atlanta Medical Center, Atlanta, GA, 30312, USA

##### **Correspondence:** Trisha A. VanDusseldorp (tvanduss@kenensaw.edu)

**Background**

Methylliberine (1,7,9-tetramethyluric acid; Dynamine™) is a derivative of caffeine that naturally occurs in kucha tea, many species of *Coffae*, and the cupuacu fruit. Theacrine (TeaCrine^®^) and Dynamine™ are isomers commonly found in energy supplements. TeaCrine has previously been shown to enhance feelings of energy, cognition, and exercise performance. However, to date, there are no published human safety data available on Dynamine. The purpose of this study was to examine the effects of four weeks of continuous use of Dynamine with and without TeaCrine on changes in heart rhythm (electrocardiogram; ECG), resting heart rate (RHR), blood pressure (BP), and psychometric parameters (PP).

**Materials and methods**

Twenty-four college aged men (n=13) and women (n=11) were randomly assigned to one of five groups: low dose Dynamine (100mg), high dose Dynamine (150mg), low dose Dynamine with TeaCrine (100mg Dynamine + 50mg TeaCrine), high dose Dynamine with TeaCrine (150mg Dynamine + 25mg TeaCrine), and placebo (125mg Maltodextrin). Participants were then assessed for baseline ECG, RHR, BP and PP (energy, feeling of productivity, alertness, desensitization, motivation to do physical tasks, motivation to do mental tasks, and perceived level of focus) using visual analogue scales (VAS; 1-10 scale) every 30 minutes until 120 after the first dosage. Following the initial assessment, participants were instructed to take their supplement upon waking with approximately 12oz of water for four consecutive weeks. VAS were assessed one and two weeks post initial measures. After the four weeks of supplementation participants returned to repeat the initial measures.

**Results**

No group × time interactions were noted for RHR, BP, and PP. Main effects for time were noted for corrected QT interval (p = 0.016) pre to post four week supplementation, as well as R to R and P to P intervals pre to 60 min post at both visits (p = 0.001). Main effects for time were noted for increases in energy (p < 0.001), alertness (p ≤ 0.013), productivity (p ≤ 0.03), and motivation to perform mental tasks (p ≤ 0.028) for all time points assessed compared to pre-supplementation. No adverse events were reported in participants that completed the investigation.

**Conclusion**

These preliminary data suggest that Dynamine alone or in combination with TeaCrine does not significantly affect heart rhythm, RHR, BP, or PP following acute or chronic supplementation at the dosages used in this study. We will expand this investigation to an additional 100 participants.

**Acknowledgements**

Compound Solutions, Inc. grant

## A73 Short-term effects of ingesting a food bar containing whey protein and isomalto-oligosaccharides on glycemic and insulinemic responses to an acute resistance-exercise bout and sprint conditioning

### Tyler J. Grubic, Ryan Sowinski, Ben E. Nevares, Susannah L. Williamson, Victoria M. Pizzitola, Aimee G. Reyes, Chris Rasmussen, Peter S. Murano, Mike Greenwood, Conrad P. Earnest, Richard B. Kreider

#### ^1^Exercise & Sport Nutrition Lab, Human Clinical Research Facility, Texas A&M University, College Station, TX, 77446, USA

##### **Correspondence:** Richard B. Kreider (rbkreider@tamu.edu)

**Background**

Prior research in our lab demonstrated that ingesting a food bar (FB) containing a whey protein blend and the plant fiber isomalto-oligosaccharides elicited a lower glycemic but similar insulin response in comparison to a reference carbohydrate in healthy adults. (*Austin J Nutri Food Sci 6(1):1099, 2018*). This study examined whether ingesting this FB would serve as a low glycemic and insulinogenic food option surrounding intense exercise.

**Methods**

Twelve resistance-trained males (82.8±10 kg, 14.2±3.7% fat; BMI 26.3±3.7 kg/m^2^) participated in an un-blinded, randomized, counterbalanced, cross-over trial. Participants donated fasting venous blood samples and completed a Readiness to Perform (RTP) and Eating Satisfaction (ES) surveys prior to ingesting a FB (*Fitjoy™*) containing 20 g of a whey protein blend and 25 g of isomalto-oligosaccharides plant fiber (*VitaFiber™*, 13 g fiber, 4 g sugar) and 7g fat (1.5 g saturated) or 25 g of dextrose gel placebo (PLA). Thirty minutes after ingesting the FB or PLA, participants performed a resistance training workout (3 sets of 10 repetitions at 70% 1RM on 11 exercises) followed by sprint condition drills (3 x 40 yd and 3 x Nebraska drills). Midway and following exercise, participants again ingested the FB or PLA. Glucose was determined via finger sticks pre-ingestion, pre-exercise, midway-exercise, post-exercise, post-sprint, and post-isokinetic testing. Venous blood samples and RTP and ES surveys were obtained midway and post-exercise. Data were analyzed by general linear model (GLM) repeated measures multivariate and univariate statistics and are presented as mean [95% CI] changes from baseline and effect sizes as partial eta-squared (n^2^, 0.01 = small, 0.06 = medium, 0.13 = large).

**Results**

Glucose was significantly greater (+25%) following 30-min post ingestion in the PLA compared to FB trial (151.5 [137.2, 165.8]; 111.2 [96.9, 125.5] mg/dL, p<0.001). Glucose values with FB remained within normal values (94.6±11 to 111.2±18.6 mg/dL) while greater variability was seen with PLA (95.8±19.6 to 151.5±28.7 mg/dL). No differences were seen between groups in glucose AUC. Both groups demonstrated similar peak insulin responses immediately post exercise with no differences between treatments although the FB displayed a 28% higher insulin peak post training (PLA 11.2 [5.6, 16.8]; FB 15.5 [9.9, 21.1] uIU/mL, p=0.27, n^2^=0.149). Venous blood glucose taken 48-h recovery was lower in FB group, although no significantly different (FB -0.05 [-0.28, 0.18]; PLA 0.23 [-0.002, 0.46], p=0.09, n^2^=0.13). Participants also reported significantly greater satisfaction from food, feeling of fullness, and amount of energy with less feelings of hunger with FB. No significant differences over time or between treatments were observed in ratings of symptoms of hypoglycemia, or perceptions to RTP questionnaires.

**Conclusions**

The FB examined in this study better maintained glucose responses during an intense bout of resistance exercise and sprint conditioning with a similar insulin response suggesting that ingestion of this FB around exercise can serve as a good food choice.

**Acknowledgements**

This study was supported by the Exercise & Sport Nutrition Lab at Texas A&M University. CPE served as a Director of Clinical Sciences for Nutrabolt. RBK served as a university approved scientific advisor for Nutrabolt. PSM served as quality assurance supervisor.

## A74 Short-term effects of a ingesting a food bar containing whey protein and isomalto-oligosaccharides on performance outcomes and recovery from an acute resistance-exercise bout and sprint conditioning

### Tyler J .Grubic, Ryan Sowinski, Ben E. Nevares, Susannah L. Williamson, Victoria M. Pizzitola, Aimee G. Reyes, Chris Rasmussen, Peter S. Murano, Mike Greenwood, Conrad P. Earnest, Richard B. Kreider

#### Exercise & Sport Nutrition Lab, Human Clinical Research Facility, Texas A&M University, College Station, TX, 77446, USA

##### **Correspondence:** Richard B. Kreider (rbkreider@tamu.edu)

**Background**

Prior research in our lab demonstrated that ingesting a food bar (FB) containing a whey protein blend and the plant fiber isomalto-oligosaccharides elicited a lower glycemic but similar insulin response in comparison to a reference carbohydrate in healthy adults. (*Austin J Nutri Food Sci 6(1):1099, 2018*). This study examined whether ingesting this FB would affect performance and/or recovery during intense resistance and sprint conditioning training.

**Methods**

Twelve resistance-trained males (82.8±10 kg, 14.2±3.7% fat; BMI 26.3±3.7 kg/m2) participated in an un-blinded, randomized, counterbalanced, cross-over trial. Participants donated fasting venous blood samples, graded visual rating scale (GRPS) measurements at 3 sites (distal vastus medials [VM], distal vastus lateralis [DVL] and mid-lateral vastus lateralis [MLVL]), and isokinetic leg extension/flexion maximal voluntary contractions (MVCs) prior to ingesting a FB (*Fitjoy™*) containing 20 g of a whey protein blend and 25 g of isomalto-oligosaccharides plant fiber (*VitaFiber™*, 13 g fiber, 4 g sugar) and 7g fat (1.5 g saturated) or 25 g of dextrose gel placebo (PLA). Thirty minutes after ingesting the FB or PLA, participants performed a resistance training workout (3 sets of 10 repetitions at 70% 1RM on 11 exercises with 2-min recovery between sets and exercises) followed by sprint conditioning drills (3 x 40 yd sprints [FYD] and 3 x Nebraska agility drills [NAD] with 1:4 work:rest ratio). Venous blood samples, GRPS, and MVC assessments were also assessed post-exercise and at 48-h recovery. Participants repeated the experiment while ingesting the alternate supplement 7-d later. Data were analyzed by general linear model (GLM) repeated measures multivariate and univariate statistics and are presented as mean [95% CI] changes from baseline and effect sizes as partial eta-squared (n^2^, 0.01 = small, 0.06 = medium, 0.13 = large).

**Results**

Assessment of mean 95% CI indicated that participants experienced significantly less pain post workout compared to pre-exercise with FB ingestion as indicated at GPRS VM (FB 0.29 [-0.99, 1.57]; PLA 1.88 [0.60, 3.17] cm, p=0.08, n^2^=0.130). GPRS DVL (FB 1.45 [-0.02, 3.12]; PLA 2.13 [0.45, 3.80] cm, p=0.56, n^2^=0.016) and GPRS MLVL (FB 1.53 [-0.28, 3.33]; PLA 2.32 [0.51, 4.12] cm, p=0.53, n^2^=0.018) were significantly lower at 48-h recovery compared to pre-exercise for FB. MVCs were not significantly different over time or between treatments. NAD sprint-2 was significantly faster for FB group (FB -0.21 [-0.36, -0.60]; PLA 0.13 [-0.28, 0.02] sec, p=0.42) compared to baseline. Both groups were significantly faster in NAD sprint-3. No significant differences in time or between group differences were observed for FYD. Leg-press volume was significantly lower in PLA during set 2 (FB 0.00 [-34.06, 34.06]; PLA -42.71 [-76.77, -8.65] reps*kg, p=0.08, n^2^=0.133) and set 3 (FB -7.94 [-112.17, 96.30]; PLA -130.79 [-235.02, -26.55] reps*kg, p=0.09, n^2^=0.120) when compared to baseline. Blood urea nitrogen to creatinine ratio (BUN:Cre) were maintained in FB at 48-h recovery where PLA decreased compared to baseline (FB –1.16 [-2.92, 0.61]; PLA -3.09 [-4.85, -1.34] (mmol/L)/(umol/L), p=0.121, n^2^=.106).

**Conclusions**

Participants ingesting the FB exhibited lowered muscle soreness compared to a PLA, better maintained NAD repeated sprint ability, and better leg press performance. It appears this whey protein bar could serve as an advantageous pre-exercise food choice compared to a matched carbohydrate alone.

**Acknowledgements**

This study was supported by the Exercise & Sport Nutrition Lab at Texas A&M University. CPE served as a Director of Clinical Sciences for Nutrabolt. RBK served as a university approved scientific advisor for Nutrabolt. PSM served as quality assurance supervisor.

## A75 A comparison of actual dietary intake versus recommended intake of female Lacrosse layers

### Andrew R. Jagim^1,2^, Hannah Zabriske^1^, Patrick S. Harty^1^, Richard Stecker^1^, Chad Kerksick^1^

#### ^1^Exercise & Performance Nutrition Laboratory, Lindenwood University, St. Charles, MO 63301, USA; ^2^Research Collaborator, Mayo Clinic Health Systems, La Crosse, WI 54601, USA

##### **Correspondence:** Andrew R. Jagim (ajagim@lindenwood.edu)

**Background**

The purpose of this study was to compare calculated ISSN recommendations for daily energy and macronutrient intake to the actual in-season dietary intake of female Lacrosse players.

**Materials and methods**

Twenty-two female NCAA Division II Lacrosse players (169.9 ± 6.2 cm; 69.7 ± 10.2 kg; 27.3 ± 3.3 % body fat) completed a 4-day monitoring period during in-season competition. Athletes were outfitted with a combined heart rate and activity monitor over four consecutive days and completed four-day food and fluid records to assess total daily energy expenditure (TDEE) and dietary intake. Participants were also assessed for body composition which was used to calculate recommended intake values and total daily energy expenditure. Dietary intake was self-reported using a commercially available food tracking program (*MyFitnessPal©, USA*). Daily average values were calculated for total and relative energy, protein, carbohydrate, and fat intake. These values were then compared to nutritional recommendations put forth by the ISSN for team sport athletes undergoing a similar level of training, which equated to recommended values of 65 kilocalories per kilogram of body weight per day (kg/d), 6.5 g/kg/d, and 1.8 g/kg/d for total energy, carbohydrates and protein, respectively. Recommendations for energy intake were also compared to measured TDEE from activity monitors. A recommended fat intake equating to 30% of the recommended energy intake was used. Paired sample t-tests were used to compare differences between calculated recommended values and actual intake.

**Results**

Significant differences for all energy and macronutrient recommendations were observed (p<0.001) when compared to actual intakes. These differences were present for both total and relative daily values. For energy and all macronutrient recommendations, athletes consumed well below the recommendations as outlined in Table 1.

**Conclusions**

Athletes tended to under consume for both energy and macronutrient content when compared to the ISSN Recommendations, based on their body size and level of training. However, when daily energy intake was compared the measured TDEE, the measured TDEE was far below the ISSN recommendation and subsequently the magnitude of daily energy deficiency was reduced. Therefore, it is possible the energy recommendation of 65 kcal/kg/day is too high for Division II female Lacrosse players in-season. Smaller institutions frequently do not have the resources to hire full-time nutritional staff; therefore, it may be beneficial for coaches to offer a nutrition-education program to ensure their players are meeting the energy requirements for their body size and level of training.

**Table 1 (abstract A75).** Comparison of recommended dietary intake versus actual intake

n=22	ISSN Recommendations	Actual Intake	Delta (Actual – Recommended)	p value
Total Energy Intake (kcal/d)	4,532 ± 664	2,140 ± 412	-2,391 ± 812	p<0.001
Relative Energy Intake (kcal/kg/d)	65.0 ± 0.0	31.2 ± 7.8	-33.8 ± 7.8	p<0.001
Total Energy (kcal/d)*	3,694 ± 838	2,140 ± 412	-1,553 ± 967	p<0.001
Relative Energy (kcal/kg/d)*	53.5 ± 12.4	31.2 ± 7.8	-22.4 ± 14.1	p<0.001
Total CHO (g/d)	453.2 ± 66.4	235.2 ± 74.1	-218.0 ± 94.8	p<0.001
Relative CHO (g/kg/d)	6.5 ± 0.0	3.4 ± 1.2	-3.1 ± 1.2	p<0.001
Total PRO (g/d)	125.5 ± 18.4	77.3 ± 19.4	-48.3 ± 31.5	p<0.001
Relative PRO (g/kg/d)	1.8 ± 0.0	1.1 ± 0.4	-0.6 ± 0.4	p<0.001
Total FAT (g/d)	151.1 ± 22.9	85.8 ± 22.9	-65.3 ± 35.1	p<0.001
Relative FAT (g/kg/d)	2.2 ± 0.0	1.3 ± 0.4	-0.9 ± 0.4	p<0.001

Data are mean±SD. n=26

## A76 Glycemic and insulinemic response to a commercial food bar containing a whey protein blend with isomalto-oligosaccharides plant fiber as the carbohydrate source

### Tyler J. Grubic^1^, Ryan L. Sowinski^1^, Ryan L. Dalton^1^, Christopher J. Favot^1^, Brittany Sanchez^1^, Patrick B. Collins^1^, Aimee G. Reyes^1^, Christopher Rasmussen^1^, Mike Greenwood^1^, Conrad P. Earnest^1,2^, Richard B. Kreider^1^

#### ^1^Exercise & Sport Nutrition Lab, Human Clinical Research Facility, Texas A&M University, College Station, TX, 77446, USA; ^2^Nutrabolt, Bryan, TX, 77801, USA

**Background**

This study examined the pharmacokinetic glucose and insulin response of a commercial Food Bar (Bar, Fitjoy™) containing a whey protein blend (20g), carbohydrate (25g) as isomalto-oligosaccharides plant fiber (VitaFiber™; 13 g fiber, 4 g sugar) fat (7g total, 1.5g saturated) (220 kcals) vs. 25 g of dextrose (96 kcals) placebo (PLA).

**Methods**

Twenty apparently healthy participants (50% female; BMI <24.9 kg/m2) participated in an un-blinded, randomized, cross-over trial. Participants donated a 10-h fasted blood sample prior to ingestion of their respective treatments. Additional blood samples were taken at 10, 20, 30, 60, 90, 120 and 240-min post-ingestion. Glucose and Insulin AUC were analyzed via a univariate general linear model (GLM) for treatment, treatment order, adjusted for sex and respective 0-min measures of glucose and insulin, as no sex- by-treatment effects were observed. A repeated measure GLM with the same adjustments was used to assess individual time point blood values. Data are mean ± SD, mean change (95% CI) and effect sizes as partial eta-squared (n^2^, 0.01 = small, 0.06 = medium, 0.13 = large).

**Results**

Glucose AUC was significantly greater following PLA (688±78 mmol-h/L) compared to Bar ingestion (599±50 mmol-h/L, n^2^, 0.08, P=0.001). No significant differences were observed between treatments for insulin AUC (PLA 1848±971; Bar 2136±1073 pmol-h/L, n^2^ = 0.14, P=0.38). Both groups demonstrated peak glucose concentrations at 30-min with significantly higher peak glucose values observed with PLA (PLA 7.51±1.24; Bar 5.61±0.62 mmol/L, P=0.001). At glucose peak, the Bar (5.61 mmol-h/L) was 25.3% lower than the PLA (7.51 mmol-h/L). For insulin, both groups demonstrated similar peak insulin concentrations at 30-min (PLA 27.83±15.17; Bar 30.87±20.68 pmol/L) with no differences between treatments. The insulin:glucose ratio was significantly lower at 30-min [PLA (3.65, 95% CI, 2.47, 4.82) vs. Bar (5.33, 95% CI, 4.15, 6.50)] and 60-min [PLA (2.69, 95% CI, 1.74, 3.65) vs. Bar (4.11, 95% CI, 3.16, 5.07)].

**Conclusions**

The estimated glycemic load of the Bar examined in this study ranges from 3.84-8.39 and exhibits a 25.3% lower glycemic response, but similar insulin response, compared to a dextrose PLA.

**Acknowledgments**

This study was supported by Nutrabolt (Bryan, TX) through an unrestricted research grant provided to Texas A&M University. CP Earnest serves as a Director of Clinical Sciences for Nutrabolt. RB Kreider serves as a university approved scientific advisor for Nutrabolt . PS Murano serves as quality assurance supervisor.

## A77 Short-term effects of a ready-to-drink pre-workout beverage on blood chemistry and self-reported side effects

### Patrick B. Collins^1^, Christopher J Favot^1^, Conrad P. Earnest^1^, Ryan L. Dalton^1^, Ryan J Sowinski^1^, Tyler J. Grubic^1^, BK Sanchez^1^, Aimee G Reyes^1^, Adriana M. Coletta^1^, Christopher Rasmussen^1^, Mike Greenwood^1^, Peter S. Murano^2^, Richard B. Kreider^1^

#### ^1^Exercise and Sport Nutrition Lab, Human Clinical Research Facility, Texas A&M University, College Station, TX, 77446, USA; ^2^Institute for Obesity and Program Evaluation, Texas A&M University, College Station, TX, USA; ^3^Nutrabolt, Bryan, TX, 77446, USA

##### **Correspondence:** Richard B. Kreider (rbkreider@tamu.edu)

**Background**

This study examined the effects of short-term ingestion of a ready-to-drink pre-workout supplement (RTD) on blood chemistry responses and self-reported side effects.

**Methods**

Resistance-trained participants (n=25) ingested in a randomized, double-blind, crossover manner a: (1) Dextrose placebo (PLA, 12g) and, (2) RTD containing caffeine (200mg), β-alanine (2.1g), niacin (65mg), folic acid (325mcg), Vitamin B12 (45mcg), and arginine nitrate (1.3g) in a randomized, crossover manner for 7-d, interspersed by 7d washout. Fasting 8h blood samples were assessed for plasma nitrate, whole blood counts, liver, kidney, and muscle function, and a metabolic panel. A side effects questionnaire was obtained twice on each testing day (Days 1, 2, 6, and 7). Data were analyzed by MANOVA adjusted for sex and relative caffeine dose and presented as mean change from baseline (95% CI). The frequency of side effects and blood chemistry changes from baseline were analyzed using Pearson’s Chi Square analysis.

**Results**

Plasma nitrate concentration increased from Day 1 to Day 7 in the RTD treatment (0.06, 95% CI, 0.003, 0.124 μM) with no change in PLA (0.02, 95% CI, -0.05, 0.08 μM). No significant overall statistical effects were observed for remaining blood chemistry markers. Chi Square analysis of blood chemistry changes from Day 1 to Day 7 did not reveal any significant change from baseline with the exception of nitrates. No overall effects were observed in severity of side effect (p=0.42). As expected with β-alanine ingestion, participants consuming the RTD tended to report skin tingling or paresthesia (p=0.07).

**Conclusions**

Results indicate that use of this RTD prior to exercise for up to seven days does not have a negative impact on liver, kidney, or muscle blood chemistries or cause any unexpected side effects in college-aged, resistance-trained males and females.

**Acknowledgements**

This study was supported by Nutrabolt (*Bryan, TX*) through an unrestricted research grant provided to Texas A&M University. CP Earnest served as a Director of Clinical Sciences for Nutrabolt. RB Kreider served as a university approved scientific advisor for Nutrabolt. PS Murano served as quality assurance supervisor.

## A78 Short-term effects of a ready-to-drink pre-workout beverage on skeletal muscle strength and endurance

### Patrick B. Collins^1^, Conrad P. Earnest^1^, Ryan L. Dalton^1^, Ryan J. Sowinski^1^, Tyler J. Grubic^1^, Brittany K Sanchez^1^, Christopher J. Favot^1^, Aimee G. Reyes^1^, Adriana M. Coletta^1^, Christopher Rasmussen^1^, Mike Greenwood^1^, Peter S Murano^2^, Richard B Kreider^1^

#### ^1^Exercise and Sport Nutrition Lab, Human Clinical Research Facility, Texas A&M University, College Station, TX, 77446, USA; ^2^Institute for Obesity and Program Evaluation, Texas A&M University, College Station, TX, 77446, USA; ^3^Nutrabolt, Bryan, TX, 77446, USA

##### **Correspondence:** Richard B Kreider (rbkreider@tamu.edu)

**Background**

This study examined the short-term effects of ingesting a ready-to-drink pre-workout supplement (RTD) on muscular performance.

**Methods**

Resistance-trained participants (n=25) ingested in a randomized, double-blind, crossover manner a dextrose placebo (PLA, 12g) and RTD containing caffeine (200mg), β-alanine (2.1g), niacin (65mg), folic acid (325mcg), Vitamin B12 (45-mcg), and arginine nitrate (1.3g) for 7d interspersed by a 7d washout. Participants performed a 1RM and 3 x 10 repetitions at 70% 1RM on the bench press (BP) and leg press (LP); ingested the assigned supplement, then had BP and LP 1RM’s determined followed by repetitions to failure (RtF) at 70% 1RM on days 1 and 6. A 4-km cycling time trial (TT) was performed on Days 2 and 7.  Data were analyzed by MANOVA adjusted for sex and relative caffeine dose and are presented as mean change, 95% CI’s.

**Results**

Acute RTD ingestion increased BP lifting volume (70.4, CI 20, 120; PLA 1.96, CI -48, 52 kg). After 6d, both groups increased BP lifting volume (RTD: 108, CI, 59, 157; PLA: 102, CI 54, 152 kg) while LP lifting volume (900, CI 169, 1,632; PLA -968, CI -2,558, 623 kg) and combined lifting volume (1,008, CI 262, 1,755; PLA 383, CI -362, 1,130 kg) were increased in RTD but not PLA. Post-supplementation BP 1RM decreased in both groups at Day 1 (RTD: -2.6, CI, -4.0, -1.2; PLA: -4.4, CI -5.8, -3.0 kg) and Day 6 (RTD: -2.8, CI -4.1, -1.6; PLA: -1.8, CI -3.1, -0.57 kg). No change was observed in LP 1RM for RTD while PLA significantly decreased at Day 1 (RTD: 1.7, CI, -11.1, 14.6; PLA: -15.3, CI -28.2, -2.4 kg) and Day 6 (RTD: -7.4, CI -25.5, 13.8; PLA: -23.8, CI -45, -2.6 kg). RtF increased after acute RTD ingestion (RTD: 2.0, CI, 0.8, 3.3; PLA: -0.4, CI -1.6, 0.9) while both groups improved following 6-d (RTD: 2.8, CI, 1.7, 4.0; PLA: 2.1, CI 1.0, 3.3) TT performance from Day 2 to 7 improved with PLA with no differences between treatments (RTD: -5.72, CI -15.47, 4.0; PLA: -11.48, CI -21.23, -1.73 sec; RTD: 8.1, CI -4.4, 20.7; PLA: 18.2, CI, 5.7, 30.8 W).

**Conclusions**

Acute RTD use enhanced lifting volume while short-term RTD supplementation improved muscular endurance (RtF and lifting volume) and maintained LP 1RM strength but had no effects on 4km cycling time trial performance in resistance-trained college-aged participants.

**Acknowledgements**

This study was supported by Nutrabolt (*Bryan, TX*) through an unrestricted research grant provided to Texas A&M University. CP Earnest serves as a Director of Clinical Sciences for Nutrabolt. RB Kreider served as a university approved scientific advisor for Nutrabolt. PS Murano serves as quality assurance supervisor.

## A79 Short-term effects of a ready-to-drink pre-workout beverage on hematological response to postural challenge

### Patrick B. Collins^1^, Ryan J. Sowinski^1^, Conrad P. Earnest^1^, Ryan L. Dalton^1^, Tyler J. Grubic^1^, Brittany K. Sanchez^1^, Christopher J. Favot^1^, Aimee G. Reyes^1^, Adriana M. Coletta^1^, Christopher Rasmussen^1^, Mike Greenwood^1^, Peter S. Murano^2^, Richard B. Kreider^1^

#### ^1^Exercise and Sport Nutrition Lab, Human Clinical Research Facility, Texas A&M University, College Station, TX, 77446, USA; ^2^Institute for Obesity and Program Evaluation, Texas A&M University, College Station, TX, 77446, USA; ^3^Nutrabolt, Bryan, TX, 77446, USA

##### **Correspondence:** Richard B. Kreider (rbkreider@tamu.edu)

**Background**

Nitrates have been claimed to cause perturbations in blood pressure response, specifically increasing the risk of orthostatic hypotension. The purpose of this study was to investigate the effects of short-term ingestion of a ready-to-drink pre-workout supplement (PWS) on hemodynamic response to changes in posture.

**Methods**

Resistance-trained participants (n=25) ingested in a randomized, double-blind, crossover manner a: (1) Dextrose placebo (PLA, 12g) and, (2) PWS containing caffeine (200mg), β-alanine (2.1g), niacin (65mg), folic acid (325mcg), Vitamin B12 (45mcg), and arginine nitrate (1.3g) in a randomized, crossover manner for 7d, interspersed by 7d washout. We employed a tilt table protocol as a postural challenge to assess hemodynamics. Hemodynamics were analyzed by MANOVA adjusted for sex and relative caffeine dose presented as mean change from baseline (95% CI).

**Results**

Prior to acute supplementation, HR increased from supine to standing for both groups (PWS: 8.25, 95% CI, 4.7, 12; PLA: 8.9, 95% CI, 5.4, 12.5 bpm) and the same occurred at Day 6 (PWS: 7.1, 95% CI, 3.1, 11.1; PLA: 10.8, 95% CI, 6.9, 14.8 bpm). Only PLA increased HR post-ingestion at Day 1 (6.4, 95% CI, 2.9, 9.9 bpm) and Day 6 (9.3, 95% CI, 5.7, 13 bpm) while no changes were seen following PWS ingestion (Day 1: 2.4, 95% CI, -1.2, 6 bpm; Day 6: 3.2, 95% CI, -0.5, 6.9 bpm). SBP increased from supine to standing following PLA ingestion on Day 6 (2.1, 95% CI, 0.05, 4.1 mmHg) with no differences seen with PWS (0.5, 95% CI, -1.6, 2.6 mmHg). No other significant changes were seen with SBP. DBP also increased in the PLA treatment from supine to standing at Day 6 pre-ingestion (2.9, 95% CI, 1.1, 4.7 mmHg) with no change seen following 6d of PWS ingestion (0.8, 95% CI, -1, 2.7 mmHg). Mean arterial pressure was increased after ingestion of the PLA on Day 6 (14.3, 95% CI, 7.3, 21.2 mmHg) with no change following PWS ingestion (5.2, 95% CI, -1.8, 12.3 mmHg). No significant changes were seen in rate pressure product responses for either group at any time point.

**Conclusions**

Results indicate that use of this PWS prior to exercise for up to 7d does not affect heart rate or blood pressure responses to a hemodynamic challenge in resistance-trained males and females.

**Acknowledgements**

This study was supported by Nutrabolt (*Bryan, TX*) through an unrestricted research grant provided to Texas A&M University. CP Earnest served as a Director of Clinical Sciences for Nutrabolt. RB Kreider serves as a university approved scientific advisor for Nutrabolt. PS Murano served as quality assurance supervisor.

